# Efficacy of Silver Diamine Fluoride on Young Children With Severe Early Childhood Caries: A Randomized Clinical Trial

**DOI:** 10.1001/jamapediatrics.2026.2567

**Published:** 2026-07-27

**Authors:** Margherita Fontana, Amr Moursi, Carlos Gonzalez-Cabezas, Divya Khera, Steven M. Levy, George J. Eckert, Barry P. Katz, Livia M. A. Tenuta, Marcia S. Campos, Elisabeta Karl, James Boynton, Courtney Chinn, Alex Sheen, Justine Kolker, John Warren, Emily Thorpe

**Affiliations:** 1 University of Michigan, Ann Arbor; 2 New York University, New York City; 3 University of Iowa, Iowa City; 4 Indiana University, Indianapolis; 5 University of Minnesota, Minneapolis; 6 Maquette University, Milwaukee, Wisconsin

## Abstract

**Importance:**

Dental caries is a highly prevalent yet preventable disease affecting more than 40% of US children, with severe early childhood caries (S-ECC) leading to serious health and economic consequences. This randomized clinical trial addresses critical evidence gaps by evaluating 38% silver diamine fluoride (SDF) effectiveness for caries arrest in young US children with S-ECC to inform US Food and Drug Administration (FDA) regulatory approval.

**Objective:**

To assess the efficacy of 38% SDF vs placebo for arresting active cavitated dentin caries lesions in the primary dentition of US children with S-ECC.

**Design, Setting, and Participants:**

This phase III, multisite, randomized, blind, placebo-controlled clinical trial with 2 parallel arms had a data collection period from October 2018 through April 2023. Analyses were completed in 2024. Three sites recruited from and treated children in a variety of settings: medical and dental clinics, and Head Start and city/state-subsidized preschool programs. Enrolled participants included 830 generally healthy children aged 12 to 71 months with S-ECC and active cavitated lesions.

**Intervention:**

Children were randomized to receive either 38% SDF or placebo, applied at baseline and at 6 months. Clinical examinations were conducted at baseline, 3 months, 6 months, and 8 months.

**OUTCOMES AND MEASURES:**

The primary outcome was the proportion of arrested lesions at 6 months after 1 application. Secondary outcomes included arrest efficacy at 3 months and after 2 applications (8 months), and pain at or before 6 months and 8 months

**Results:**

Of 830 enrolled children, 584 (70%) completed the study. Of those lost to follow-up (approximately 30%), 81 (9.8%) were lost due to COVID-19. Participants included 428 females (52%) and 402 males (48%) and were aged 1 to 6 years with high caries experience (mean [SD] decayed, missing, and filled teeth [dmft] (International Caries Detection and Assessment System ≥1), 11.36 [4.56]; range, 1-23). In intent-to-treat analyses, SDF arrested 57.5%, 54.0%, and 50.2% of lesions at 3 months, 6 months, and 8 months, respectively, vs placebo (18.8%, 22.5%, 17.4%, respectively). Differences (99.9% CI) in arrest rates were 38.7% (28.7%-48.6%), 31.5% (21.5%-41.6%), and 32.8% (22.3%-43.2%). There were no differences in pain. Averse events (AEs) were reported by 196 (47.3%) participants in the SDF group and 180 (43.3%) in the placebo group. Most AEs were mild to moderate in severity; 4 AEs (0.6%) were severe. Treatment-related AEs were similar between the 2 groups (SDF = 22.9%; placebo = 22.2%), as were discontinuation rates due to AEs (7.5% for both groups).

**Conclusion and Relevance:**

In this randomized clinical trial, 38% SDF was significantly more effective than placebo for arresting dentin caries lesions in primary teeth in young children with S-ECC, providing a noninvasive treatment for young high-risk children and supporting its consideration for FDA drug approval.

**Trial Registration:**

ClinicalTrials.gov Identifier: NCT03649659

## Key Points

**Question:**

To inform US Food and Drug Administration regulatory approval, what is the clinical effectiveness of 38% silver diamine fluoride (SDF) when used in medical, dental, and school settings in arresting cavitated dentin caries lesions in the primary dentition of children with severe early childhood caries (S-ECC) at a 6-month follow-up?

**Findings:**

In this blinded, randomized clinical trial of 830 children with S-ECC, 38% SDF demonstrated significantly greater efficacy than placebo in arresting cavitated dentin caries lesions.

**Meaning:**

These results demonstrate that application of 38% SDF was an effective intervention for arresting dentin caries lesions in the primary teeth of young children with S-ECC at a 6-month interval.

## Teaser

This randomized clinical trial assesses the efficacy of 38% silver diamine fluoride vs placebo for arresting active cavitated dentin caries lesions in the primary dentition of US children with severe early childhood caries.

## Introduction

Oral health is an integral component of children’s health, influencing nutrition, growth, development, and quality of life.^1,2^ Dental caries is highly prevalent, affecting nearly half of preschoolers globally and more than 40% of US children ages 2 to 19 years.^2,3^ Untreated caries can cause pain, infection, tooth loss, and complications, such as school absenteeism^4^ and substantial health care costs. Severe early childhood caries (S-ECC) is particularly aggressive and costly,^5^ and often requires repeated treatment under general anesthesia, with added risks, expenses, and possible neurological effects.^2^

Despite this substantial burden, many children—especially those in underserved communities—have limited access to dental care, but more frequent interactions with medical professionals.^6^ Pediatricians and primary care clinicians are key partners in caries detection, prevention, and management.^1,7^ The US Preventive Services Task Force recommends topical fluoride varnish and dietary fluoride supplementation for caries prevention in primary care,^8^ citing insufficient evidence for other interventions.

Affordable and easily applied, 38% silver diamine fluoride (SDF) has emerged as a promising caries-arrest agent.^9,10^ The World Health Organization added SDF to its essential medicines list in 2021.^11^ SDF has been used internationally since the 1970s for caries arrest and sensitivity reduction. In the US, SDF was US Food and Drug Administration (FDA)–cleared in 2014 for adult hypersensitivity; caries use (including pediatrics) is off-label. Nevertheless, both the American Academy of Pediatrics (AAP) and the American Academy of Pediatric Dentistry (AAPD) have issued SDF guidance,^12,13^ and pediatric use has increased, especially post-COVID-19.^14^ SDF entered medical settings with a dedicated 2023 procedural code and inclusion in Smiles for Life training.^15^

38% SDF promotes remineralization, inhibits microbial activity, and hardens carious tissue—arresting decay with characteristic dark staining.^16^ Systematic reviews report caries-arrest rates of 60% to 90% after repeated applications, supporting guideline recommendations.^17,18^ However, a 2024 Cochrane review highlights low certainty for SDF efficacy in primary dentition,^19^ with limited data for children younger than 3 years with S-ECC and none from US populations. In 2016, the FDA granted SDF breakthrough therapy status for caries arrest, motivating trials in children with S-ECC. This randomized clinical trial (RCT) was designed to support an FDA caries-arrest drug claim in this population. The primary objective was 6-month efficacy of 1 SDF application to arrest active cavitated caries dentin lesions in vital teeth. Secondary outcomes include lesion arrest at 3 months and at 8 months after reapplication at 6 months, and lesion pain at or before 6 months and 8 months. Oral health–related quality of life and treatment satisfaction and acceptability were also assessed (reported elsewhere).^20^

## Methods

### Study Design

Funded by the National Institute of Dental and Craniofacial Research (NIDCR), this phase III, multisite, randomized, blinded, placebo-controlled trial compared 2 parallel groups, SDF (Advantage Arrest; Elevate Oral Care) vs placebo (deionized water with blue 1 dye; Elevate Oral Care) over 8 months.

The hypothesis that SDF is superior to placebo was tested by measuring changes in dentin hardness using the International Caries Detection and Assessment System (ICDAS) criteria, focusing on cavitated lesions (ICDAS 5: less than half surface or 6: half or more of surface).^21^ Study product was applied at baseline and 6 months, with in-person visits at baseline, 3 months, 6 months, and 8 months (plus or minus 10 days). Adverse events (AEs), severe AEs (SAEs), and unanticipated problems (UPs) were assessed in person within 48 hours postapplication during in-person visits and intermediate checks at approximately 1.5 months, 4.5 months, and 7 months.

Each site used 2 or more calibrated dentists with annual cross-site ICDAS calibration; reliability required κ more than 0.75 (ICDAS) and more than 0.70 (for both lesion hardness [soft vs hard] and color [yellow/brown vs black]). The study followed Consolidated Standards of Reporting Trials (CONSORT) reporting guidelines, was approved by WCG institutional review board, and obtained written informed consent; a NIDCR-appointed data safety monitoring board (DSMB) monitored trial integrity. The trial was registered at ClinicalTrials.gov (NCT03649659). See Supplement 1 for trial protocol.

### Participants

From 2018 to 2022, the University of Michigan, New York University, and the University of Iowa teams recruited children aged 12 to 71 months via medical/dental private practice and academic clinics, Head Start, preschools, and online. Online recruits completed assessments and were treated at a convenient participating clinic. In Michigan and Iowa, in-person recruits were assessed and treated at the same site. In New York, school-based recruits completed visits in an academic dental clinic. Eligibility required S-ECC, 1 or more accessible soft cavitated lesions (ICDAS 5-6) suitable for SDF, and sufficient cooperation. Children with developmental or intellectual disabilities were included if they met other criteria.

Parents/legal guardians (≥18 years) or emancipated minors provided consent; foster care children were excluded. Parents/guardians had to comply with procedures and read English, Spanish, or Mandarin.

Exclusion criteria included pain from caries; pulpal exposure; evidence of pulpal infection (abscess, fistula, swelling); nonexfoliative tooth mobility; gingival or peri-oral ulceration, abscess, or stomatitis; allergy or sensitivity to silver/heavy metals; expected inability to comply with protocols; specified chronic diseases or conditions (osteopenia, osteoporosis, chronic kidney disease, amelogenesis or dentinogenesis imperfecta, hypothyroidism, hyperparathyroidism, hypocalcemia); or ongoing use of chronic glucocorticoids, anticonvulsants, chemotherapy, or bisphosphonates.

### Randomization and Intervention

Participants were randomized 1:1 to receive either 38% SDF or placebo, stratified by study site, using random block sizes of 4/6 generated by SAS version 9.4 (SAS Institute). Randomization occurred at the participant level following eligibility confirmation. Blinding used identical coded unit-dose ampules with matched liquid color. An unmasked biostatistician generated assignments; the project biostatistician (G.E.) remained blinded until database lock.

Following randomization, SDF/placebo was applied without caries removal. Lesions were cleaned (toothbrush/microbrush), dried (cotton/gauze), and treated for approximately 10 seconds using a standardized applicator, then blotted dry. No fluoride varnish or other topical fluoride was administered on the day of treatment. Trained hygienists applied product, separate from masked examiners, to maintain blinding.

### Data Collection

At each visit, full-mouth soft tissue and ICDAS examinations assessed hardness of all cavitated lesions. The primary outcome (lesion hardness) was assessed independently from color. AEs, SAEs, and UPs were monitored at each visit and intermediate contact.

### Outcomes

Arrest of cavitated lesions was evaluated using ICDAS II activity criteria. The primary end point was the proportion of trial lesions arrested at 6 months after 1 application. Secondary end points included arrest at 3 months and at 8 months following a second application at 6 months. Arrest was binary (yes/no) at the lesion level. Study teeth with pain, pulpal exposure, abnormal mobility, or infection exited early and were referred; lesions on these teeth were failures. AEs were tracked from consent through 7 days posttrial and SAEs were tracked through 30 days.

Dental pain was assessed using the validated parent/guardian-reported Dental Discomfort Questionnaire (DDQ) for children younger than 5 years.^22^ This includes a toothache occurrence/timing item (0 = none) and 8 caries/toothache-related behavior items scored 0 = never, 1 = sometimes, 2 = often (sum 0-16; higher = worse). Children with a DDQ score of 1 or higher underwent clinical examination to confirm trial tooth pain; any postbaseline trial-tooth pain was recorded as trial lesion pain.

### Sample Size and Statistical Analysis

Target enrollment was 1144 (80% power to detect 55% vs 45% arrest at α = .001), accounting for clustering (participant as cluster with multiple lesions; within-participant correlation 0.5), and an early stopping rule by interim analyses after approximately half the sample completed the 6-month visit (Lan-Demets O’Brien-Fleming boundaries)^23^ with efficacy/futility thresholds. Calculations used East version 6 (Cytel). Additional details are provided in the statistical analysis plan (Supplement 2).

After a positive interim analysis, the DSMB/NIDCR approved early termination for efficacy (September 2022) and the final number enrolled was 830.^24^ Already enrolled participants could complete follow-up.

Per FDA requirements, between-groups tests used an α = .001 for arrest at 6 months (primary) and for the hierarchical testing strategy of the 2 secondary outcomes, arrest at 8 months followed by pain at 8 months if arrest at 8 months was significant. The prespecified within-group change from 3 months to 6 months used a less stringent significance level .05. Arrest at 3 months and pain at 6 months were exploratory outcomes. Multiple imputation addressed missing data; 25 imputed datasets were analyzed and combined using standard methods (Supplement 2).

Between-group arrest at 6-month was analyzed using a generalized estimating equation (GEE) model with logit link, adjusting for site; lesions were the unit of analysis with clustering of multiple lesions within a participant. Similar GEE models analyzed arrest at 3 months and 8 months, as well as lesion pain at or before the 6-month and 8-month visits. A longitudinal binary GEE incorporated within-participant correlations across lesions and time points (3 months and 6 months). No statistical tests were performed for AE outcomes. Statistical analyses used SAS version 9.4 (SAS Institute).

## Results

From September 2018 to September 2022, 830 participants were randomized (Figure): 414 in the SDF group (976 lesions) and 416 in the placebo group (1011 lesions); all were included in the intent-to-treat analyses. COVID-19 affected follow-up (Figure); attrition was 29.6%, including 9.8% impacted at the final visit. Participants were diverse, aged 1 to 6 years with high caries experience (mean [SD] decayed, missing, and filled teeth [dmft] (ICDAS ≥1), 11.36 [4.56]; range, 1-23). The dmft index summarizes caries experience in the 20 primary teeth. A mean dmft of 11 indicates approximately 11 of 20 primary teeth affected (high disease burden). Baseline characteristics and behaviors are summarized in Table 1 and eTable 1 in Supplement 3. All examiners met and maintained annual calibration thresholds for ICDAS scores, lesion hardness, and color.

**Figure.**
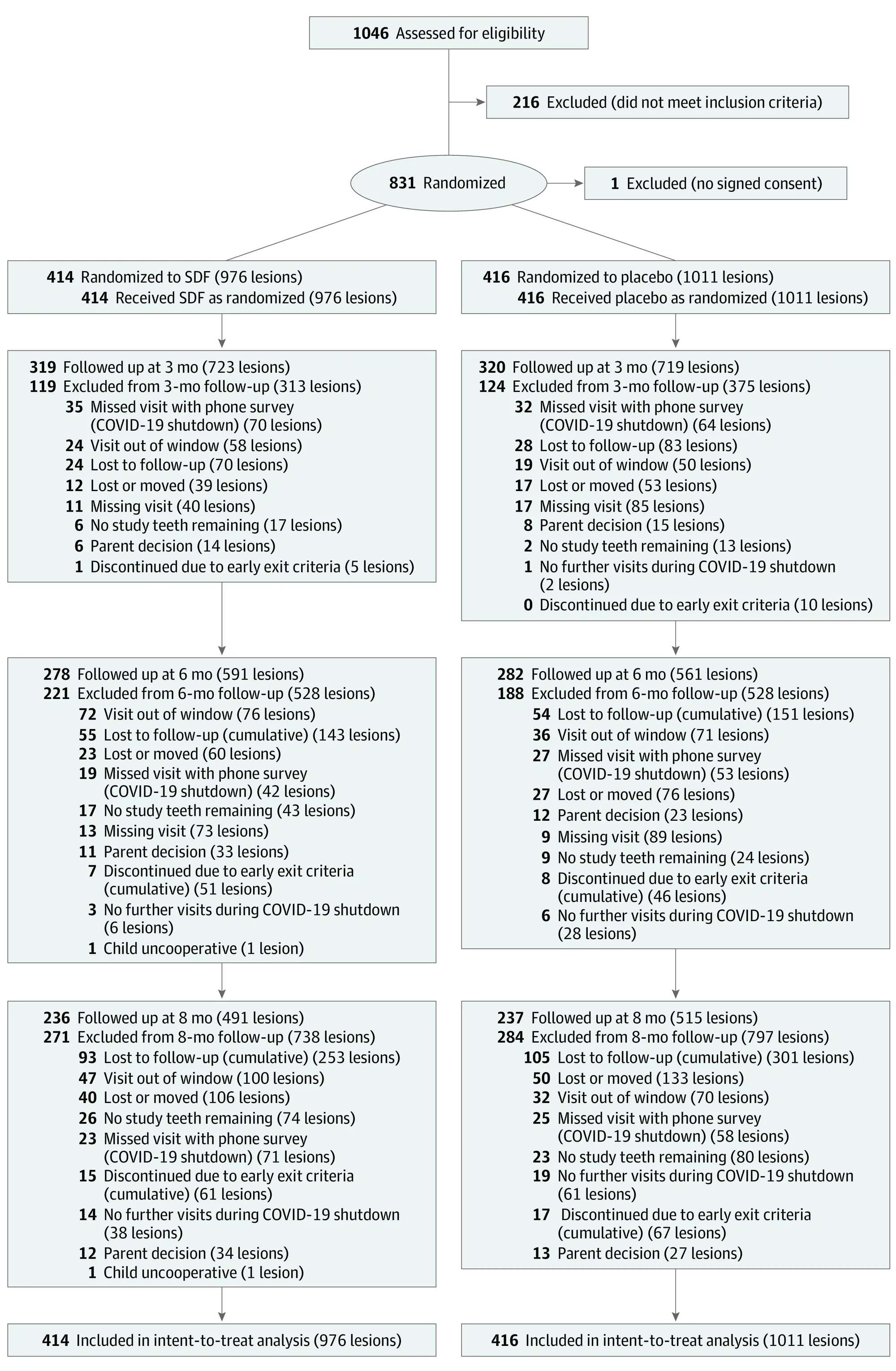
Consolidated Standards of Reporting Trials (CONSORT) Flow Diagram SDF indicates silver diamine fluoride.

**Table 1.** Participant Characteristics at Baseline

Variable	Level	No. (%)		
		SDF (n = 414)	Control (n = 416)	Overall (n = 830)
Child age, y	1	15 (3.6)	19 (4.6)	34 (4.1)
	2	56 (13.5)	56 (13.5)	112 (13.5)
	3	124 (30.0)	107 (25.7)	231 (27.8)
	4	155 (37.4)	170 (40.9)	325 (39.2)
	5	64 (15.5)	64 (15.4)	128 (15.4)
Child’s sex	Female	228 (55.1)	200 (48.1)	428 (51.6)
	Male	186 (44.9)	216 (51.9)	402 (48.4)
Child race and ethnicity^a^	Black or African American	67 (16.2)	73 (17.5)	140 (16.9)
	Hispanic or Latino	182 (44.0)	183 (44.0)	365 (44.0)
	Multiracial	21 (5.1)	28 (6.7)	49 (5.9)
	White	106 (25.6)	96 (23.1)	202 (24.3)
	Other^b^	27 (6.5)	24 (5.8)	51 (6.1)
	Not reported	11 (2.7)	12 (2.9)	23 (2.8)
Study site	New York University	203 (49.0)	203 (48.8)	406 (48.9)
	University of Iowa	12 (2.9)	12 (2.9)	24 (2.9)
	University of Michigan	199 (48.1)	201 (48.3)	400 (48.2)
Primarily non-English speaking at home	NA	0	3 (0.7)	3 (0.4)
	No	195 (47.1)	209 (50.2)	404 (48.7)
	Yes	219 (52.9)	204 (49.0)	423 (51.0)
Household income	Missing	16	7	23
	<$5000	41 (10.3)	38 (9.3)	79 (9.8)
	$5000-$9999	22 (5.5)	37 (9.0)	59 (7.3)
	$10 000-$19 999	41 (10.3)	36 (8.8)	77 (9.5)
	$20 000-$29 999	42 (10.6)	42 (10.3)	84 (10.4)
	$30 000-$39 999	35 (8.8)	26 (6.4)	61 (7.6)
	$40 000-$49 999	23 (5.8)	27 (6.6)	50 (6.2)
	$50 000-$79 999	23 (5.8)	34 (8.3)	57 (7.1)
	$80 000-$99 999	3 (0.8)	8 (2.0)	11 (1.4)
	$100 000 or more	13 (3.3)	14 (3.4)	27 (3.3)
	Do not know/missing	155 (38.9)	147 (35.9)	302 (37.4)

Abbreviations: NA, not applicable; SDF, silver diamine fluoride.

^a^ Race and ethnicity were caregiver-reported.

^b^ Includes American Indian or Alaskan Native, Asian, and Native Hawaiian or Other Pacific Islander.

Lesions were evenly split anterior/posterior; 68.1% were maxillary, 62.7% ICDAS 5, and 73.6% yellow. There were no significant differences in lesion characteristics between groups (Table 2).

**Table 2.** Baseline Trial Lesion Characteristics

Variable	Level	No. (%)		
		SDF (n = 976)	Control (n = 1011)	Overall (n = 1987)
Tooth location	Anterior	477 (48.9)	522 (51.6)	999 (50.3)
	Posterior	499 (51.1)	489 (48.4)	988 (49.7)
Jaw	Mandibular	311 (31.9)	323 (31.9)	634 (31.9)
	Maxillary	665 (68.1)	688 (68.1)	1353 (68.1)
ICDAS severity at baseline	5	623 (63.8)	622 (61.5)	1245 (62.7)
	6	353 (36.2)	389 (38.5)	742 (37.3)
Lesion color at baseline	Black	24 (2.5)	17 (1.7)	41 (2.1)
	Brown	243 (24.9)	238 (23.5)	481 (24.2)
	Not visible	1 (0.1)	2 (0.2)	3 (0.2)
	Yellow	708 (72.5)	754 (74.6)	1462 (73.6)

Abbreviations: ICDAS, International Caries Detection and Assessment System; SDF, silver diamine fluoride.

Most children had all study lesions treated at each visit with a single ampule/dose (eTable 2 in Supplement 3). The SDF group had significantly higher proportions of arrested lesions than the control at all visits (3-month, 6-month, and 8-month) (Table 3). In this high caries risk cohort, at 6 months after 1 application, 22.6% (95% CI, 15.3%-31.8%) of control lesions arrested naturally, while 54.0% (95% CI, 43.1%-64.6%) arrested in the SDF group. Lesion arrest in the control group remained unchanged between 3 months and 6 months (odds ratio [OR], 1.21; 95% CI, 0.93-1.58; *P* = .15), whereas arrest in the SDF group decreased slightly but significantly between 3 months and 6 months (OR, 0.83; 95% CI, 0.69-1.00; *P* = .05) and the absolute decrease was less than 5%.

**Table 3.** Lesion Arrest Data at 6 Months and 3-Month and 8-Month Follow-Up and Presence of Lesion Pain at or Before 6-Month and 8-Month Follow-Up

Visit	% (99.9% CI)		Odds ratio SDF to control (99.9% CI)	*P* value^a^	Risk difference SDF—control, % (99.9% CI)
	SDF^b^	Control			
Lesion arrest					
6 mo (Primary)	54.0 (43.1 to 64.6)	22.5 (15.3 to 31.8)	4.06 (2.54 to 6.48)	<.001	31.5 (21.5 to 41.6)
3 mo (Exploratory)	57.5 (46.5 to 67.8)	18.8 (12.4 to 27.5)	5.84 (3.59 to 9.51)		38.7 (28.7 to 48.6)
8 mo (Secondary)	50.2 (39.5 to 60.9)	17.4 (11.4 to 25.7)	4.78 (2.84 to 8.05)		32.8 (22.3 to 43.2)
Pain					
6 mo (Exploratory)	1.3 (0.3 to 5.7)	1.3 (0.3 to 5.8)	0.96 (0.18 to5.12)	.89	0 (−2.2 to 2.1)
8 mo (Secondary)	2.7 (0.8 to 8.9)	2.8 (0.8 to 9.6)	0.95 (0.25 to 3.54)		−0.2 (−3.6 to 3.3)

Abbreviation: SDF, silver diamine fluoride.

^a^ Multiply imputed data analyzed using generalized estimating equation models with logit link, adjusting for site; lesions were the unit of analysis with clustering of multiple lesions within a participant.

^b^ Tests used α = .001 for arrest at 6 months (primary) and for the hierarchical testing strategy of 2 secondary outcomes, arrest at 8 months followed by pain at 8 months if arrest at 8 months was significant. Arrest at 3 months and pain at 6 months were exploratory outcomes.

Exploratory analyses concluded that at 6 months, posterior lesions arrested less than anterior lesions (25.8% vs 41.6%; OR, 0.49; 95% CI, 0.35-0.67). In addition, at 8 months, baseline ICDAS 5 lesions had lower arrest rates than ICDAS 6 lesions (24.7% vs 31.3%; OR, 0.72; 95% CI, 0.52-0.98), posterior lesions had a lower arrest percentage than anterior lesions (20.2% vs 37.1%; OR, 0.43; 95% CI, 0.30-0.61), and sites differed (University of Iowa, <14.9%; University of Michigan, <30.2%; New York University, <43.1%).

There were no statistically significant differences between groups for the presence of lesion pain at or before the 8-month or 6-month follow-up visits (Table 3). AEs were reported by 196 participants (47.3%) in the SDF group and 180 (43.3%) in the placebo group. Table 4 summarizes AEs, SAEs, and UPs. Most AEs were mild to moderate in severity; 4 AEs (0.6%) were severe. Treatment-related AEs were similar between the 2 groups (SDF = 22.9%; placebo = 22.2%), as were discontinuation rates due to AEs (7.5% for both groups).

**Table 4.** Safety Data of Adverse Events Information

Variable	Response	No. (%)	
		SDF (n = 414)	Control (n = 416)
Any adverse event		196 (47.3)	180 (43.3)
Adverse event term^a^			
Abscess		35 (8.5)	34 (8.2)
Allergic reaction		3 (0.7)	3 (0.7)
Constipation		3 (0.7)	3 (0.7)
Cough		9 (2.2)	7 (1.7)
Dental trauma		6 (1.4)	5 (1.2)
Dental treatment		38 (9.2)	33 (7.9)
Diarrhea		6 (1.4)	5 (1.2)
Ear infection		11 (2.7)	9 (2.2)
Extraoral staining		10 (2.4)	0
Fever		15 (3.6)	25 (6.0)
Gastrointestinal pain		9 (2.2)	12 (2.9)
Intraoral staining		5 (1.2)	1 (0.2)
Nausea		1 (0.2)	2 (0.5)
Oral erythema		4 (1.0)	2 (0.5)
Oral pain		3 (0.7)	4 (1.0)
Oral ulceration		2 (0.5)	6 (1.4)
Other		29 (7.0)	19 (4.6)
Other surgical procedure		3 (0.7)	5 (1.2)
Pulpal exposure		9 (2.2)	16 (3.8)
Respiratory infection		11 (2.7)	12 (2.9)
Skin and tissue disorders		10 (2.4)	4 (1.0)
Sore throat		4 (1.0)	2 (0.5)
Toothache		62 (15.0)	51 (12.3)
Vomiting		6 (1.4)	17 (4.1)
System Organ Class^b^			
Congenital, familial, and genetic disorders		0	1 (0.2)
Gastrointestinal disorders		115 (27.8)	123 (29.6)
General disorders and administration site conditions		17 (4.1)	25 (6.0)
Immune system disorders		6 (1.4)	3 (0.7)
Infections and infestations		30 (7.2)	27 (6.5)
Injury, poisoning, and procedural complications		26 (6.3)	10 (2.4)
Musculoskeletal and connective tissue disorders		1 (0.2)	0
Respiratory, thoracic, and mediastinal disorders		15 (3.6)	10 (2.4)
Skin and subcutaneous tissue disorders		12 (2.9)	4 (1.0)
Surgical and medical procedures		41 (9.9)	38 (9.1)
Vascular disorders		0	1 (0.2)
Relationship to treatment	No adverse event	218 (52.7)	236 (56.7)
	Not related	54 (13.0)	48 (11.5)
	Unlikely related	47 (11.4)	40 (9.6)
	Possibly related	41 (9.9)	46 (11.1)
	Probably related	37 (8.9)	32 (7.7)
	Related	17 (4.1)	14 (3.4)
Severity CTCAE grade	No adverse event	218 (52.7)	236 (56.7)
	Mild	84 (20.3)	64 (15.4)
	Moderate	108 (26.1)	116 (27.9)
	Severe	4 (1.0)	0
	No	383 (92.5)	385 (92.5)
Caused study discontinuation	Yes	31 (7.5)	31 (7.5)
	No	404 (97.6)	412 (99.0)
Adverse event related to COVID-19	Yes	10 (2.4)	4 (1.0)
	No	411 (99.3)	415 (99.8)
Is the adverse event serious?	Yes	3 (0.7)	1 (0.2)

Abbreviations: CTCAE, Common Terminology Criteria for Adverse Events; SDF, silver diamine fluoride.

^a^ Terms derived from the CTCAE version 5.0.

^b^ A System Organ Class in the Medical Dictionary for Regulatory Activities is the highest-level grouping for adverse event terms, categorizing them by body system (eg, cardiac disorders), etiology (eg, infections), or purpose (eg, surgical procedures) to standardize medical coding, data retrieval, and analysis in pharmacovigilance and clinical trials.

## Discussion

In this RCT, we evaluated the efficacy and safety of 38% SDF in young children with S-ECC across diverse settings, including medical, dental, and school environments. Consistent with school-based studies,^25,26^ SDF may also be feasible in primary care because application is brief and noninvasive. Generalizability depends on primary care teams reliably identifying eligible cavitated lesions, supported by training, protocols, and referral pathways.

Our results show that 38% SDF confers substantial, statistically, and clinically significant benefits to high caries-risk young children. The study was designed to meet FDA requirements for a caries-arrest drug application. In the US, over-the-counter fluoride products are regulated as drugs for dental caries,^27^ while most high-concentration professional fluoride products, including 38% SDF and 5% fluoride varnish, enter the market as medical devices to manage dentin hypersensitivity in adults. Thus, caries use, especially in children, is off-label.

A 2024 Cochrane review found low-certainty evidence for SDF in the primary dentition,^19^ and limited data in children younger than 3 years with S-ECC, particularly in US populations. Since 38% SDF became available in the US in 2014, several SDF products and 1 nanosilver product have been cleared by the FDA for hypersensitivity. Approval of SDF specifically for caries arrest in young children would represent an important innovation to facilitate safe, effective person-centered dental care, reinforcing the rationale for this RCT.^28^

To address pediatric RCT ethics, children were connected to a dental home for ongoing care. Other professionally applied topical fluoride products are not FDA-approved for caries or pediatric use and have not been shown to arrest cavitated lesions^29,30^; thus, they could not serve as controls per FDA guidance. The trial’s short duration (8 months) and provision for prompt scheduling of urgent visits supported participant safety. The FDA-mandated primary end point at 6 months aligns with routine dental examination intervals. The literature indicates that arrest rates may improve with repeated applications in older children,^17^ so the impact of a second application at the 6-month visit was assessed, extending follow-up to 8 months. The study period was considered ethically appropriate given the often-prolonged wait times for general anesthesia in hospital dental settings,^31^ a common pathway for children with S-ECC. Eight months also overlaps with the length of a school year, facilitating regular follow-up. In-person and intermediate contacts maintained engagement about every 2 months. Postapplication safety monitoring included 24- to 48-hour in-person assessment, surpassing the safety standards of most prior studies, which typically relied on parent/participant reporting. Safety remains paramount in pediatric research, and no differences in AEs were recorded between groups, congruent with previous studies supporting broader clinical use of SDF in young children.

The SDF product evaluated was the only 38% formulation available in the US at the time. Preclinical toxicological data and prior human trials confirmed safety and efficacy for treating adult dentin hypersensitivity and pediatric pharmacokinetic studies further supported the conduct of this trial.^32,33^ Evidence regarding natural caries arrest in this high-risk population is limited. Prior research on preschool children with ECC demonstrated that mean arrested lesion counts in the SDF group were 1.5 to 2.6 times higher than controls,^9,10^ guiding our conservative sample size ratio. Arrest in our sample (approximately 50% after 1 or 2 applications) exceeded reports for 1- to 3-year-olds outside the US (21% at 6 months post-first application; 35% post-second application),^30^ but remained lower than in older preschool children (64to 84%, up to 90% with semiannual/combination treatments).^34^ Lower efficacy in younger children may stem from factors, such as limited cooperation to maintain the cavity dry during application and suboptimal oral health behaviors (eg, nighttime bottle feeding and delayed tooth brushing).^30^

Our data did not demonstrate incremental improvement in arrest rates following a second SDF application. We also found no significant SDF–placebo pain differences at 6 months or 8 months, possibly because not all lesions arrested in this high-risk population. Reapplication timing matched guidelines,^12,18^ and the 2-month interval between the second dose and final assessment should suffice, given lesion arrest typically manifests within 2 to 3 weeks after treatment.^32^ Our 3-month study data confirmed early arrest. Nevertheless, more frequent application schedules may be beneficial in high-risk populations. A recent Canadian study^35^ observed nearly complete arrest rates in 1-month and 4-month reapplication interval groups, but lowest arrest with a 6-month interval. The current study protocol incorporated measures to minimize bias and uphold data quality; for example, hardness and color were assessed separately because color is an imperfect arrest marker. In prior work, Chu et al^9^ found that, while 100% of SDF-treated lesions darkened, so did a significant proportion of fluoride varnish–treated and untreated lesions.

Even without intervention, about 20% of lesions in the control group arrested naturally. Placebo/no-treatment SDF trials show spontaneous arrest occurrs.^9,10^ Additionally, nonrestorative caries control—in which lesions are mechanically opened to support self-cleaning—has shown efficacy in arresting cavitated caries.^36,37^ Consistent with prior work, arrest was higher for anterior and more advanced lesions, likely due to easier hygiene access.^38^ These anatomical factors likely contribute to the sustained effectiveness of SDF and similar interventions.

### Limitations

Despite these encouraging findings, several limitations must be acknowledged. First, the study sample, though diverse, is not representative of all US pediatric groups. Second, while sufficient for primary end points, the trial duration does not capture potential long-term effects. Third, the COVID-19 pandemic contributed to loss to follow-up; however, this was mitigated by intention-to-treat analysis. Measures were taken to reduce bias, yet the color change associated with SDF may have occasionally compromised blinding. While the trial focused on data for an FDA drug claim for caries arrest, future research should address alternate follow-up intervals, evaluate arrest of noncavitated lesions by surface, assess preventive effects, and elucidate mechanistic and population-specific outcomes. Additionally, the high rate of nonarrested lesions in this high-risk cohort underscores the need to use SDF alongside other preventive strategies, rather than relying on it alone.

## Conclusions

This clinical trial of young children with S-ECC demonstrated that 38% SDF achieves more than twice the arrest rate of placebo, representing a statistically and clinically significant difference, with no lasting adverse effects after 1 or 2 applications. The data also suggest that, since not all lesions arrest, and there are no differences in pain, close monitoring of these children is needed. These results substantiate the FDA’s Breakthrough Therapy designation and provide essential data supporting evaluation of SDF drug-claim approval for caries arrest in children aged 1 to 6 years with S-ECC.

## Notes

Supplement 1. Trial protocol

Supplement 2. Statistical analysis plan

Supplement 3. eMethods 1. Sample size and early stopping rules eMethods 2. Multiple imputation eTable 1. Participant Dental Characteristics and Practices at Baseline eTable 2. Product Application-Number of Treatment Doses/Ampules Used

Supplement 4. Data sharing statement
