# Supplementary material for: Efficacy of Silver Diamine Fluoride on Young Children With Severe Early Childhood Caries: A Randomized Clinical Trial
Source: JAMA Pediatr. 2026 Jul 27:e262567. Online ahead of print. doi: 10.1001/jamapediatrics.2026.2567 (PMC13409122; doi:10.1001/jamapediatrics.2026.2567)
Supplement: Supplement 1. — Trial protocol [file jamapediatr-e262567-s001.pdf]

Phase III RCT of the Effectiveness of Silver Diamine Fluoride in Arresting Cavitated Caries Lesions

NIDCR Protocol Number: 17-094-E

NIDCR Grant Number: U01DE027372

Principal Investigator: Margherita Fontana, DDS, PhD

NIH Project Scientist: Jane Atkinson, DDS

NIDCR Program Official: Dena Fischer, DDS, MSD, MS

NIDCR Medical Monitor: Kevin D. McBryde, MD

IND Sponsor: Advantage Silver Dental Arrest, LLC

Draft or Version Number: 3.1

16 April 2019

STATEMENT OF COMPLIANCE

The trial will be conducted in accordance with the International Conference on Harmonisation guidelines for Good Clinical Practice (ICH E6), the Code of Federal Regulations on the Protection of Human Subjects (45 CFR Part 46), and the NIDCR Clinical Terms of Award. All personnel involved in the conduct of this trial have completed human subjects' protection training.

SIGNATURE PAGE

The signature below constitutes the approval of this protocol and the attachments, and provides the necessary assurances that this trial will be conducted according to all stipulations of the protocol, including all statements regarding confidentiality, and according to local legal and regulatory requirements and applicable US federal regulations and ICH guidelines.
Principal Investigator or Clinical Site Investigator:

Signed: ______________________________ Date: ____________

Name:

Title:

TABLE OF CONTENTS

PAGE

STATEMENT OF COMPLIANCE I
SIGNATURE PAGE II
TABLE OF CONTENTS III
LIST OF ABBREVIATIONS VI
PROTOCOL SUMMARY VIII
1 KEY ROLES AND CONTACT INFORMATION 11
2 INTRODUCTION: BACKGROUND INFORMATION AND SCIENTIFIC RATIONALE 13
2.1 Background Information 13
2.2 Rationale 14
2.3 Potential Risks and Benefits 16
2.3.1 Potential Risks 16
2.3.2 Potential Benefits 17
3 OBJECTIVES 19
3.1 Trial Objectives 19
3.2 Trial Outcome Measures 19
3.2.1 Primary 19
3.2.2 Secondary 19
3.2.3 Tertiary 20
4 TRIAL DESIGN 21
5 TRIAL ENROLLMENT AND WITHDRAWAL 22
5.1 Participant Inclusion Criteria 22
5.2 Participant Exclusion Criteria 22
5.3 Strategies for Recruitment and Retention 23
5.3.1 Recruitment 23
5.3.2 Retention 25
5.4 Treatment Assignment Procedures 25
5.4.1 Randomization Procedures 25
5.4.2 Masking Procedures 25
5.4.3 Breaking the Blind 26
5.5 Participant Withdrawal 26
5.5.1 Reasons for Withdrawal 26
5.5.2 Handling of Participant Withdrawals or Participant Discontinuation of Trial Intervention 26
5.6 Premature Termination or Suspension of Trial 26
6 TRIAL INTERVENTION 28
6.1 Trial Product Description 28
6.1.1 Formulation, Packaging, and Labeling 28
6.1.2 Product Storage and Stability 28
6.2 Dosage, Preparation and Administration of Trial Product 28
6.3 Modification of Trial Product Administration for a Participant 29
6.4 Accountability Procedures for the Trial Product 29
6.5 Concomitant Medications/Treatments 29

7 TRIAL SCHEDULE ... 30
7.1 Consenting Process ... 30
7.2 Screening/Enrollment/Baseline ... 31
7.3 Intermediate Visits ... 31
7.3.1 24- to 48-Hour Safety Contacts (±8 hours) ... 31
7.3.2 24- to 48-Hour Safety Visits (±8 hours) ... 31
7.3.3 1.5-, 4.5-, 7-Month Intermediate Contacts (±10 days) ... 31
7.3.4 Three-Month Visit (±19 days) ... 32
7.3.5 Six-Month Visit (±19 days) ... 32
7.4 Final Trial Visit: 8 Months (±19 days) ... 32
7.5 Withdrawal Visit ... 32
7.6 Unscheduled Visit ... 32
7.7 Unscheduled Contact ... 33
8 TRIAL PROCEDURES / EVALUATIONS ... 34
8.1 Trial Procedures/Evaluations ... 34
8.1.1 Clinical Examination ... 34
8.1.2 Questionnaires ... 34
9 ASSESSMENT OF SAFETY ... 36
9.1 Specification of Safety Parameters ... 36
9.1.1 Adverse Events ... 36
9.1.2 Serious Adverse Events ... 36
9.1.3 Unanticipated Problems ... 37
9.2 Time Period and Frequency for Event Assessment and Follow-Up ... 37
9.3 Characteristics of an Adverse Event ... 37
9.3.1 Relationship to Trial Intervention ... 37
9.3.2 Expectedness of AEs ... 38
9.3.3 Severity of Event ... 38
9.4 Reporting Procedures ... 38
9.4.1 Unanticipated Problem Reporting to IRB and NIDCR ... 38
9.4.2 Serious Adverse Event Reporting to IND Sponsor and NIDCR ... 39
9.4.3 Reporting of Safety Events to FDA ... 39
9.5 Halting Rules ... 40
10 TRIAL OVERSIGHT ... 41
11 CLINICAL SITE MONITORING ... 42
12 STATISTICAL CONSIDERATIONS ... 43
12.1 Trial Hypotheses ... 43
12.2 Sample Size Considerations ... 43
12.3 Planned Interim Analyses ... 44
12.3.1 Safety & Efficacy Review ... 44
12.4 Final Analysis Plan ... 44
12.4.1 Analysis populations ... 46
12.4.2 Missing data ... 46
13 SOURCE DOCUMENTS AND ACCESS TO SOURCE DATA/DOCUMENTS ... 47
14 QUALITY CONTROL AND QUALITY ASSURANCE ... 48

14.1 Participant Binder 48
14.2 Regulatory Binder 48
15 ETHICS/PROTECTION OF HUMAN SUBJECTS 49
15.1 Ethical Standard 49
15.2 Institutional Review Board 49
15.3 Informed Consent Process 49
15.4 Exclusion of Women, Minorities, and Children (Special Populations) 50
15.5 Participant Confidentiality 50
15.6 Future Use of Stored Specimens and Other Identifiable Data 51
16 DATA HANDLING AND RECORD KEEPING 52
16.1 Data Management Responsibilities 52
16.2 Data Capture Methods 52
16.3 Types of Data 52
16.4 Schedule and Content of Reports 52
16.5 Trial Records Retention 53
16.6 Protocol Deviations 53
17 PUBLICATION/DATA SHARING POLICY 54
18 LITERATURE REFERENCES 55
SUPPLEMENTAL MATERIALS 58
APPENDICES 59
APPENDIX A: ICDAS II CRITERIA: CARIES LESION SEVERITY AND ACTIVITY ASSESSMENT60
APPENDIX B: DENTAL DISCOMFORT QUESTIONNAIRE (DDQ) 63
APPENDIX C: ORAL HEALTH-RELATED QUALITY OF LIFE (OHRQOL) QUESTIONNAIRES .. 64
APPENDIX D: FAMILY SURVEY 68
APPENDIX E: ADVERSE EVENTS 71

LIST OF ABBREVIATIONS

AE	Adverse Event/Adverse Experience
CFR	Code of Federal Regulations
CMP	Clinical Monitoring Plan
CRF	Case Report Form
DCCC	Data and Clinical Coordinating Center
DDQ	Dental Discomfort Questionnaire
DMFS	Decayed, missing, and filled tooth surfaces
DSF	Diammine Silver Fluoride
DSMB	Data and Safety Monitoring Board
eCRF	Electronic Case Report Form
ECC	Early Childhood Caries
EHS	Early Head Start
F	Fluoride
F-Toothpaste	Fluoridated Toothpaste
FDA	Food and Drug Administration
FIS	Family Impact Scale
FFR	Federal Financial Report
FV	Fluoride Varnish
GCP	Good Clinical Practice
HIPAA	Health Insurance Portability and Accountability Act
HS	Head Start
ICDAS	International Caries Detection and Assessment System
ICH	International Conference on Harmonisation
ICMJE	International Committee of Medical Journal Editors
IND	Investigational New Drug Application
IRB	Institutional Review Board
IWRS	Interactive Web Response System
LG	Legal Guardian
MedDRA®	Medical Dictionary for Regulatory Activities
MOP	Manual of Procedures
N	Number (typically refers to participants)
NDA	New Drug Application
NIDCR	National Institute of Dental and Craniofacial Research, NIH
NIH	National Institutes of Health

OCTOM	Office of Clinical Trials Operations and Management, NIDCR, NIH
OHRP	Office for Human Research Protections
OHRQoL	Oral Health-Related Quality of Life
OR	Operating Room
P-CPQ	Parental-Caregiver Perception Questionnaire
PHI	Protected Health Information
PI	Principal Investigator
QA	Quality Assurance
QC	Quality Control
RCT	Randomized Clinical Trial
SAE	Serious Adverse Event/Serious Adverse Experience
SAP	Statistical Analysis Plan
SDF	Silver Diamine Fluoride
SECC	Severe Early Childhood Caries
sIRB	Single Institutional Review Board
SOP	Standard Operating Procedure
UP	Unanticipated Problem
US	United States

PROTOCOL SUMMARY

Title: *Phase III RCT of the Effectiveness of Silver Diamine Fluoride in Arresting Cavitated Caries Lesions*

Précis: There are large disparities in the access to and effectiveness of many available preventive strategies for Severe-Early Childhood Caries (S-ECC) and Early Childhood Caries (ECC), two of the disease presentations for dental caries in children, one of the most common chronic diseases of childhood. This inequity frequently results in progression of dental disease in higher risk groups to an advanced stage, during which cavitated carious lesions and/or pulpal infections develop. When primary prevention fails or is not accessible, effective treatments that are safe, simple, low-cost, and acceptable to patients are necessary to decrease disease morbidity.

The product, 38% percent Silver Diamine Fluoride (SDF; also known as Diammine Silver Fluoride- DSF) is a potentially safe, non-invasive, quick, simple to use agent that will be tested in this trial for its ability to arrest cavitated lesions with exposed dentin in primary teeth (Chu et al., 2002; Llodra et al., 2005; Tan et al., 2010; Liu et al., 2012). SDF has been used since the 1970s in Asia as a caries arresting and anti-hypersensitivity agent, and in 2014 SDF was cleared by the Food and Drug Administration (FDA) as a medical device to manage hypersensitive teeth in adults.

This trial is a Phase III, multisite, randomized, placebo-controlled superiority trial, with two parallel groups: SDF/placebo, applied at baseline and at approximately 6 months. The primary hypothesis of the trial is that 38% SDF is superior to placebo for arresting cavitated caries lesions with dentin exposed in primary teeth when assessed approximately 6 months after initial application.

Objectives:

Primary:

- Aim 1: Assess the efficacy of 38% SDF applied once, to arrest cavitated lesions exposing dentin clinically, in the primary dentition at 6 month follow-up.

Secondary:

- Aim 2: Assess the efficacy of 38% SDF applied twice, approximately 6 months apart, to arrest cavitated lesions exposing dentin clinically, in the primary dentition at 8 month follow-up.
- Sub Aim 2a: Compare the effect of one application of 38% SDF at 3 and 6 month follow-ups.
- Aim 3: Assess the impact of 38% SDF applied twice, approximately 6 months apart, on pain in children.
- Sub Aim 3a: Assess the impact of 38% SDF on pain after a single application (assessed at 3 and 6 months).

Tertiary:

- Aim 4: Assess the impact of 38% SDF on Family-Level Outcomes:
- Sub Aim 4a: Assess the impact of 38% SDF on Oral Health-Related Quality of Life (OHRQoL).
- Sub Aim 4b: Assess the effect of 38% SDF on Treatment Satisfaction and Acceptability.

Population: 1144 generally healthy children 12-71 months of age, with S-ECC, and at least one cavitated caries lesion exposing dentin clinically (International Caries Detection and Assessment System- ICDAS scores 5 or 6) at the baseline caries examination. Participants may undergo a pre-screening examination as part of the annual dental examination for school-based early childhood education programs such as Head Start, Early Head Start, and equivalent city/state subsidized preschool programs in Michigan, Iowa, or New York, or in clinics associated with the three clinical sites. Children of all demographics and genders will be included.

Phase: III

Clinical Sites: University of Michigan School of Dentistry
New York University College of Dentistry
University of Iowa College of Dentistry

Description of Intervention: Advantage Arrest[TM] (Elevate Oral Care, LLC., West Palm Beach, FL) is the 38% SDF drug brand to be used in this trial. The trial drug, 38% Silver Diamine Fluoride [$Ag(NH_3)_2F$], a clear and blue-tinted solution, will be applied to each eligible carious lesion with a disposable applicator at baseline and approximately at 6 months. For participants randomized to the placebo group, non-fluoridated deionized water containing a blue tint will be applied to each carious lesion, in the same manner as the SDF is applied.

Trial Duration: 5 years

Participant Participation Duration: Approximately 8 months (from baseline exam to final exam)

Estimated Time to Complete Enrollment: Approximately 4 years

Schematic of Trial Design:

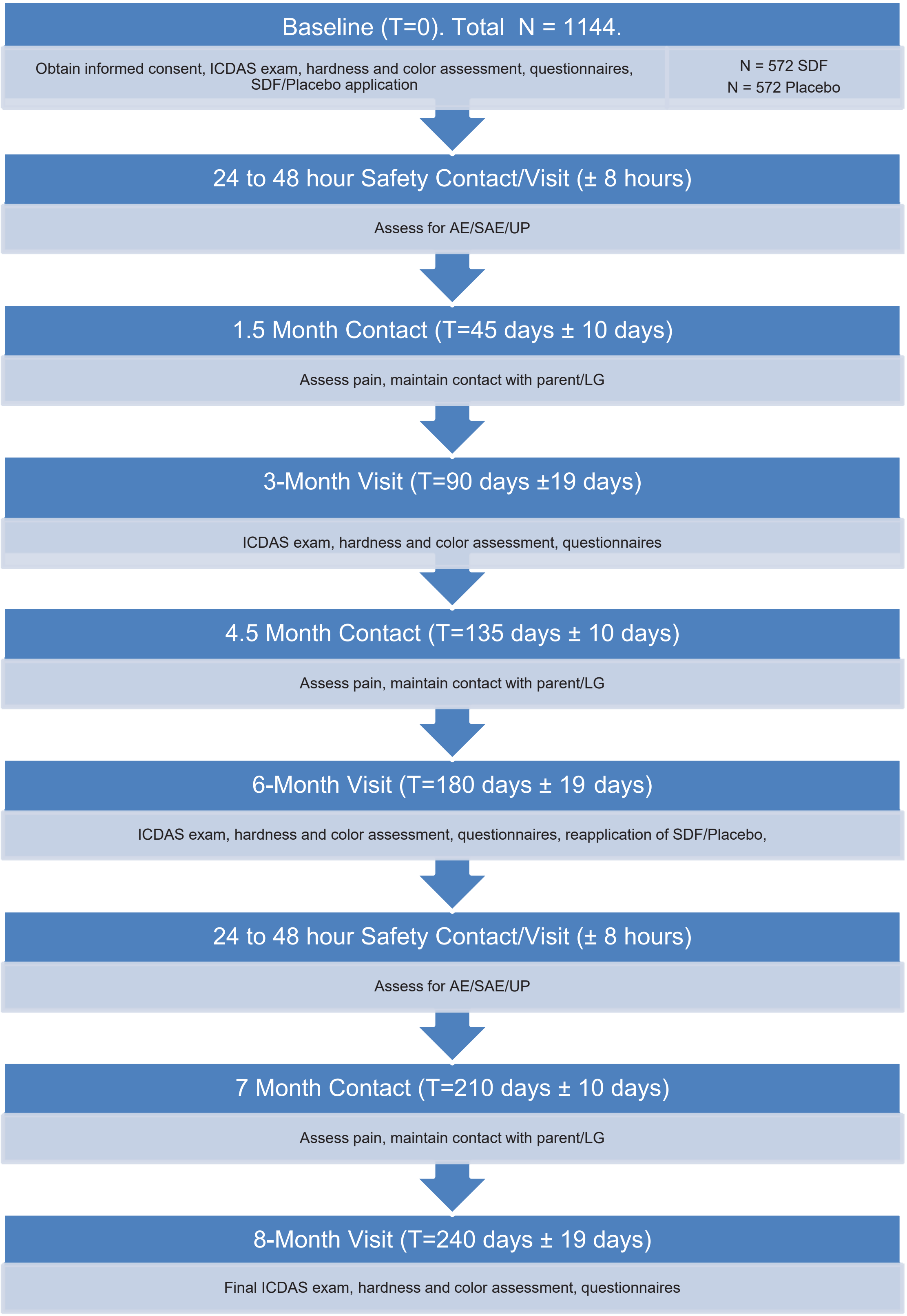

1 KEY ROLES AND CONTACT INFORMATION

Principal Investigator:

Margherita Fontana, DDS, PhD
Professor, Dept. Cariology, Restorative Sciences & Endodontics
University of Michigan School of Dentistry
1011 N. University, Room 2393, Ann Arbor, MI 48109-1078, USA
Phone: 734-647-1225
Fax: 734-936-1597
Email: mfontan@umich.edu

NIDCR Medical Monitor:

Kevin McBryde, MD
Senior Medical Officer
NIH/NIDCR/DER
6701 Democracy Blvd., Room 638
Bethesda, MD 20892-4878
Phone: 301-594-0170
Email: mcbrydekd@nidcr.nih.gov

NIH Project Scientist:

Jane Atkinson, DDS
Director, Trial Innovation Network
National Center for Advancing Translational Sciences
6701 Democracy Blvd., Room 922
Bethesda, Maryland 20892
Phone: 301-827-6031
Email: jatkinso@mail.nih.gov

NIDCR Program Official:

Dena Fischer, DDS, MSD, MS
Acting, Director, Center for Clinical Research
National Institute of Dental and Craniofacial Research
6701 Democracy Blvd., MSC 4878
Bethesda, MD 20892-4878
Phone: 301-594-4876
Email: dena.fischer@nih.gov

Clinical Site Investigators:

Carlos Gonzalez-Cabezas, DDS, MSD, PhD
Associate Professor, Dept. Cariology, Restorative Sciences & Endodontics
University of Michigan School of Dentistry
1011 N. University, Room 2395, Ann Arbor, MI 48109-1078, USA
Phone: 734-763-3391
Fax: 734-936-1597
Email: carlosgc@umich.edu

Amr Moursi, DDS, PhD
Chair, Department of Pediatric Density
New York University College of Dentistry
421 First Avenue, Room 969 W Weissman
New York, NY 10010
Phone: 212-995-9435
Fax: 212-995-4364
Email: moursi@nyu.edu

Steve Levy, DDS, MPH
Professor, Department of Preventive & Community Dentistry
University of Iowa College of Dentistry
801 Newton Road
Iowa City, IA 52242
Phone: 319-335-7185
Fax: 319-335-7389
Email: steven-levy@uiowa.edu

Data & Clinical Coordinating Center (DCCC):

Emily Yanca
Project Manager
University of Michigan School of Dentistry
1011 N. University, Room 2331, Ann Arbor, MI 48109-1078, USA
Phone: 734-763-3988
Fax: 734-936-1597
Email: emthorpe@umich.edu

Other Key Personnel:

George Eckert, MAS
Biostatistician
Indiana University School of Medicine
410 W. Tenth St., Suite 3000
Indianapolis, IN 46202-3012
Phone: 317-274-2884
Fax: 317-274-2678
Email: geckert@iu.edu

Barry Katz, PhD, MPH
Professor and Chair, Department of Biostatistics
Indiana University School of Medicine
410 W. Tenth St., Suite 3000
Indianapolis, IN 46202-3012
Phone: 317-274-2661
Fax: 317-274-2678
Email: bkatz@iu.edu

2 INTRODUCTION: BACKGROUND INFORMATION AND SCIENTIFIC RATIONALE

2.1 Background Information

Early Childhood Caries (ECC) is defined as the presence of one or more decayed, missing or filled primary tooth surfaces (dmfs) in a child 71 months of age or younger (Drury et al., 1999). The term severe-ECC refers to "progressive" or "rampant" patterns of dental caries (American Dental Association-ADA Definition of ECC, 2000). According to the American Academy of Pediatric Dentistry (AAPD Definition of ECC, 2008), in children younger than age 3, any sign (white spot or cavitation) of smooth-surface caries is indicative of severe-ECC. From ages 3 through 5, ≥1 dmfs in maxillary anterior teeth; or a dmfs score of ≥4 (age 3), ≥5 (age 4), or ≥6 (age 5) constitutes severe-ECC.

Dental caries is one of the most prevalent chronic diseases among children in the U.S. and the world (Kassebaum et al., 2015), and one of the most common unmet healthcare needs of poor children. ECC has long-term broad dental, medical, societal, economic and oral health-related quality of life (OHRQoL; Locker and Allen, 2007) consequences (e.g., young children with ECC have poor OHRQoL; Malden et al., 2008; Turton et al., 2013).

There are profound disparities in oral health and access to dental care. In the US, as much as 80% of caries incidence is experienced by only 20-25% of children, with 10% having untreated cavities, and those from low socioeconomic and minority groups experiencing significantly higher rates and at younger ages (Dye et al., 2007 and 2015). There also are clear issues related to access to care. The lack of uniformity in caries distribution underscores issues related to resource allocation. Limited data indicate that although roughly 60% of dentists accept Medicaid-enrolled patients, rates of treatment for Medicaid enrollees is much lower when more stringent criteria for Medicaid participation are utilized (e.g., approximately 20% of Medicaid-enrolled dentists treat at least 100 patients in 1 year) (Warder and Edelstein, 2017). Similarly, an American Dental Association (ADA) survey indicated that most general dentists (~96%) do not see children until 6 years of age (ADA, 2012), leaving care for children under 6 to the very limited pediatric dental workforce.

The current standard of care for management of caries once cavitated lesions develop is mostly limited in the U.S. to restoring these lesions. Especially in the case of young children who need extensive treatment, or are uncooperative and/or have immature cognitive functioning, disabilities, or medical conditions, treatment is often provided in hospital operating rooms with the child under general anesthesia (AAPD Policy on the Use of Deep Sedation and General Anesthesia in the Pediatric Dental Office, 2012), with all associated health, economic and frequent relapse issues (Berkowitz, 2003). In 2016, the FDA issued a notice to health care providers that general anesthesia in young children can result in permanent neurological damage and should be avoided. To address this concern, there is an ongoing randomized clinical trial (RCT) in the United Kingdom (i.e., the FiCTION trial; Innes et al., 2013) to assess effective management strategies for cavities in primary teeth, with a “preventive” arm in which children are exposed to preventive recommendations and treatments without receiving any restorative intervention for cavitated lesions.

Fluoride Varnish (FV) is one of the most simple, easy and possibly effective strategies currently available to prevent ECC or treat non-cavitated caries lesions (i.e., white spot lesions; Marinho et al., 2013); however, it is not effective in treating cavitated lesions. When tested in an RCT, FV had the same effect as providing no treatment in management of cavitated lesions (Chu et al., 2002). Also, because FVs are approved by the FDA in the U.S. as “devices” for reducing hypersensitivity, not “drugs” for preventing dental caries, there is no oversight to ensure products are effective and safe for caries control. This has resulted in great product variability in terms of in vitro and in vivo Fluoride (F) release and in vitro

remineralization. This raises concerns about clinical anti-caries efficacy of some existing products (Lippert et al., 2013, Gonzalez-Cabezas and Flannagan, 2014 and 2015).

The previously described factors resulted in a significant interest in developing and/or evaluating non-invasive, safe, simple, and effective strategies for the management of cavitated caries lesions (i.e., holes in the teeth with dentin exposed) in young children that are acceptable, inexpensive (and thus cost-effective), and feasible to implement in a variety of settings (dental and non-dental). Such alternative strategies to restorative care should increase access to care, help reduce disparities in young children, and diminish the excess morbidity and mortality associated with the disease.

The product investigated in this trial is 38% silver diamine fluoride (SDF; also known as Diammine Silver Fluoride- DSF). SDF is currently marketed in the United States as Advantage Arrest™, an FDA approved device to manage dentin hypersensitivity in adults. The effectiveness of SDF will be compared to a placebo for arrest of cavitated lesions with dentin exposed in primary teeth, an off-label use of the current FDA approval for SDF.

2.2 Rationale

The overarching goal of this trial is to determine if SDF is an effective and acceptable strategy for the management of cavitated lesions with dentin exposed in primary teeth.

Children experiencing S-ECC will be enrolled in this trial. To recruit children with cavitated lesions with dentin exposed (ICDAS scores 5 or 6), young children enrolled in school-based early childhood education programs such as Head Start, Early Head Start and equivalent city/state-subsidized preschool programs, or children attending clinics associated with the clinical trial sites, will be screened for untreated cavitated lesions with dentin exposed. These programs typically have higher untreated cavitated lesion rates than the national average.

The trial product, 38% SDF or placebo will be applied twice, approximately 6 months apart.

The use of 38% SDF for caries management is drawing increasing attention throughout the world. Recent systematic reviews and meta-analyses of existing RCT or quasi randomized clinical trials have concluded that 38% SDF is a potentially safe, non-invasive, quick, simple to use, and potentially effective agent, with reported effect sizes for percentages of primary teeth with arrested cavitated caries lesions from 66% (Gao et al., 2016a; Mei et al., 2016) to 81% (Gao et al., 2016b). SDF has been used since the 1970s in Asia as a cavity-arresting and anti-hypersensitivity agent. The literature reports a very high percentage of caries lesion arrest after a single application of SDF (~79%; Zhi et al., 2012), with increases after repeated application after 6 months (~91%; Zhi et al., 2012). This is the reason for assessing the effect of SDF after a single application, and also after being applied twice, approximately 6 months apart. As children will be clinically examined at baseline and 3, 6 and 8 month visits in this trial, and SDF applied at baseline and approximately 6 months post-baseline, we will be able to evaluate the effect after 1 and 2 applications. Regarding application method, no caries removal is necessary prior to application (Chu et al., 2002). Lesions are first cleaned of food debris and gross visible plaque, dried, and then the solution is painted on the dentin of each targeted cavitated lesion using a standardized applicator. One drop (approximately 100 μl) may treat many lesions, depending on their size. We will use up to 2 drops per child per visit in this trial. The arrest happens very quickly after application: 72% (95% CI: 55-84%) of cavities turned hard within 2 weeks when treated once with SDF vs. 5% (95% CI: 0-16%) when treated with a placebo (i.e., water) in a recent short-term RCT conducted in Head Start programs (Milgrom et al., 2018). Limited data suggests there might be a difference in efficacy based on lesion location and size (Zhi et al., 2012), thus we will use these variables as covariates in our analyses.

In gaining clearance by the FDA, Advantage Silver Dental Arrest, LLC conducted female and male rat and mouse studies to determine the lethal dose (LD50) of SDF by oral and subcutaneous administration. The oral LD50 was between 520 and 570 mg/kg. The subcutaneous LD50 was between 442 and 440 mg/kg. The relative safety margin of using 2 entire drops on a 10 kg child is: 440 mg/kg subcutaneous LD50 /8.6 mg/kg dose = 51-fold safety margin (see Section 6.3). In Japan, the product is used for treatment of dental caries in adults and children, as well as for sensitivity. The Japanese manufacturer of the identical product estimates that more than 2 million multi-use containers of Saforide have been sold since it was approved by Japanese authorities, including between 41,000 and 48,000 units each year in the most recent three reporting years. In the three-year reporting period, there was not a single adverse event reported. Furthermore, the lack of adverse events reported after SDF use in Japan suggests that SDF will be well tolerated and will not result in significant toxicity. Recent studies have found that >90% of silver compounds applied to tooth samples are incorporated into the tooth substance and contribute to increased mineral density (Besinis et al, 2013; Zhi et al, 2013). This suggests that systemic exposure to the silver compound following SDF application may be limited.

Advantage Silver Dental Arrest, LLC conducted a clinical efficacy and safety study in 2 clinical sites in Peru, after consultation with FDA staff regarding the design (Castillo et al. 2011). Exposure to SDF in adults produced no difference in mean Gingival Index (GI) score between test (n=63) and placebo (n=63) groups at 24 hours or seven days. Erythema scores above grade 1 were transiently higher for the test group at 24 hours at one site and not the other, while neither site's participants showed a difference between test and placebo at seven days. An independent examiner masked to the trial intervention, using photographs, confirmed the finding that no white or dark changes or instances of ulceration were visible in any participant at any time point at either clinical trial site. Similarly, there was no evidence of systemic reaction. No participant reported nausea, vomiting, gastrointestinal upset or other potential acute reaction to the product. The finding of no or only transient changes to the gingival tissues is consistent with other studies, as described next.

In four randomized clinical trials in which multiple teeth were treated with SDF for dental caries in preschool children, school-age children, and elders, not a single unexpected adverse event was reported (Llodra et al. 2005; Chu et al., 2002; Tan et al., 2010; Liu et al., 2012). In the Llodra et al. study (2005), 3 out of 225 children treated with SDF had transient mildly painful white lesions on the gingival surface near the tooth treated. These lesions resolved without treatment in 48 hours when the children were re-examined. These transient effects, although mildly adverse, were not unexpected. In a recent study in the US, targeting 3-5 year old children in Head Start centers, 38% SDF or placebo was applied (Milgrom et al., 2018). Within 24 to 48 hours of treatment, trained staff contacted parent/caregivers by telephone about adverse events. The questions included: (1) Has your child required medical care since his/her dental visit in the last 48 hours? (2) Has your child been to an emergency room, medical doctor, nurse or health care provider? (3) Since receiving the solution on the teeth has your child experienced any of the following? Nausea, not eating, vomiting, difficulty swallowing or breathing, swelling around the lips or skin of the face, itchiness around the lips or skin of the face, hives or rash, stomach ache, or diarrhea. Eight adverse events were reported by parents, 4 in each group. Based on enrolled participants, the adverse event rate was 11.1% in the placebo group (exact 95% CI: 3.2 to 26.7%) and 13.3% in the 38% SDF group (exact 95% CI: 3.7 to 30.7%). Based on all contacted participants, the adverse event rate was 15.4% in the placebo group (exact 95% CI: 4.3 to 34.9%) and 16.0% in the 38% SDF group (exact 95% CI: 4.5 to 36.1%). There were no statistically significant differences by treatment. The type of adverse event, severity and relationship of the adverse event to the treatment condition are reported in the Tables 1 and 2 below. The majority of the adverse events were either diarrhea or stomach ache, mild in severity and all resolved within 2 days of reporting. At the follow-up visit 14 to 21 days after treatment, a dental provider performed a visual examination to detect gingival or soft tissue stomatitis or ulcerative lesions. No participant was identified at that point with gingival or soft tissue stomatitis or ulcerative lesions.

Table 1. Completion of 24-48 hour follow-up and reported adverse events in Milgrom et al., 2018 study in 3-5 year old children after application of 38% SDF or placebo

	All participants N = 66	Placebo N = 35	SDF N = 30
24-48 hour follow-up, n (%)			
Completed	51 (77.3)	26 (62.2)	25 (83.3)
Unable to contact	15 (22.7)	10 (27.8)	5 (16.7)
Adverse event, n (%)	8 (12.1)	4 (11.1)	4 (13.3)

Table 2. Harms in Milgrom et al., 2018 study in 3-5 year old children

Study identification	Group	Adverse event description	Severity of event	Relationship of event to study product
1015	Placebo	Diarrhea	Mild	Unrelated
2004	Placebo	Diarrhea	Mild	Possibly related
2082	Placebo	Stomach ache	Moderate	Possibly related
3052	Placebo	Sporadic sore & hurting tooth; Diarrhea	Mild	Probably not related
1003	SDF	Flu like symptoms (nausea, vomiting, stomach ache, diarrhea)	Mild	Probably not related
1007	SDF	Redness around mouth, but not sore or irritated	Mild	Probably not related
1008	SDF	Spot on corner of lip; looked like a burn but was flat, not sore or irritated	Mild	Possibly related
3056	SDF	Nausea and stomach ache	Mild	Probably not related

2.3 Potential Risks and Benefits

2.3.1 Potential Risks

Potential SDF-affiliated risks to human subjects include: discoloration of cavitated lesions, possibility that treated lesions will progress between enrollment and the 8-month final trial visit if the SDF does not arrest the lesion, transient metallic or bitter taste, and temporary irritation of soft tissues (e.g., erythema, ulceration) after application. Similar risks apply to the placebo group, as lesions may discolor and progress between enrollment and the 8-month final trial visit. If SDF contacts the skin there may be a temporary tattoo that will resolve within 2 weeks. Potential safety risks associated with an allergic reaction or gastrointestinal disturbances will be assessed during the trial, to include: nausea, not eating, vomiting, difficulty swallowing or breathing, swelling around the lips or skin of the face, itchiness around the lips or skin of the face, hives or rash, stomach ache, or diarrhea. Other risks associated with trial procedures include potential trauma as a result of the ICDAS examination, (this risk is no greater than the risk from a traditional dental examination); cross-contamination associated with the ICDAS and intraoral photographs; and potential loss of confidentiality. Additionally, the trial questionnaires pose minimal risk of discomfort as they do not contain any disturbing material.

The protocol has been designed to minimize these risks. Particularly, as there is risk of disease progression while the child is in this trial, all children will be followed up closely, with the trial team facilitating access to care immediately upon trial completion, or whenever necessary throughout the trial. Children enrolled in the trial will not be denied services that constitute the current clinical standard of care, except that they will be asked to not brush with fluoridated toothpaste the day of the SDF/placebo application and to not receive fluoride varnish application that day. Furthermore, all parents/guardians will be encouraged to have their children receive all preventive services, including fluoride varnish applications, that would typically be provided to children in the program or setting from which they are recruited. Parents/caregivers of all child study participants will be advised to seek follow-up care for their children at the end of the study. During the 1.5-, 4.5-, and 7-Month Intermediate Contacts, which will take place between the frequent trial visits (3, 6, and 8 months), the parent will be asked questions to help determine if the child has toothache pain and disease progression. Additionally, parents/legal guardians will be instructed to contact the trial team at any point during the trial if they have any questions or concerns. See Section 9 for a description of safety procedures. See Manual of Procedures for more details.

To decrease potential risks associated with SDF usage, teeth will be dried with cotton or gauze prior to application, and excess fluid will be blot dried after application. Follow-up of participants will take place approximately 24-48 hours after study product is applied, to record any adverse events that occur following SDF/placebo administration. Safety monitoring is described in detail in Section 9.

Furthermore, dental examinations and other data collection procedures will be performed by experienced personnel well versed in proper trial methodology and Good Clinical Practices. Risk of infection and cross-contamination will be minimized by following established infection control procedures developed by the University of Michigan, University of Iowa, and New York University, which are in compliance with all state and federal mandates. In addition, training will be provided to ensure the appropriate management of young children (although all trial sites have extensive experience working with young children in clinical research), and every attempt will be made to maintain examination times as short as possible.

The primary caregiver could be uncomfortable answering some of the questions in the trial questionnaires. The primary caregiver may choose to decline responding to any question(s) s/he is not comfortable answering. Precautions will be in place to minimize the risk of loss of confidentiality, such as collecting only minimal identifying information and storing data collection documents in locked cabinets (located in locked offices), using unique trial codes for participants, using encrypted computers, and maintaining electronic data files on a password-protected computer drive. Compliance with all IRB regulations concerning data collection, data analysis, data storage, and data destruction will be strictly observed.

2.3.2 Potential Benefits

During the randomized clinical trial, benefits to the participants include periodic dental examinations, with results of all dental examinations provided to the parent or legal guardian. Additionally, for those participants in the intervention arm, the intervention might arrest caries lesion progression, decrease pain, and improve quality of life for the child and family. In the case of serious dental health problems (e.g., abscesses, pain, etc.), parents/legal guardians and children will be offered an unscheduled trial visit and referred for care for these issues by the trial team. Please see the Manual of Procedures (MOP) for more details.

Additionally, the trial team will work with parents/legal guardians so appointments are in place for an immediate non-trial dental visit upon completion. Up to $250 will be reimbursed, if needed, for emergency care costs associated with a trial tooth within 6 months after withdrawal from the trial (during

a 6- to 9-month hospital operating room waiting period in the teams' clinics, 8-12% of patients required emergency visits). Furthermore, at baseline (and at any other point when teeth requiring treatment are identified), the parent/legal guardian will be informed of any ineligible teeth requiring treatment, and the trial team will facilitate referrals for care for these teeth.

If efficacy is demonstrated for SDF, the anticipated long-term benefit for future non-trial children is increased access to care by providing a cost-effective, non-invasive option as an alternative for managing cavitated caries lesions in young children. The anticipated benefit for society is having an indication for caries arrest for SDF use, if effective, which would ensure regulation of efficacy and safety over time.

3 OBJECTIVES

3.1 Trial Objectives

Aim 1: The primary aim of this trial is to assess the efficacy of 38% SDF applied once, to arrest cavitated lesions (caries lesions with cavities that expose dentin clinically, but with no pulp exposure, pain, mobility, or clinical signs of pulpal infection) in the primary dentition (assessed using the International Caries Detection and Assessment System-ICDAS II activity criteria, Appendix A) at 6 month follow-up.

Aim 2: The secondary aim (not intended for drug labeling claims) is to assess the efficacy of 38% SDF applied twice, approximately 6 months apart, to arrest cavitated lesions in the primary dentition at approximately 8 months after initial application. A comparison of the effect of one application will be assessed over time at 3 and 6 months follow-ups (Sub-Aim 2a).

Aim 3: The next secondary aim (not intended for drug labeling claims) is to assess the impact of 38% SDF applied twice, approximately 6 months apart on pain in children, through use of the Dental Discomfort Questionnaire. The impact of 38% SDF on pain after a single application will be assessed at approximately 3 and 6 months (Sub-Aim 3a).

Aim 4: The tertiary aim (not intended for drug labeling claims) is to assess the effect of 38% SDF applied once (assessed at approximately 6 months after initial application) and twice, approximately 6 months apart (assessed at approximately 8 months after initial application) on family-level outcomes, assessing the impact on oral health-related quality of life (Sub-Aim 4a), and on treatment satisfaction and acceptability (e.g., associated with the change in color of treated lesions, etc.; Sub-Aim 4b). The relationship between treatment satisfaction/acceptability and other factors (e.g., family experience with ECC, oral hygiene and dietary habits) will be evaluated.

3.2 Trial Outcome Measures

3.2.1 Primary

Efficacy of 38% SDF to arrest Cavitated Caries Lesions (Aim 1): Lesion arrest will be assessed for all cavitated lesions in the mouth (whether included or not in the trial, to reduce bias) using the ICDAS II system activity criteria, which categorizes lesion activity for ICDAS scores 5-6 based on dentin hardness [i.e., arrested lesions will change over time from soft-included in the trial at baseline- to hard, without an increase in ICDAS severity score (see Appendix A)]. The primary outcome used in the analyses will be the proportion of arrested trial lesions per child in each treatment arm. For the purposes of testing the hypothesis, the primary outcome will be measured at about 6 months post initial treatment (Aim 1).

3.2.2 Secondary

The following secondary outcomes associated with application of 38% SDF will be assessed, but are not intended to support drug labeling claims:

- Efficacy of 38% SDF to arrest Cavitated Caries Lesions when applied twice, approximately 6 months apart: lesion arrest will be measured at approximately 8 months post initial treatment to assess efficacy (Aim 2). Compare (Sub-Aim 2a) the effect of one application of SDF measured at approximately 3 and 6 months post initial treatment.

- Child's Pain (Aim 3): The pain outcome will be defined as the proportion of children in each treatment arm experiencing toothache pain. Additional pain outcomes will be the number of episodes of pain associated with treated teeth for each child in each arm during the follow-up period and the average recorded pain score for children in each treatment arm. The presence of pain associated with a trial tooth will be defined as a score of 1 or higher using the Dental Discomfort Questionnaire (DDQ; Appendix B and Section 8), which a trial examiner can associate with a trial tooth during a clinical visit. The number of pain episodes is the total of all

in-person visits at which a DDQ score of 1 or higher is confirmed by the trial examiner to be associated with a trial tooth. The pain score will be calculated using the DDQ.

3.2.3 Tertiary

The following tertiary outcomes associated with the effect of 38% SDF on the Family will be assessed, but are not intended to support drug labeling claims:

- Oral Health-Related Quality of Life (OHRQoL): OHRQoL will be assessed at baseline, 3-, 6-, and 8-month visits. Parents/legal guardians will be asked to complete the 16-item Parental-Caregiver Perception Questionnaire (P-CPQ) and 8-item Family Impact Scale (FIS) questionnaire (Thomson et al., 2013; Appendix C). The P-CPQ and FIS total and subscale scores are computed by summing the scores.

- Treatment Satisfaction and Acceptability: This will be assessed at baseline/24-48 hour contact/visit, and 3-, 6-, and 8-month visits using the questionnaire labeled as Family Survey (Appendix D). Parents/legal guardians in each treatment arm will be asked about their level of satisfaction with the appearance of their children’s teeth, and their overall level of acceptance and satisfaction with the treatment received. They will also be asked about their family experiences with ECC, as well as the child’s brushing and dietary habits, and these items will be correlated with acceptability and satisfaction (e.g., adults having prior experience with the consequences of ECC and associated stress might be more accepting of this treatment alternative). In addition, color of treated lesions will be determined (yellow, brown, black), not as a primary indicator of lesion activity, but the change in color over time will be used for both trial arms as a measure of consequences associated with treatment assessed in the satisfaction questionnaire.

4 TRIAL DESIGN

This trial is a phase III, multicenter, randomized, placebo-controlled superiority trial, with two parallel groups (placebo vs. 38% SDF applied to cavitated lesions) involving a total of 1144 children with the primary outcome assessed approximately 6 months after initial treatment.

One thousand one hundred forty-four children, 12-71 months of age, from early childhood education programs, such as Head Start centers, Early Head Start centers, and equivalent city/state subsidized preschool programs, or children attending clinics associated with the clinical trial sites, will be randomized. All children will be followed for approximately 8 months. Trial enrollment will occur over a course of approximately 4 years. Trial visits will occur at baseline, and approximately 3 months, 6 months, and 8 months. SDF/placebo will be applied at baseline and 6 month visits. Parents/legal guardians will be contacted approximately 24 to 48 hours after the SDF/placebo application to assess adverse events and unanticipated problems and in-person visits for safety exams will be available for all child participants approximately 24 to 48 hours after SDF/placebo application. This visit could happen before or after the 24- to 48-hour contact with the parent or legal guardian, but every attempt will be made so that the contact occurs prior to the 24- to 48-hour visit (unless the parent/legal guardian is able to attend the 24-48 hour visit and answer questions in person). Additionally, intermediate contacts at approximately 1.5 months, 4.5 months, and 7 months will occur to determine if the child needs an additional visit to assess pain, lesion progression, AEs and UPs, etc. and to maintain contact with the participant.

Randomization to 38% SDF application (treatment) or placebo (control) will be at the participant-level; all teeth within a participant that meet the inclusion criteria will receive the same trial product. Both treatment and control will be dispensed from identical unit-dose ampules coded and labelled to ensure masking of all trial personnel. The number of ampules required to treat all carious lesions in the participant’s mouth will be recorded. No caries removal will be performed. Lesions will be cleaned with a toothbrush or microbrush to remove food debris and gross visible plaque, and will be dried with cotton/gauze, and the solution will be painted on the dentin of each targeted cavitated lesion using a standardized applicator. Following application, the lesion will be blotted dry with gauze if excess fluid is present.

At each clinical visit ICDAS examinations, including cavitated lesion hardness assessments, soft tissue assessments and questionnaires on dental discomfort, family impact and treatment satisfaction and acceptability will be collected.

5 TRIAL ENROLLMENT AND WITHDRAWAL

5.1 Participant Inclusion Criteria

In order to be eligible to participate in this trial, participants must meet all of the following criteria:

Child:

- Male or female, between 12-71 months of age at baseline, up to the day the child turns 6 years old.
- Must allow examination of the oral cavity and application of treatment by the examiners at baseline.
- Must have S-ECC

  [defined as: In children younger than age 3, any sign (non-cavitated or cavitated lesion) of caries in any tooth surface (i.e., most common for this age group will be on erupted smooth surfaces). From ages 3 through 5, ≥1 dmfs in maxillary anterior teeth; or a dmfs score of ≥4 (age 3), ≥5 (age 4), or ≥6 (age 5) constitutes S-ECC. Note: The "d" component of the dmfs index is defined as including cavitated and non-cavitated lesions, thus ICDAS≥1 (Appendix A)].
- Have at least one SDF-target tooth with
  - Soft cavitated caries lesions extending into dentin [ICDAS 5 or 6 (Appendix A)];
  - Cavitated lesion(s) that allow for direct hardness assessment and application of SDF (microbrush applicator must fit the cavity and be able to access all exposed dentin).

Parent/Legal Guardian:

- Provide written informed consent for the child and her/himself prior to participation.
- Must be at least 18 years old, or an emancipated minor who provides documentation of emancipation.
- Must be willing and able to participate in trial activities.

5.2 Participant Exclusion Criteria

An individual who meets any of the following criteria will be excluded from participation in this trial:

Child:

- Hereditary generalized developmental dental defects such as Amelogenesis Imperfecta and Dentinogenesis Imperfecta.
- Known allergy/sensitivity to silver or other heavy metal ions.
- Presence of any gingival or peri-oral ulceration, abscess or stomatitis.
- Participating in the foster care system at trial initiation.
- Toothache pain at baseline (based on Dental Discomfort Questionnaire score of 1 or higher (Appendix B).
  - Note: If toothache pain occurs after baseline, the child remains eligible to continue in the trial as long as he/she has at least one trial tooth that meets tooth inclusion/exclusion criteria.
- Demonstrated inability to comply with trial protocol requirements (determination is at the Clinical Site Investigator's discretion).
- Rickets.
- Osteopenia or osteoporosis (e.g., Osteogenesis Imperfecta, Ehlers-Danlos Syndrome, Marfan Syndrome, etc.).
- Chronic diseases such as chronic kidney disease, leukemia, lymphoma, rheumatic disorders, etc.
- Metabolic bone disease (e.g., Galactosemia, Glycogen Storage Disease Type 1, etc.).

- Chronic glucocorticoid, anticonvulsants, chemotherapy, bisphosphonate administration.
- Hypothyroidism, hyperparathyroidism, impaired glucose tolerance, hypocalcemia or hypophosphatemia.

Tooth:

- Pain due to caries at baseline (based on DDQ score of 1 or higher, Appendix B).
    - Note: If toothache pain occurs after baseline due to a trial tooth, the tooth is removed from the trial.
- Pulpal exposure, or signs of pulpal infection (abscess, fistula, swelling).
- Mobility not associated with expected exfoliation patterns.

Parent/Legal Guardian:

- Demonstrated inability to comply with trial protocol requirements (determination is at the Clinical Site Investigator's discretion).
- Inability to read and comprehend the consent document or trial questionnaires in the translated languages available.

5.3 Strategies for Recruitment and Retention

5.3.1 Recruitment

The New York University site will focus on providing a population that is largely Hispanic and Asian, the Iowa site will provide a population primarily White, and the Michigan site will provide a large percentage of Black and Hispanic children. It is expected that a recruitment period of 4 years should be sufficient to approach and recruit the number of participants needed. Untreated caries rates in children age 24-60 months nationwide are 10%, with 23% with some caries experience (Dye et al., 2015). Anderson et al (2010) reported 23% of children in Head Start programs in New Hampshire to have caries lesions that needed treatment (and thus were cavitated), and stated this was similar to the rest of the country. Based on preliminary experience, it is anticipated approximately half of these children with untreated caries will meet eligibility criteria for the trial. Thus, an approach: enroll ratio of 100:12 is estimated. To enroll 1,144 children, an estimated 9,534 will be approached.

Recruitment at a variety of school-based early childhood education programs, such as Head Start, Early Head Start, and city/state subsidized preschool programs, and clinics associated with the clinical sites, will help achieve a racially and ethnically diverse population.

In the case of Head Start programs, children must present proof of a dental "examination" within 90 days of enrollment (generally September through November). This "examination" collects reportable data required for early childhood education programs on presence of untreated caries lesions (i.e., cavitated lesions) and caries experience (i.e., restorations).

As this dental "examination" may occur during the school day when parents/legal guardians are not present, the trial teams will coordinate with schools so that when the "screening" results are sent home, trial information and invitation to schedule a baseline trial visit will be sent home with the child as well. Interested families can then return their contact information to the center or call the trial team. In addition, the trial team will request a waiver of consent and waiver of HIPAA from the IRB of record to use the pre-screening data to identify children with potentially-eligible carious lesions and to ask the early childhood education program to obtain their phone number to contact these families.

Once the trial team communicates with the family over the phone, they will provide a brief explanation of the trial and review trial inclusion/exclusion criteria. If the parent/guardian is interested in participating, a baseline trial visit will be scheduled.

Some early childhood education program recruitment pre-screenings may also be arranged as part of health fairs on evenings or weekends to encourage parent/guardian attendance. Parents/guardians will be informed of the findings from the pre-screening. If pre-screenings are done when parents/guardians are present, parents/guardians of children with eligible lesions will be informed about the trial. If interested, and if the trial team is able to also be present, parents/guardian can undergo the informed consent process and complete trial questionnaires, and the child will be scheduled for a baseline trial visit. If time allows, a baseline visit could be performed, following the pre-screening, on the same day, at the same visit. In these cases, the FV that is normally applied at these examinations would not be applied that day, but the early childhood education center would be encouraged to plan this at a later date.

To recruit from clinics associated with the trial sites, the trial team will utilize the waiver of consent and waiver of HIPAA from the IRB of record to review existing dental charts to identify children with potentially eligible carious lesions. Other clinical partners, such as pediatricians, may also refer potentially eligible children to the study team. The trial team will then contact these families to explain the trial and assess interest.

A screening log maintained at each site (refer to Manual of Procedures) will record all participants who are approached to participate in the trial. Participants who decline to participate will not be approached again during the same school/calendar year, and the reason they declined will be recorded on the screening log.

At regularly scheduled intervals, the approached-to-enrolled ratio will be examined along with the total number of enrolled pairs. These assessments will determine if recruitment goals are being met. If not, resources can be added and/or refocused on particular early childhood education programs or clinical sites to improve recruitment efforts.

Participants will receive remuneration of $100 for completion of each of the following in-person visits: baseline, 3-, 6-, and 8-month. For completing the surveys associated with the in-person visits at baseline, 3-, 6-, and 8-month, participants will receive $25 for each. For participation in the in-person 24- to 48-hour safety visits, participants will receive $25 each. In addition, all participants will receive $25 for speaking with the trial team during each of the 1.5-, 4.5-, and 7-month Intermediate Contacts.

	Visit	Remuneration
**In-person and surveys**	**Baseline**	**$125**
**In-person**	24-48 h post baseline	$25
Contact	1.5 Month	$25
**In-person and surveys**	**3 Month**	**$125**
Contact	4.5 Month	$25
**In-person and surveys**	**6 Month**	**$125**
In-person	24-48 h post 6 month	$25

Contact	7 Month	$25
**In-person and surveys**	**8 Month**	**$125**
	**Total**	**$625**

Finally, for ethical reasons, children will be monitored very closely during the trial, and the trial team will work with parents/legal guardians so appointments are in place for an immediate non-trial dental visit upon completion. Unscheduled trial visits will be performed to address dental emergency needs. Up to $30 will be provided for transportation/parking needed to attend those visits. Up to $250 will be provided, if needed, for emergency care costs of trial teeth.

5.3.2 Retention

Attrition will be minimized by the short length of the trial and by following all participants through one school year, or equivalent if recruited from a clinic. Also, permission will be requested from the participants to allow the early childhood education program to contact the participant's family if the trial team cannot. The trial team may also verify the participant contact information with the early childhood education program during the course of the trial. Parents/legal guardians will be asked to provide their address, email (if they have one), and up to 3 possible telephone numbers (e.g., home, work and cellular phone), as well as their preferred method of contact. In addition, they will be asked to provide names/telephone numbers, addresses and email, as well as the preferred method of contact (including text messages) for 3 individuals who know how to contact the parent/caregiver. Securing email addresses will also increase the likelihood of follow-up contact as these remain relatively constant.

Intermediate contact with the primary caregiver will occur regularly by phone, email or mail between clinic visits to help increase retention.

5.4 Treatment Assignment Procedures

Randomization will be stratified by site. Allocation to SDF/placebo will be 1:1.

5.4.1 Randomization Procedures

The randomization schedule will be generated by a biostatistician. Biostatisticians who will be involved in the analyses will remain masked to the trial randomization.

Randomization procedures are explained in more detail in the MOP and the Randomization Plan.

5.4.2 Masking Procedures

To ensure concealment of treatment allocation, the identical ampules containing test and placebo product will be assigned sequential identification numbers. The master randomization schedule will not be broken until analyses of the data, or if requested by the DSMB for meetings or due to safety concerns. All trial product will be sealed and labeled as required by the FDA [Section 6.0].

The following steps are being taken to minimize issues associated with bias and masking: 1) Product will be concealed in identical unit-dose ampules, identified by identification numbers, and with liquids of equal color. 2) The trial personnel applying the concealed product at baseline and 6 months will be separate from the trial ICDAS examiner. 3) The parent/guardian, child, and trial caries examiners and trial team involved in any data collection will remain masked regarding trial group allocation. 4) The primary trial outcome (hardness) will be measured independently of the outcome of color (to acknowledge teeth can turn dark, and still remain soft) for all cavitated caries lesions (not only those included and treated in the trial).

5.4.3 Breaking the Blind

Randomization code assignment will be broken only under circumstances where participant safety is in question. The participant will be withdrawn from the trial upon breaking of the randomization assignment.

Unmasking of individual subjects' treatment assignments should occur only when knowledge will have a direct bearing on the medical treatment or evaluation of a subject. Whenever possible, the need to unmask should be discussed with the Grant PI and the NIDCR Medical Monitor prior to unmasking. Unmasking procedures are described in more detail in the MOP.

A full account of the unmasking event will be recorded in the subject's source document and eCRF, including the date and time of the unmasking, the reason for the decision to unmask, the extent of unmasking, and the name of the individual who made the decision to unmask. The treatment assignment should not be included in either the source document or the eCRF.

In addition, as needed to meet regulatory reporting requirements, designated personnel may be unmasked to the treatment status of individual subjects. In this circumstance, and if there are no other concerns, neither the Grant PI, NIDCR Medical Monitor nor the site staff will be further unmasked to treatment status.

5.5 Participant Withdrawal

5.5.1 Reasons for Withdrawal

Any participant who chooses to withdraw from the trial may do so. Verbal desire to voluntarily withdraw will be recorded in the participant's trial file. Trial personnel may request that the participant notify in writing their request for withdrawal for documentation in the participant trial file. However, even if this written request is not received, record of the verbal withdrawal will suffice to ensure termination.

Both the caries exam and the SDF/placebo application must be completed at the baseline visit; otherwise, the participant will be withdrawn from the trial by the Clinical Site Investigator.

An investigator may terminate a trial participant's participation in the trial if any clinical adverse event (AE), serious adverse event (SAE), unanticipated problem (UP), or other medical condition or situation occurs such that continued participation in the trial would not be in the best interest of the participant.

5.5.2 Handling of Participant Withdrawals or Participant Discontinuation of Trial Intervention

Participants who notify the site either verbally or in writing of their desire to be withdrawn from the trial will be withdrawn. Data collected before the participant withdraws from the trial will not be removed from the research trial dataset and will be used in the trial analyses. The sponsor or the institution may stop participant involvement in this research trial at any time without consent for reasons including those listed in Section 5.6. Individual participants may be withdrawn if the investigator believes it is in the best interest of the participant. Any data collected and stored as part of the research trial may be destroyed without participant consent.

Participants who are withdrawn will be referred to a local dentist for follow-up care.

5.6 Premature Termination or Suspension of Trial

This trial may be suspended or prematurely terminated if there is sufficient reasonable cause. Written notification, documenting the reason for trial suspension or termination, will be provided by the

suspending or terminating party to the investigator, funding agency, the Investigational New Drug (IND) sponsor and regulatory authorities. If the trial is prematurely terminated or suspended, the Principal Investigator (PI) will promptly inform the sIRB and local IRBs and will provide the reason(s) for the termination or suspension.

Circumstances that may warrant termination include, but are not limited to:

- Determination of unexpected, significant, or unacceptable risk to participants.
- Insufficient adherence to protocol requirements.
- Data that are not sufficiently complete and/or evaluable.
- Determination of trial futility.
- Trial meets early stopping rules.

6 TRIAL INTERVENTION

6.1 Trial Product Description

Test Intervention: Advantage Arrest™ (Elevate Oral Care, LLC., West Palm Beach, FL).
Placebo: Non-fluoridated deionized water provided by the manufacturer (Elevate Oral Care, LLC., West Palm Beach, FL).

6.1.1 Formulation, Packaging, and Labeling

Thirty-eight percent silver diamine fluoride (SDF) Advantage Arrest™ and the placebo will be provided by the manufacturer (Elevate Oral Care, LLC.) in identical single use unit-dose ampules.

The trial drug is an Aqueous Diamine Silver Fluoride $[Ag(NH_3)_2F]$, CAS # 34445-07-3, 38.3% to 43.2% weight/volume in purified water. The clear blue-tinted solution contains:

CAS #	Ingredient	Percentage
7440-22-4	Silver (Ag)	24 - 27
1336-21-6	Ammonia (NH3)	7.5 - 11
16984-48-8	Fluoride (F)	5 - 6
7732-18-5	Deionized Water	<= 62.5
3844-45-9	FD&C Blue 1	<1

The placebo will be deionized water, also colored with the same blue colorant, in identical unit-dose ampules. The test and placebo ampules will be assigned codes. Each trial product or trial placebo ampule will be sealed and subsequently placed in a small plastic bag. Labeling will comply with the regulations for labeling of an investigational product (21 CFR Part 312.6). A certificate of analysis from the manufacturer will be obtained for each batch provided for use in the trial to confirm F and Ag concentrations of the test stock and the placebo. In addition, a certificate of analysis from the manufacturer will be obtained for Ag and F content of randomly selected and labeled ampules containing the drug and placebo, prior to clinical use. At the conclusion of the trial, the remaining stock of active drug will again be subjected by the manufacturer to confirmatory chemical analysis.

The placebo will NOT have any of the caries active ingredients: Fluoride or silver compounds.

6.1.2 Product Storage and Stability

The trial product and placebo will be stored in a dark, cool (4^0C-40^0C), dry place away from acids, alkaline materials, heat, and bleach. The containers will be stored in a locked, controlled, secure environment. Shelf life has been established by the manufacturer as 3 years. Access to the trial product and placebo will be limited to IRB-approved trial personnel only. Drug accountability (Section 6.4*)* will be closely monitored at each trial site.

6.2 Dosage, Preparation and Administration of Trial Product

Teeth that meet the inclusion criteria will be assigned to SDF application (treatment) or placebo (control), following the manufacturer's recommendations. No caries removal will be performed. Lesions will be cleaned with a toothbrush or microbrush to remove food debris and gross visible plaque, and dried with cotton/gauze, and the solution will be painted on the dentin of each targeted cavitated lesion using a standardized applicator. The amount used will be recorded as multiple ampules may be required to treat multiple carious lesions (up to 2 ampules-each containing approximately 100 µL, will be used per child, per application visit).

Universal precautions will be followed to protect participants and clinicians from exposure to potentially infectious fluids and materials.

6.3 Modification of Trial Product Administration for a Participant

No modifications of trial product administration are anticipated as the lethal dose (LD50) of SDF subcutaneously ranges between 440 and 442 mg/kg. Each ampule contains a drop. One drop (approximately 100 μL) is ample material to treat several teeth, but for a child with S-ECC, 2 drops may be needed depending on the number of teeth to be treated. One drop of Advantage Arrest™ (38.3% to 43.2% SDF) contains 38-43.2 mg SDF. Two drops would contain 76-86.4 mg SDF. Assuming children in this trial (1-5 years of age) weigh 10-18 kg (50th percentile based on the Centers for Disease Control and Prevention growth curves), for a 10 kg child, and an SDF dose for 2 drops (maximum amount used per child per application visit) of 76-86.4 mg, the dose would be 7.6-8.6 mg/kg. Thus, the relative safety margin of using 2 entire drops on a 10 kg child is: 440 mg/kg subcutaneous LD50 /8.6 mg/kg dose = 51-fold safety margin. As this trial will apply SDF at 2 separate visits, the total possible dose during the trial is doubled compared to the previous calculations, and the safety margin is 25.5-fold.

6.4 Accountability Procedures for the Trial Product

Trial product and placebo will be shipped directly from the manufacturer to each of the 3 clinical trial sites. The product should be stored in a dark, cool (4^0C-40^0C), dry place away from acids, alkaline materials, heat, and bleach. There will be a manifest each time product is shipped, and a receipt/tracking record maintained at each clinical trial site.

Investigational product accountability logs, including stock records and participant records, are to be utilized at each trial site for trial product accountability. Detailed instructions for product accountability will be contained within the MOP.

6.5 Concomitant Medications/Treatments

The date of last FV treatment will be recorded at baseline, 1.5-, 3-, 4.5-, 6-, 7- and 8-month visits. FV will not be applied the same day as SDF; time between FV and SDF applications must be at least 12 hours.

Concomitant medications associated with any adverse events which occur will be recorded.

7 TRIAL SCHEDULE

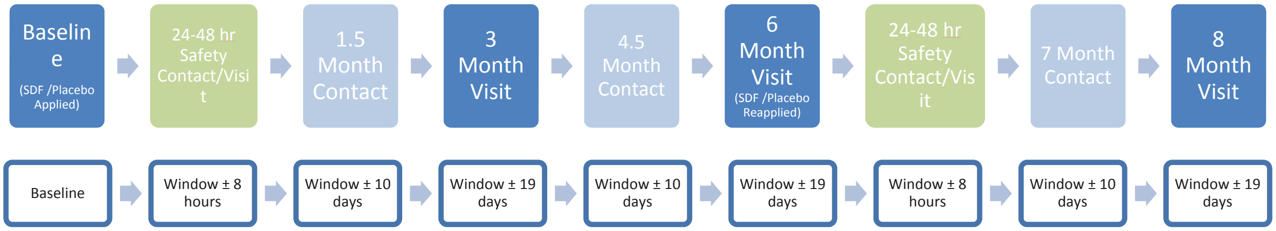

	Screening/Baseline	24-48 Hour Contact	24-48 Hour Visit	1.5 Month Contact	3 Month Visit	4.5 Month Contact	6 Month Visit	24-48 Hour Contact	24-48 Hour Visit	7 Month Contact	8 Month Visit	Unscheduled Contact	Unscheduled Visit	Withdrawal Visit
Informed Consent	X													
Medical History	X					X	X							
Oral Soft Tissue Exam	X		X		X		X		X		X		X	X
ICDAS Exam	X				X		X				X			X
SDF/Placebo Application	X						X							
Photographs of Teeth	X		X		X		X		X		X		X	X
Oral Hygiene/Diet Education	X				X		X				X			X
DDQ (Pain)	X	X		X	X	X	X	X		X	X	X	X	X
OHRQoL	X				X		X				X			X
Family Survey	X	X			X		X				X			X
Adverse Events	X	X	X	X	X	X	X	X	X	X	X	X	X	X
FV History Check	X			X	X	X	X			X	X			X

7.1 Consenting Process

Prior to the collection of any trial data, consent will be obtained by trained trial personnel. Parents or legal guardians will review the consent document with trained trial personnel. Parents or legal guardians will be allowed time to review the consent document and all questions will be answered before the parent or legal guardian consents to participate. Additionally, parents and guardians will be instructed that participation in this or any research trial is totally voluntary and deciding not to participate will not affect their ability to receive other examinations or future treatment through oral health clinics. The parent/legal guardian will be informed that they are consenting for their participation in the trial, as well as that of their child. Consent will be documented using IRB-approved and stamped consent forms. The consent process will be documented on a source document which ensures the steps of the consenting procedure have been followed.

Consent forms will be translated by a translator into selected languages. In addition, trial personnel fluent in selected languages will be available at sites if needed.

7.2 Screening/Enrollment/Baseline

Prior to the initiation of any trial procedures, the Site Coordinator will confirm written informed consent has been obtained from the parent/legal guardian, and confirm that the adult/child pair meets the eligibility criteria (except for tooth inclusion, which requires the ICDAS exam to be completed).

The parent/guardian will be asked to complete a brief medical history for the child, and the trial questionnaires. This could occur on a day other than the ICDAS examination, but must occur after informed consent is obtained.

The child will be examined by a calibrated trial examiner, who will be masked to treatment allocation and responses on the trial questionnaires. The examiner will review the medical history and the inclusion/exclusion criteria. After doing a soft tissue exam, teeth will be cleaned with a toothbrush to remove food debris and gross plaque, and a full mouth exam will be done using a clinical examination headlight, mirror, and cotton/gauze. The ICDAS examination will be conducted scoring the teeth for lesion severity and hardness/activity (Appendix A). Randomization will occur after the baseline ICDAS exam is completed and it is verified the child has eligible trial teeth. The parent/guardian, child and trial examiners/team will remain masked regarding trial group allocation.

If the adult/child pair meets eligibility criteria, all teeth that meet the inclusion criteria will be targeted for treatment. Administration of the trial drug/placebo is detailed in Section 6.2 of the protocol as well as the MOP. Photographs may be taken of the child's teeth/mouth but not including the face or other identifying features for illustration and publication purposes. Frankl score, a measurement of child behavior, will also be recorded. All children will receive advice on diet, brushing with a small amount of F toothpaste under supervision, and will receive a toothbrush and fluoride toothpaste. Parents will be advised not to use F toothpaste the evening after the application.

Trial drug/placebo application will not be performed the same day the child is receiving a FV application as part of usual care; time between FV and SDF applications must be at least 12 hours.

7.3 Intermediate Visits

7.3.1 24- to 48-Hour Safety Contacts (±8 hours)

All participants will be contacted 24- to 48-hours after the baseline and 6-month trial visits to inquire if any Adverse Events (AEs), Serious Adverse Events (SAEs), or Unanticipated Problems (UPs) have occurred. This could be via a phone call, email, text, etc., or in person, if possible. All events will be recorded and reported, and the child will be brought in for an unscheduled visit as required. Additionally, the four questions from the Family Survey which refer to treatment acceptability may be asked at this time.

7.3.2 24- to 48-Hour Safety Visits (±8 hours)

In addition to the 24- to 48-hour safety contacts, a safety visit 24- to 48-hours following application of the trial drug/placebo will be available for each participant after the baseline and 6-month trial visits. An intraoral soft tissue examination, including examination of the gingiva, will be performed. The status of the intraoral soft tissues will be recorded, and children with an AE (e.g., gingival irritation), SAE, or UP will be monitored until resolution of the problem or referred for treatment, if necessary. Refer to Appendix E and the MOP for additional details.

7.3.3 1.5-, 4.5-, 7-Month Intermediate Contacts (±10 days)

Intermediate contacts will occur approximately 1.5, 4.5, and 7 months post baseline, and will allow the trial team to ask brief questions to inquire if any AEs, SAEs, or UPs have occurred, in addition to administering the tooth pain questionnaire to determine if an unscheduled trial visit is necessary to

evaluate the child. Additionally, medical conditions may be updated at the 4.5 month intermediate contact in order to confirm eligibility for the second application of SDF/placebo at the 6 month visit. These contacts will occur by a variety of strategies, including via phone, email, online survey, etc. The contacts will help to enhance retention.

All AEs, SAEs, and UPs will be recorded and reported. Trial teeth that cause pain due to caries, pulpal exposure, mobility, signs of pulpal infection (abscess, fistula, swelling) will be exited from the trial and referred for appropriate care (**early exit strategy**), and will be considered treatment failures in the analysis of the efficacy outcome.

7.3.4 Three-Month Visit (±19 days)

Trial participants will be asked to attend a trial visit at the early childhood education program or a central location (may vary by trial site) approximately 3 months post-baseline. This visit will include: an intraoral soft tissue oral exam, a full-mouth ICDAS exam, including an assessment of color and hardness of all cavitated lesions (regardless of whether included or not in the trial), potential photographs of the child’s teeth/mouth but not including the face or other identifying features for illustration purposes, assessment of AEs, SAEs, and UPs, recording Frankl behavior score, and trial questionnaire completion by the parent/legal guardian. If the parents/guardians are not in attendance, parents will be contacted via preferred contact method to obtain questionnaire responses. If a trial tooth meets the criteria for an “early exit strategy”, it will be exited from the trial and referred to appropriate follow-up care. The tooth will be considered a treatment failure in the analysis of the primary endpoint. All children will receive written advice to take home on diet, brushing with a small amount of fluoridated toothpaste under supervision, and will receive a toothbrush and fluoridated toothpaste.

7.3.5 Six-Month Visit (±19 days)

Trial participants will attend a trial visit at the early childhood education program or a central location (may vary by trial site) approximately 6 months post-baseline. This visit will be identical to the 3-Month Visit described in 7.4.4, but in addition, medical history will be either re-assessed or reviewed from the 4.5 month contact to confirm eligibility and then the SDF/placebo will be re-applied to all cavitated trial lesions. Data from the 6-month visit will be used to assess the primary outcome of the trial.

7.4 Final Trial Visit: 8 Months (±19 days)

Trial participants will be asked to attend a final trial visit at the early childhood education program or a central location (may vary by trial site) approximately 8 months post-baseline. This visit will be identical to the 3-Month Visit described in 7.3.4. Additionally, final assessment of the treated teeth will be provided to the family. Follow-up care referrals will be facilitated by the trial team.

7.5 Withdrawal Visit

A withdrawal visit will occur for participants who withdraw or are withdrawn from the trial. The withdrawal visit will mirror the 8-month trial visit. If a participant cannot or will not attend the withdrawal visit, this information will be recorded.

7.6 Unscheduled Visit

In the event there are signs (abscess, fistula, and swelling) or symptoms (pain) of infection at any point, an unscheduled visit will be offered to the parent/legal guardian and child by the trial team. A clinically-warranted examination will be conducted, an assessment for AEs will be performed, and the participant will be referred for treatment if necessary. AEs will be reported per IRB guidelines.

7.7 Unscheduled Contact

Parents/legal guardians of trial children will be instructed to contact the trial team if they have any concerns at any point in the trial (e.g., believe their child is in pain). When the parent contacts the trial team, an assessment for AEs will be performed, the DDQ will be collected, and the participant will be scheduled for an unscheduled visit as necessary. AEs will be reported per IRB guidelines.

8 TRIAL PROCEDURES / EVALUATIONS

8.1 Trial Procedures/Evaluations

8.1.1 Clinical Examination

A clinical assessment will be conducted with each child participant. This will consist of the following.

- Teeth: A full mouth ICDAS examination will be conducted, and lesion hardness will be assessed using the ICDAS II system activity criteria for lesions with score ICDAS 5-6. See Appendix A.

- Intraoral soft tissues: The intraoral soft tissues will be examined for pathology and signs of infection. For details refer to the MOP. The clinical criteria for odontogenic infection are the presence of swelling, dental abscess, or a draining sinus in the tissue closely surrounding the tooth. Assessments for odontogenic infection or other intraoral infections are made at each clinical trial visit and unscheduled/emergency visit. Assessment of mucosal irritancy will be made by the trial examiner or hygienist, using a modification of an oral mucositis scale developed for radiation-induced mucositis (Sonis et al., 1999).

8.1.2 Questionnaires

- Medical history will be assessed at baseline and reviewed at approximately 6 months or between 4.5 and 6 months.

- Assessments for tooth pain are to be made at each trial visit throughout the child's participation in the trial using the Dental Discomfort Questionnaire (DDQ) (Appendix B; Versloot et al., 2006), to be completed by the parent/guardian. More details are provided next: Given that pain is a subjective experience, pain in general is most reliably measured using self-report (FACES pain scale and Wong-Baker FACES scale) in children (Hicks et al., 2001; Baretto et al., 2004; Versloot et al., 2004). Assessing pain in preschoolers presents special challenges, as their cognitive capacities are still under-developed making it difficult for them to communicate verbally, and consequently reliably self-report pain. To avoid inaccurate assessment of pain in very young children, it is recommended to use a validated observational tool that assesses pain as reported by parents/legal guardians as proxies based on the observation of pain-related behaviors by the child. The DDQ (Appendix B; Versloot et al., 2006), a behavioral observation tool developed and validated to recognize toothache in children aged 5 or younger, focuses on toothache-related pain behaviors.

  This validated questionnaire consists of two parts. The first part includes a question concerning the occurrence of toothache. This question can be answered with: 'never', 'sometimes', 'often' or 'I do not know'. If the parent answers 'sometimes' or 'often', they are asked when. Choices include: either during meals, during daytime or nighttime (several alternatives possible). The second part of the DDQ consists of 8 validated questions about different behaviors associated with toothache or discomfort due to caries (e.g., crying during meals or chewing problems). For each item the parent is asked to rate how often their child shows a given specific behavior. The questions can be answered on a three-point scale: 0 'never', 1 'sometimes', and 2 'often'. According to Versloot et al. (2006), 70% of children with cavities and toothache had a DDQ score of 4 or higher, and the average score for these children was 5.73. However, for ethical reasons in this study, any complaint of toothache pain (DDQ score of 1 or higher) will result in the study team attempting an unscheduled visit to examine the child to assess whether the tooth still meets eligibility criteria for the trial or needs referral for care.

Assessments for presence of pain and level of pain (score of the DDQ) will be made at each trial visit, intermediate contact, and unscheduled/emergency visit in the trial using the DDQ. The total number of pain episodes associated with trial teeth per child will be assessed.

- Systemic effects/allergic reaction will be assessed at each trial visit and interim trial contact, either by examiner observation or by soliciting information from parents about the following adverse events: nausea, not eating, vomiting, difficulty swallowing or breathing, swelling around the lips or skin of the face, itchiness around the lips or skin of the face, hives or rash, stomach ache, or diarrhea.

- OHRQoL will be measured at the baseline, 3-, 6-, and 8-month trial visits throughout participation in the trial. Parents/guardians will be asked to complete a 16-item Parental-Caregiver Perception Questionnaire (P-CPQ) and an 8-item Family Impact Scale (FIS) questionnaire (Thomson et al., 2013; Appendix C). The rationale is that for children under six years of age, the use of simple child-completed scales, or questionnaires completed by parents/legal guardians as proxies, is the usual approach. Parents/legal guardians will be asked to complete the 16-item P-CPQ and 8-item FIS questionnaire. These measures have been found to be reliable, valid and responsive in children with severe-ECC, and help measure different aspects of the oral health condition. The P-CPQ estimates the nature and magnitude of the effects of the condition on the child's OHRQoL, considering not just pain, but the impact on their overall well-being (e.g., how they eat, play with others/socialize, look/teeth appearance, etc.). The FIS enables determination of the impact of the child's condition on the family. These scores will be compared at each trial visit against an accepted single-item (global) self-rating or overall well-being question, which serves as a summary measure of the same construct. In addition, a global transition judgment question will be included at follow-up visits for assessing the responsiveness of these measures to SDF treatment in children with ECC (Locker et al., 2004). For details see Appendix C.

- The Family Survey (Appendix D) including questions about treatment acceptability and satisfaction will be completed at baseline/24-48 hour contacts/visits, and 3-, 6-, and 8-month trial visits. Satisfaction with treatment will be assessed using a well-validated tool (the Post-Surgical Patient Satisfaction Questionnaire; Kiyak et al.,1984); and in addition, the Client Satisfaction Questionnaire (which is an 8-item, easily administered and scored instrument designed to assess client satisfaction with services) which has been adapted for this project (Larsen et al., 1979; Attkisson and Zwick, 1982). A question will be asked after the placement of SDF/placebo regarding the acceptability of the treatment (i.e., was the treatment tolerated by the child), as there is evidence that different types of treatment may influence children's behavior and perceptions of their dental treatment (Santamaria et al., 2015).

9 ASSESSMENT OF SAFETY

9.1 Specification of Safety Parameters

Parameters used to assess safety will include AEs spontaneously reported by the participant or observed by the investigator, solicited AEs captured by interviewing the child's parent or legal guardian, and events recorded from oral cavity examination. All AEs will be coded by system organ class and preferred term according to the terminology of the Medical Dictionary for Regulatory Activities (MedDRA®).

Monitoring of AEs will be conducted throughout the trial. All AEs, SAEs, and UPs will be captured any time after informed consent is obtained. In addition, all AEs will be captured until 7 days after the last day of trial participation. Serious adverse events (SAEs) will be captured until 30 days after trial participation. SAEs will be reported to the IND sponsor and to NIDCR within 24 hours of becoming aware of the event. Safety events will be reported to the sIRB according to their policies and guidelines. AEs will be followed until they are resolved, considered stable, the subject is lost to follow-up, or the AE is otherwise explained.

Cavitated caries lesions will have signs of progressing infection (e.g., abscess, fistula, etc.) recorded. Gingival tissue irritation (e.g., ulcerations and mucosal erythema, leukoplakia or darkening of pigmentation) will also be recorded. In addition we will record observed or reported: nausea, not eating, vomiting, difficulty swallowing or breathing, swelling around the lips or skin of the face, itchiness around the lips or skin of the face, hives or rash, stomach ache; or diarrhea.

Potential adverse reactions to the trial drug include

- Infection: Infection will be defined by changes in clinical signs (from examination) over time in each trial arm. The clinical criteria for the positive recording of infection are the clinical presence of swelling, dental abscess, or fistula. Assessments for infection are made at each clinical trial visit and unscheduled/emergency visit. (See Appendix E).
- Mucosal irritancy: children in each group experiencing gingival erythema, ulcerations, etc. will be recorded (See Section 8.1.1 and Appendix E).
- Systemic effects/allergic reaction: children in each group reported or observed to have: nausea, not eating, vomiting, difficulty swallowing or breathing, swelling around the lips or skin of the face, itchiness around the lips or skin of the face, hives or rash, stomach ache, or diarrhea (See Appendix E).

9.1.1 Adverse Events

An adverse event is any untoward or unfavorable medical occurrence in a human subject, including any abnormal sign (for example, abnormal physical exam or laboratory finding), symptom, or disease, temporally associated with the participant's participation in the research, whether or not considered related to the participant's participation in the research.

9.1.2 Serious Adverse Events

A serious adverse event (SAE) is an AE one that meets one or more of the following criteria:

- Results in death
- Is life-threatening (places the participant at immediate risk of death from the event as it occurred)
- Results in inpatient hospitalization or prolongation of existing hospitalization
- Results in a persistent or significant disability or incapacity
- Results in a congenital anomaly or birth defect

- An important medical event that may not result in death, be life threatening, or require hospitalization may be considered an SAE when, based upon appropriate medical judgment, the event may jeopardize the participant and may require medical or surgical intervention to prevent one of the outcomes listed in this definition.

9.1.3 Unanticipated Problems

The Office for Human Research Protections (OHRP) considers unanticipated problems involving risks to participants or others to include, in general, any incident, experience, or outcome that meets **all** of the following criteria:

- unexpected in terms of nature, severity, or frequency given (a) the research procedures that are described in the protocol-related documents, such as the IRB-approved research protocol and informed consent document; and (b) the characteristics of the participant population being studied;
- related or possibly related to participation in the research ("possibly related" means there is a reasonable possibility that the incident, experience, or outcome may have been caused by the procedures involved in the research); and
- suggests that the research places participants or others at a greater risk of harm (including physical, psychological, economic, or social harm) than was previously known or recognized.

9.2 Time Period and Frequency for Event Assessment and Follow-Up

Adverse events will be followed until they have resolved, are considered stable, the participant is lost to follow-up, or the AE is otherwise explained. Phone calls or extra visits may be performed to follow up the AE. The participant will be questioned on the AE and whether it continues or is resolved at the time of the trial visit, and whether the participant received any treatment or medications specifically for the AE. The AE data will be updated and a follow-up report will be submitted to the IND Sponsor, NIDCR, and the sIRB according to the requirements of each.

At the last scheduled visit, the investigator will instruct each participant to report any subsequent event(s) that the participant, or the participant's personal physician, believes might reasonably be related to participation in this trial. The investigator will notify the IND sponsor, NIDCR, and the sIRB of any death or AE occurring at any time after a participant has discontinued or terminated trial participation, if the death or AE may reasonably be related to this trial.

9.3 Characteristics of an Adverse Event

All AEs must be graded for relationship to trial product, expectedness, and severity.

9.3.1 Relationship to Trial Intervention

The clinician's assessment of an AE's relationship to test article is part of the documentation process, but it is not a factor in determining what is or is not recorded in the trial database. If there is any doubt as to whether a clinical observation is an AE, the event should be recorded. All AEs will be recorded and reported periodically to the DSMB, NIDCR, and IND sponsor, and only those which meet the definition of an unanticipated problem will be reported to the sIRB within 5 days. All AEs must have their relationship to trial product assessed using the following terms:

1. Related (Possible, Probable, Related)
    a. The event is known to occur with the trial intervention.
    b. There is a temporal relationship between the intervention and event onset.
    c. The event abates when the intervention is discontinued.

d. The event reappears upon a re-challenge with the intervention.

2. Not Related (Unlikely, Not Related)
    a. There is no temporal relationship between the intervention and event onset.
    b. An alternate etiology has been established.

9.3.2 Expectedness of AEs

The Trial PI will be responsible for determining whether an AE is expected or unexpected. An adverse event will be considered unexpected if the nature, severity, or frequency of the event is not consistent with the risk information previously described for the intervention.

9.3.3 Severity of Event

Severity of AEs will be graded according to the following scale:

- Mild: no intervention required; no impact on activities of daily living
- Moderate: minimal, local, or noninvasive intervention indicated; moderate impact on activities of daily living
- Severe: significant symptoms requiring invasive intervention; participant seeks medical attention, needs major assistance with activities of daily living. This also includes events that are considered life-threatening or may result in death.

9.4 Reporting Procedures

9.4.1 Unanticipated Problem Reporting to IRB and NIDCR

Incidents or events that meet the OHRP criteria for unanticipated problems require the creation and completion of an unanticipated problem report form. OHRP recommends that investigators include the following information when reporting an adverse event, or any other incident, experience, or outcome as an unanticipated problem to the IRB:

- appropriate identifying information for the research protocol, such as the title, investigator's name, and the IRB project number;
- a detailed description of the adverse event, incident, experience, or outcome;
- an explanation of the basis for determining that the adverse event, incident, experience, or outcome represents an unanticipated problem;
- a description of any changes to the protocol or other corrective actions that have been taken or are proposed in response to the unanticipated problem.

To satisfy the requirement for prompt reporting, unanticipated problems that are not SAEs will be reported using the following timeline:

- Unanticipated problems will be reported to the sIRB within 5 days of the investigator becoming aware of the event.
- All unanticipated problems should be reported to appropriate institutional officials (as required by an institution's written reporting procedures), the supporting agency head (or designee), and OHRP within one month of the IRB's receipt of the report of the problem from the investigator.

Concurrently with reporting to the IRB, all unanticipated problems will be reported to the NIDCR via Rho Product Safety (EDC notification).

General questions about UP reporting to the NIDCR can be directed to the Rho Product Safety Help Line (available 8:00AM – 5:00PM Eastern Time):

- US: 1-888-746-7231
- International: 919-595-6486

In the event EDC is not available, the site Investigator should call Rho Product Safety to report the event, then submit a paper UP form via email or fax. Once EDC is operational, the Investigator is to enter the UP data into the EDC system within 24 hours of regained functionality.

9.4.2 Serious Adverse Event Reporting to IND Sponsor and NIDCR

Any AE meeting the specified Serious Adverse Event criteria, whether related or unrelated, will be communicated to the IND sponsor via telephone within 24 hours of site awareness:

SAE Reporting Contact Information:

- IND Sponsor: 206-251-6831

Concurrently with reporting to the IND Sponsor, the Investigator will submit an SAE form (eCRF) to the NIDCR via Rho Product Safety (EDC notification). Once submitted, Rho Product Safety will send a confirmation email to the investigator within 1 business day. The Investigator should contact Rho Product Safety if this confirmation is not received. This process applies to both initial and follow-up SAE reports.

In the event EDC is not available, the site Investigator should call Rho Product Safety to report the event, then submit a paper SAE form via email or fax. Once EDC is operational, the Investigator is to enter the SAE data into the EDC system within 24 hours of regained functionality.

SAE Reporting Contact Information:

- Product Safety Fax Line (US): 1-888-746-3293
- Product Safety Fax Line (International): 919-287-3998
- Product Safety Email: rho_productsafety@rhoworld.com

General questions about SAE reporting to the NIDCR can be directed to the Rho Product Safety Help Line (available 8:00AM – 5:00PM Eastern Time):

- US: 1-888-746-7231
- International: 919-595-6486

All SAEs will be followed until resolution or stabilization.

9.4.3 Reporting of Safety Events to FDA

Following notification from the investigator, the IND sponsor will notify FDA of any unexpected fatal or life-threatening suspected adverse reaction within 7 calendar days after the sponsor's initial receipt of the information. The IND sponsor will notify the FDA and all investigators of 1) serious, unexpected suspected adverse reactions, 2) findings from other clinical, animal or in-vitro studies that suggest significant human risk, and 3) a clinically important increase in the rate of a serious suspected adverse reaction no later than 15 calendar days after determining that the information qualifies for reporting. All serious events designated as “not related” to trial product will be reported to the FDA at least annually in a summary format.

9.5 Halting Rules

An adverse event graded as severe that is considered to be related to trial participation will be immediately reported to the Medical Monitor and will trigger an immediate investigation to determine the next steps for the child and/or trial.

The trial may be stopped early if interim analyses provide early determination of efficacy or futility. Sample size calculations account for the use of an early stopping rule based on efficacy or futility. The interim analysis will be performed after approximately half of the sample has completed the 6-month visit.

10 TRIAL OVERSIGHT

In addition to the PI's responsibility for oversight, trial oversight will be under the direction of a Data and Safety Monitoring Board (DSMB) composed of members with appropriate expertise in pediatric dentistry, pediatrics, statistics, ethics, and other scientific disciplines. Members will be appointed by NIDCR. A copy of the DSMB charter will be provided to the FDA. The DSMB will meet at least annually to assess safety and efficacy data, trial progress, and data integrity for the trial. If safety or data integrity concerns arise, more frequent meetings may be held. The DSMB will operate under the rules of an NIDCR-approved charter that will be approved at the organizational meeting of the DSMB. The DSMB will provide recommendations to the NIDCR.

11 CLINICAL SITE MONITORING

Clinical site monitoring is conducted to ensure that the rights of human subjects are protected, that the trial is implemented in accordance with the protocol and/or other operating procedures, and that the quality and integrity of trial data and data collection methods are maintained. Monitoring for this trial will be performed by a monitor from Rho, Inc. as a subcontract from NIDCR. The monitor will evaluate trial processes and documentation based on NIDCR standards and the International Conference on Harmonisation (ICH), E6: Good Clinical Practice guidelines (GCP).

Details of clinical site monitoring will be documented in a Clinical Monitoring Plan (CMP) developed by Rho, Inc., in collaboration with the NIDCR Office of Clinical Trials and Operations Management (OCTOM) and the NIDCR Program Official. The CMP will specify the frequency of monitoring, monitoring procedures, the level of clinical site monitoring activities (e.g., the percentage of participant data to be reviewed), and the distribution of monitoring reports. Some monitoring activities may be performed remotely, while others will take place at the trial site(s). The Rho monitor will conduct monitoring activities and provide reports of the findings and associated action items in accordance with the details described in the CMP. Documentation of monitoring activities and findings will be provided to the site trial team, the trial PIs, OCTOM, and the NIDCR. The NIDCR reserves the right to conduct independent audits as necessary.

12 STATISTICAL CONSIDERATIONS

Detailed statistical procedures, listings, table shells, and figures will be provided in a separate Statistical Analysis Plan (SAP) that will be finalized before trial close-out and database lock.

12.1 Trial Hypotheses

The Primary Hypothesis:

- 38% Silver Diamine Fluoride (SDF) will arrest cavitated caries lesions (ICDAS 5 or 6) in primary teeth, measured by a change in dentin hardness, without a change in ICDAS severity score, after a single application (measured at approximately 6 months post initial application). This occurs as a result of the halting of the caries disease process, which occurs very soon after application of SDF due to its strong antimicrobial and remineralizing properties.

The Secondary Hypotheses:

- 38% Silver Diamine Fluoride (SDF) will arrest a greater proportion of cavitated caries lesions (ICDAS 5 or 6) in primary teeth after two applications, approximately 6 months apart (measured at approximately 8 months after initial SDF application).
- Lesions arrested approximately 3 months after a single application will remain stable when re-assessed at approximately 6 months.
- 38% Silver Diamine Fluoride (SDF) will decrease the proportion of children in pain, number of pain episodes associated with treated teeth for children, and pain score associated with treated teeth. The hypothesis is that SDF will do this as it is an anti-hypersensitivity agent, extremely efficient at blocking dentinal tubules, and also because it can arrest cavitated lesions, and thus decrease pain.

The Tertiary Hypotheses:

- 38% Silver Diamine Fluoride (SDF) will increase Oral Health-Related Quality of Life (OHRQoL) as a result of decreasing pain and arresting caries lesions.
- 38% Silver Diamine Fluoride (SDF) will be an acceptable treatment by parents/guardians of young children.

12.2 Sample Size Considerations

The population will be children 12-71 months of age at the baseline caries examination attending early childhood education programs or recruited from clinics associated with the Michigan, Iowa or New York clinical trial sites. We anticipate 10% attrition over the trial period, as we will follow children over approximately 8 months.

The primary outcome of this trial is the proportion of arrested caries lesions approximately 6 months from the baseline visit (i.e., lesions that have changed from soft to hard over the trial period according to the ICDAS II activity criteria). A 10% difference in the proportion of arrested caries lesions between the SDF and placebo groups will be considered clinically significant. Prior studies in the literature of SDF using this outcome in primary cavitated lesions have shown a wide range for the proportion of arrested caries lesions for both SDF-treated and placebo-treated or untreated lesions; therefore the 10% difference in proportions was calculated assuming 55% versus 45% arrested caries lesions for SDF compared to placebo. In prior studies, an average of 4 lesions was treated per participant, with a within-participant correlation of 0.5; we are conservatively assuming 3 lesions per participant. The sample size calculations used a 2-sided test for a difference in proportions with cluster randomization (participant as the cluster) at significance level α=0.1%, with 80% power to detect a superiority difference of 10% for SDF compared to placebo. The calculations also account for the use of an early stopping rule for efficacy and futility. The interim analysis will be performed after approximately half of the sample has completed the 6-month

examinations. A Lan-Demets spending function with an O'Brien-Fleming type boundary will be used, with p-values for efficacy at 0.000001709 and 0.00099938 at 50% and 100% completion, respectively. Non-binding p-values for stopping due to futility at 50% and 100% completion will be 0.2336928 and 0.00099938, respectively. With the above assumptions, the trial will require 1144 participants to be enrolled. Sample size calculations were made using East version 6.

Sample size for the 24-48 h post-treatment safety visit is justified based on the 95% upper bound for the difference in 'safety event' between SDF and control. Erythema and 'adverse event' rate in previous studies are around 10-15% in the controls. Thus, assuming no safety difference between the two groups, and events occurring in 15% of the participants, the study sample will produce an upper bound for the difference at most 4% above the observed difference. Sample size calculations were made using PASS 13.

12.3 Planned Interim Analyses

Interim Analyses: A masked interim analysis of the primary study outcome will be performed after approximately half of the sample has completed the 6-month examination. A Lan-Demets spending function with an O'Brien-Fleming type boundary will be used, with p-values for efficacy at 0.000001709 and 0.00099938 at 50% and 100% completion, respectively. Non-binding p-values for stopping due to futility at 50% and 100% completion will be 0.2336928 and 0.00099938, respectively.

12.3.1 Safety & Efficacy Review

Safety-related stopping rules for the trial as outlined in Section 9.5 would occur due to participant safety concerns.

The trial may be terminated for early efficacy or futility based on the results of an interim analysis, as described in the Statistical Analysis Plan. The interim analysis will be conducted after approximately half of the participants have completed the 6-month examination.

The DSMB, NIDCR, and IRBs would be consulted regarding stopping the trial.

12.4 Final Analysis Plan

We will consider all relevant biological variables (e.g., gender, race/ethnicity, and age) in the analyses. The initial analyses will involve exploration of the data. Continuous variables may be transformed or categorized based on the observed distribution. Categorical variable response categories may be collapsed due to small sample sizes. Whether race and ethnicity are analyzed separately or as a single variable depends upon whether the Hispanics in the trial are primarily White or if they also include Black Hispanics. Because randomization is stratified by trial site, trial site will be included as a covariate in all analyses.

Aim 1 (Cavitated caries lesion arrest at 6 months follow-up): The proportion of arrested cavities at the 6-month visit will be compared between the SDF and placebo-treated groups first using a generalized estimating equation (GEE) model for binary outcomes, using an exchangeable correlation to account for possible non-independence among multiple cavities within a participant. Trial site will be included as a covariate.

Secondary analyses of the proportion of arrested lesions will extend the GEE model for binary outcomes to include covariates and their interactions with the treatment effect; covariates will include race/ethnicity, gender, age, trial site, initial ICDAS score, tooth location (anterior/posterior; as there are some data in the literature to suggest a difference in SDF efficacy) and number of affected teeth. Interactions of the

covariates with treatment will be explored to examine the results for differential treatment effects. However, the analyses are not specifically powered to detect these interactions.

Note: As this trial may enroll siblings, at the time of analyses we intend to explore the correlations within participants and correlations between siblings to aid in determining the appropriate modeling technique to use. As described above the current analysis plan uses GEE models to account for correlations among multiple cavities within a participant. The GEEs could be extended to account for siblings by correlating all cavities within a family, rather than within a participant. However, this assumes the same within family correlation. An alternative approach is to account for the two levels of correlations (cavities within participants, participants within families) by switching the analyses to use generalized linear mixed models (GLMM) instead of GEE. One difficulty we anticipate with a switch to GLMM is estimation of the random effects – the number of families with enrolled siblings may not be large enough to properly estimate the two random effects within the GLMMs.

Aim 2 (Cavitated caries lesion arrest at 8 months follow-up): The same GEE model for binary outcomes as used for Aim 1 will be used to compare the proportion of arrested lesions at approximately 8-months to examine Aim 2.

Sub-Aim 2a (Compare the effect of one application of 38% SDF at 3 and 6 months follow-up on caries lesion arrest): A longitudinal (repeated measures) model for binary data that incorporates both the correlation within each subject at each time and across the time points will be used to compare the proportion of arrested caries lesions at approximately 3 and 6 months. Trial site will be included as a covariate.

Aim 3 and Sub-Aim 3a (pain): At the 3-, 6-, and 8-month visits, the proportion of participants who have experienced pain with treated teeth during the follow-up period will be compared between the SDF and placebo-treated groups using Cochran-Mantel-Haenszel chi-square tests, with trial sites as the strata.

The total number of episodes of pain associated with trial teeth per participant (calculated by adding all reports of pain throughout the trial verified by a clinical exam to be associated with a trial tooth) during the follow-up period will be compared between the SDF and placebo treated groups using generalized linear models (GLMs) for count data, with trial site included as a covariate. The DDQ score will be compared between groups using analysis of covariance at each follow-up visit, with trial site included as a covariate.

Additional GLMs for binary and count outcomes and analysis of covariance models for continuous outcomes, as appropriate for each outcome above, will analyze the effects of covariates and their interactions with the treatment effect.

Aim 4 (Family-level outcomes): The P-CPQ, FIS, and satisfaction and acceptability scores will be compared between the SDF and placebo-treated groups using analysis of covariance, with trial site included as a covariate .

Additional analyses will consider the effects of covariates and their interactions with the treatment effect.

Changes in the color of treated lesions over time will be compared between treatments using a Cochran-Mantel-Haenszel chi-square test, with trial sites as the strata.

The family's experiences with ECC, child's brushing and dietary habits, and color change will be examined as additional covariates for the analyses of satisfaction and acceptability scores.

Safety: The proportion of infected trial teeth at each visit will be compared between the SDF and placebo-treated groups using a generalized estimating equation (GEE) model for binary outcomes, using an exchangeable correlation to account for possible non-independence among multiple teeth within a participant. Trial site will be included as a covariate.

The proportion of participants with adverse events (gum infection, oral mucositis, allergic reaction, anaphylaxis, nausea, vomiting, gastrointestinal pain, diarrhea; individually and the presence of any of the events) during the follow-up period will be compared between the SDF and placebo-treated groups using Cochran-Mantel-Haenszel chi-square tests, with trial sites as the strata.

12.4.1 Analysis populations

The primary analysis will be conducted using the intent-to-treat (ITT) population. The ITT population will include all randomized subjects. Analyses will also be performed using the per-protocol (PP) population. The PP population will be limited to subjects who complete all dental examinations and surveys/questionnaires for the baseline, 3-month, 6-month, and 8-month visits within the specified study windows and complete the intermediate contacts at 1.5, 4.5, and 7 months within the specified study windows.

12.4.2 Missing data

As noted in Section 5.5, any lesions removed from the trial due to the "early exit strategy" will be considered to be treatment failures – non-arrest of the lesion and presence of pain and/or infection requiring removal of the tooth from the study – and not considered to be missing data.

The primary method for handling missing data will be to use a multiple imputation analysis, with imputations based on race/ethnicity, gender, age, trial site, initial ICDAS score, tooth location, treatment, and most recent prior visit results will be performed. Twenty-five imputed datasets will be created, and results will be combined using standard methods.

A sensitivity / tipping point analysis will also be performed to evaluate the MAR (missing at random) assumption for the missing primary efficacy outcome (caries arrest at approximately 6 months). A pattern-mixture approach to the missing data imputation (missing not at random, MNAR) will be applied to the logistic regression model used for imputing the missing caries arrest at approximately 6 months outcome from the variables described above. To represent MNAR, a range of shift parameters will be used to adjust the log odds ratios for the SDF-treated group. The tipping point will be identified as the amount of shift needed to change the statistical significance of the treatment comparison using the primary efficacy analysis. The size of the shift required to change the statistical significance will be used to gauge the plausibility of the MAR assumption.

13 SOURCE DOCUMENTS AND ACCESS TO SOURCE DATA/DOCUMENTS

Data will be collected on a combination of paper source documents and on electronic Case Report Forms (eCRF). When data is directly entered into the clinical database the eCRF will serve as the source document. Data will include ICDAS examination data, safety data, and parent/guardian survey responses to address tertiary objectives. A commercially-developed, FDA-compliant, web-based electronic data capture system will be utilized for this trial.

Trial staff will maintain appropriate medical and research records for this trial, in compliance with ICH E6, Section 4.9 and regulatory and institutional requirements for the protection of confidentiality of participants. Trial staff will permit authorized representatives of NIDCR and regulatory agencies to examine (and when required by applicable law, to copy) research records for the purposes of quality assurance reviews, audits, and evaluation of the trial safety, progress and data validity.

14 QUALITY CONTROL AND QUALITY ASSURANCE

Each clinical site is responsible for maintaining quality control at their site. This includes managing the security, integrity and completeness of items such as Case Report Forms, consents, and medical histories. Any data stored electronically must be handled in accordance with federal institutional policies. The Project Manager will provide oversight of Quality Management (QM). The Project Manager will work with the sites (recruitment centers) to develop plans for appropriate internal quality management that incorporate elements of quality control and quality assurance. These quality management plans will provide site staff with the means to identify and resolve problems with protocol implementation and regulatory compliance.

To ensure consistency in ICDAS examinations across different examiners and trial sites, an examiner calibration session will occur prior to trial initiation. All dental examiners will be trained and calibrated in the use of the ICDAS II criteria for dental caries, including assessment of dentin hardness and color in cavitated lesions. Examiners will also be trained in the management and examination of young children.

The recruitment centers should follow their institutional SOPs, where relevant, to conduct the trial per protocol for Quality Assurance. The SOPs should be available to the trial monitor during monitoring visits.

14.1 Participant Binder

All essential trial documents must be retained by the investigator in a Participant Binder and generally include the following:

- Source documents
- Paper questionnaires completed by the participant
- Paper CRFs
- Data correction forms

14.2 Regulatory Binder

All essential regulatory documents must be maintained by the investigator. These generally include the following:

- IRB documents, including approved consent(s)
- Investigator and Sub-Investigator CVs or documentation of qualifications and training
- Delegation of Responsibility Log, and
- Monitoring Reports

15 ETHICS/PROTECTION OF HUMAN SUBJECTS

15.1 Ethical Standard

The investigator will ensure that this trial is conducted in full conformity with the principles set forth in The Belmont Report: Ethical Principles and Guidelines for the Protection of Human Subjects of Research, as drafted by the US National Commission for the Protection of Human Subjects of Biomedical and Behavioral Research (April 18, 1979) and codified in 45 CFR Part 46 and/or the ICH E6.

15.2 Institutional Review Board

The protocol, informed consent form(s), recruitment materials, and all participant facing materials will be submitted to the single IRB (sIRB) of record for review and approval. Additionally, all trial sites will submit institutional IRB submissions to cede to a single IRB.

Approval of both the protocol and the consent form must be obtained before any participant is enrolled. Any amendment to the protocol will require review and approval by the IRB before the changes are implemented in the trial.

15.3 Informed Consent Process

A waiver of informed consent and HIPAA will be requested to access potential child participant names, telephone numbers, addresses, and dental records to pre-screen for eligibility. This information will be used to introduce the trial to parents/legal guardians of children who may be eligible to participate. If the parent/guardian is interested, her/his consent will be obtained. See below for more detail on these processes.

Parents/legal guardians of children with potentially eligible lesions will be informed about the trial, and if interested will undergo the consenting process. If pre-screening happens during the school day, when parents/guardians are not present, the pre-screening results and information about the trial, and invitation to schedule a baseline visit, will be sent home with the child. Interested families will call the trial team or will receive a call from the trial team and will undergo review of the inclusion/exclusion criteria over the phone, and receive a brief explanation of the trial. If they agree to participate, the consenting process will occur.

Participant Consent: The consent forms will be reviewed and informed consent will be obtained prior to any trial activities. The parent/guardian must consent for the child and her/himself. Participants will be given a copy of the signed consent form.

How it will be determined whether the participants understand the information that was provided in the consent document: We will determine whether the participants understand the information that is provided in the consent document by reviewing it in detail with the participant, answering any and all questions the participant may have, and asking the participant to explain the trial back to the person obtaining consent. Participants will be asked to reiterate in their own words what they will need to do as participants in the trial and what the potential risks and benefits are. If participants do not ask any questions about the document, but appear confused or unclear about what was explained to them, then the person obtaining consent will attempt to identify the area(s) of uncertainty and clarify the content for the research participant.

For participants who may prefer the use of other languages, trial materials will be translated by a translator into selected languages, and available translated versions of the consent will be used. In addition, trial personnel fluent in selected languages will be available at sites if needed.

A record of consented participants will be kept in the regulatory binder. The Data and Clinical Coordinating Center (DCCC) will also keep a record of trial ID of those who provided consent.

15.4 Exclusion of Women, Minorities, and Children (Special Populations)

Include	*Exclude*	*Vulnerable Population Type*
	X	*Adults unable to consent*
X		*Individuals who are not yet adults (e.g., infants, children, emancipated minors)*
	X	*Wards of the State (e.g., foster children)*
X		*Pregnant women*
	X	*Prisoners*

Inclusion of Women
No potential participants will be excluded from participation based on their gender. It is anticipated that a roughly equal number of boys and girls ages 12 to 71 months will be included in this trial. It is also anticipated, based on our other studies that the person identified and recruited as the parent/legal guardian in most cases will be the mother.

Inclusion of Minorities
The aim of this trial is to decrease disparities in young, diverse (ethnicity, race, location-urban, rural) U.S. children with high risk for dental caries, by conducting a multi-site longitudinal prospective trial. Thus, inclusion of minorities is crucial to this project's aim. Ethnicity and race of the caregiver and child will be identified by the caregiver.

15.5 Participant Confidentiality

Participant confidentiality is strictly held in trust by the investigators, trial staff, and the sponsor(s) and their agents.

The trial protocol, documentation, data, and all other information generated will be held in strict confidence. No information concerning the trial or the data will be released to any unauthorized third party without prior written approval of the sponsor.

The trial monitor or other authorized representatives of the sponsor may inspect all trial documents and records required to be maintained by the investigator. The clinical trial site will permit access to such records.

Any data, forms, reports, and other records that leave the university that collected the data will be de-identified of any protected health information (PHI) and replaced with trial identifier to maintain participant confidentiality. However, as many trial visits will occur off-site (e.g., head start centers, other dental clinics, etc.), trial teams will need to carry parent/legal guardian and child names with them at those times to identify the child's PIN for data collection. Information will not be released without written permission of the participant, except as necessary for: monitoring by the IRB, OHRP, NIH or its designee and/or any other government officials; and safety monitors/committees that may need the information to make sure that the trial is done in a safe and proper manner, and/or analyze the results of the trial.

To further protect the privacy of trial participants, the Secretary, Health and Human Services (HHS), has issued a Certificate of Confidentiality (CoC) to all researchers engaged in biomedical, behavioral, clinical or other human subjects research funded wholly or in part by the federal government. Recipients of NIH-funded human subjects research are required to protect identifiable research information from forced disclosure per the terms of the NIH Policy (see *https://humansubjects.nih.gov/coc/index*). As set forth in *45 CFR Part 75.303(a)* and *NIHGPS Chapter 8.3*, recipients conducting NIH-supported research covered by this Policy are required to establish and maintain effective internal controls (e.g., policies and procedures) that provide reasonable assurance that the award is managed in compliance with Federal statutes, regulations, and the terms and conditions of award. It is the NIH policy that investigators and others who have access to research records will not disclose identifying information except when the participant consents or in certain instances when federal, state, or local law or regulation requires disclosure. NIH expects investigators to inform research participants of the protections and the limits to protections provided by a Certificate issued by this Policy.

15.6 Future Use of Stored Specimens and Other Identifiable Data

The informed consent document will request permission from the parent/legal guardian to contact him/her for potential future studies. The parent/legal guardian will either consent to allow contact from the trial team to assess interest in potential future studies or will decline.

16 DATA HANDLING AND RECORD KEEPING

The investigators are responsible for ensuring the accuracy, completeness, legibility, and timeliness of the data reported. All source documents should be completed in a neat, legible manner to ensure accurate interpretation of data. The investigators will maintain adequate case histories of trial participants, including accurate case report forms (CRFs), and source documentation.

16.1 Data Management Responsibilities

The University of Michigan in collaboration with Rho, Inc. will serve as the Data and Clinical Coordinating Center (DCCC) for this trial. Data collection and accurate documentation are the responsibility of the trial staff under the supervision of the investigator, lead site coordinator, and project manager. All source documents must be reviewed by the trial team and data entry staff, which will ensure that they are accurate and complete. Unanticipated problems and adverse events must be reviewed by the investigator or designee in a timely manner (please see the MOP for more details).

16.2 Data Capture Methods

Data will be captured via paper source documents or directly entered into the electronic database, with the eCRF serving as the source document for directly entered data.

16.3 Types of Data

The material collected from participants will include data from clinical exams and trial questionnaires, specifically:

- Data collected during the trial pre-screening visit (recruitment) will include eligibility criteria, demographics such as sex, race/ethnicity, age, etc.
- At baseline/24-48 hour contacts/visits, and the 3-month, 6-month, and 8-month clinical trial visits, trial questionnaires including, for example dental discomfort (Appendix B), oral health-related quality of life (Appendix C), and family survey (Appendix D) will be collected. An oral soft tissue exam will be completed. A medical history will be collected at baseline and re-assessed at approximately 6 months or between 4.5 month and 6 month visits. The ICDAS examination of the oral cavity will include scoring of teeth present and absent, as well as presence of caries lesion severity and activity (Appendix A). Cavitated lesions' data for ICDAS score of 5 or 6 will further include hardness and color. For SDF/placebo application (during baseline and 6 month visits), time of application will be recorded.
- Data regarding Adverse and Serious Adverse Events as well as Unanticipated Problems will be collected and reported as needed throughout the trial.
- During the 1.5-, 4.5-, and 7-month Intermediate Contacts, contact information will be updated. Additionally, brief questions inquiring about side effects of the SDF/placebo treatment will be asked, in addition to administration of the dental discomfort questionnaire (Appendix B) to determine if an unscheduled clinic visit is necessary to evaluate the child. Contact information will also be updated during the in person 3- and 6-month trial visits.

16.4 Schedule and Content of Reports

Enrollment and retention data will be reviewed on a regular basis by the Executive Committee and NIDCR. The Executive Committee will be led by the trial PI, and will include the Project and Data Manager, the site PIs, the site lead coordinators, the trial biostatisticians, the NIDCR representatives, and Rho representatives. The Committee will review AEs/SAEs/UPs, reports to the Data and Safety Monitoring Board (DSMB), and interim analysis upon conclusion of the 6-month visits for approximately 50% of the total sample.

16.5 Trial Records Retention

Trial records will be maintained for at least three years from the date that the grant federal financial report (FFR) is submitted to the NIH (or 2 years following the date that SDF is approved for the drug indication for which it is being investigated, whichever is longer).

Trial documents should be retained for a minimum of 2 years after the last approval of a marketing application in an ICH region and until there are no pending or contemplated marketing applications in an ICH region or until at least 2 years have elapsed since the formal discontinuation of clinical development of the investigational product. These documents should be retained for a longer period, however, if required by local regulations. No records will be destroyed without the written consent of the sponsor, if applicable. It is the responsibility of the sponsor to inform the investigator when these documents no longer need to be retained.

16.6 Protocol Deviations

A protocol deviation is any noncompliance with the clinical trial protocol or Good Clinical Practice requirements. The noncompliance may be on the part of the participant, the investigator, or trial staff. As a result of deviations, corrective action plans are to be developed by the trial staff and implemented promptly.

Protocol deviations include, but are not limited to the following:

- Outcome measurement (e.g., caries exam) not performed, trial drug/placebo not applied
- Missed visit, or visit out of window

In addition, non-compliance issues or violations that will be expected to be reported include, but may not be limited to:

- Failure to obtain participant Informed Consent
- Failure to obtain IRB approval and keep IRB approval up to date
- Failure to follow IRB recommendations

These practices are consistent with investigator and sponsor obligations in ICH E6:

- Compliance with Protocol, Sections 4.5.1, 4.5.2, 4.5.3, and 4.5.4.
- Quality Assurance and Quality Control, Section 5.1.1
- Noncompliance, Sections 5.20.1 and 5.20.2.

All deviations from the protocol must be addressed in trial participant source documents and will be periodically reported to NIDCR and the local IRB, according to their requirements. Protocol deviations and violations will be recorded in the eCRFs. Additionally, all protocol deviations and violations will be routinely reported to the DSMB.

17 PUBLICATION/DATA SHARING POLICY

This trial will comply with the *NIH Public Access Policy*, which ensures that the public has access to the published results of NIH funded research. It requires scientists to submit final peer-reviewed journal manuscripts that arise from NIH funds to the digital archive PubMed Central upon acceptance for publication.

The International Committee of Medical Journal Editors (ICMJE) member journals have adopted a clinical trials registration policy as a condition for publication. The ICMJE defines a clinical trial as any research project that prospectively assigns human subjects to intervention or concurrent comparison or control groups to trial the cause-and-effect relationship between a medical intervention and a health outcome. Medical interventions include drugs, surgical procedures, devices, behavioral treatments, process-of-care changes, and the like. Health outcomes include any biomedical or health-related measures obtained in patients or participants, including pharmacokinetic measures and adverse events. The ICMJE policy requires that all clinical trials be registered in a public trials registry such as *ClinicalTrials.gov*, which is sponsored by the National Library of Medicine. Other biomedical journals are considering adopting similar policies. For interventional clinical trials performed under NIDCR grants and cooperative agreements, it is the grantee's responsibility to register the trial in an acceptable registry, so the research results may be considered for publication in ICMJE member journals. The ICMJE does not review specific studies to determine whether registration is necessary; instead, the committee recommends that researchers who have questions about the need to register err on the side of registration or consult the editorial office of the journal in which they wish to publish.

U.S. Public Law 110-85 (Food and Drug Administration Amendments Act of 2007 or FDAAA), Title VIII, Section 801 mandates that a "responsible party" (i.e., the sponsor or designated principal investigator) register and report results of certain "applicable clinical trials:"

Trials of Drugs and Biologics: Controlled, clinical investigations, other than Phase I investigations, of a product subject to FDA regulation;

Trials of Devices: Controlled trials with health outcomes of a product subject to FDA regulation (other than small feasibility studies) and pediatric post market surveillance studies.
NIH grantees must take specific *steps to ensure compliance* with NIH implementation of FDAAA.

18 LITERATURE REFERENCES

American Academy of Pediatric Dentistry Definition of Early Childhood Caries and severe Early Childhood Caries 2008; http://www.aapd.org/assets/1/7/D_ECC.pdf. Accessed December 2016.

American Academy of Pediatric Dentistry Policy on the Use of Deep Sedation and General Anesthesia in the Pediatric Dental Office 2012; http://www.aapd.org/media/policies_guidelines/p_sedation.pdf. Accessed June 2016.

American Dental Association (2012). 2010 Survey of Dental Practice. Characteristics of Dentists in Private Practice and their Patients. Accessed at: https://www.ada.org/en/~/media/ADA/Science%20and%20Research/Members/10_sdpc

American Dental Association Definition of Early Childhood Caries 2000; http://www.ada.org/en/about-the-ada/ada-positions-policies-and-statements/statement-on-early-childhood-caries. Accessed December 2016.

Anderson L, Martin NR, Burdick A, Flynn RT, Blaney DD. Oral health status of New Hampshire Head Start children, 2007-2008. J Public Health Dent. 2010 Summer;70(3):245-8.

Association for State and Territorial Dental Directors. Basic Screening Survey, June 2015.

Attkisson CC, Zwick R. The client satisfaction questionnaire. Psychometric properties and correlations with service utilization and psychotherapy outcome. Eval Program Plann 1982;5(3):233-7.

Barrêtto EPR, Ferreira EF, Pordeus IA. Evaluation of toothache severity in children using a visual analog scale of faces. Pediatr Dent 2004;26(6):485-91.

Berkowitz RJ. Causes, Treatment and Prevention of Early Childhood Caries: A Microbiologic Perspective. J Can Dent Assoc 2003;69(5):304–7.

Besinis A, Deperalta T, Handy R. Antibacterial and antibiofilm activity of a silver nanoparticles dentine coating. J Dent Res 2013;92(Spec Iss A):1646.

Castillo JL, Rivera S, Aparicio T, Lazo R, Aw TC, Mancl LL, Milgrom P. The short-term effects of diammine silver fluoride on tooth sensitivity: a randomized controlled trial. J Dent Res. 2011;90(2):203-208

Chu CH, Lo ECM, Lin HC. Effectiveness of silver diamine fluoride and sodium fluoride varnish in arresting dentin caries in Chinese pre-school children. J Den Res 2002;81(11):767–770.[PubMed]

Dye BA, Tan S, Smith V, Lewis BG, Barker LK, Thornton-Evans G, Eke PI, Beltran-Aguilar ED. Trends in oral health status: United States, 1988–1994 and 1999–2004. National Center for Health Statistics. Vital Health Stat 2007;11(248):1-92.

Dye BA, Thornton-Evans G, Li X, Iafolla TJ. Dental caries and sealant prevalence in children and adolescents in the United States, 2011-2012. NCHS Data Brief. 2015;191:1-8.

Food and Drug Administration (FDA). FDA Drug Safety Communication: FDA review results in new warnings about using general anesthetics and sedation drugs in young children and pregnant women. Dec, 2016. Accessed at: http://www.fda.gov/Drugs/DrugSafety/ucm532356.htm

Gao SS, Zhang S, Mei ML, Lo EC, Chu CH. Caries remineralisation and arresting effect in children by professionally applied fluoride treatment - a systematic review. BMC Oral Health. 2016a;16(1):12. doi: 10.1186/s12903-016-0171-6.

Gao SS, Zhao IS, Hiraishi N, Duangthip D, Mei ML, Lo ECM, Chu CH. Clinical Trials of Silver Diamine Fluoride in Arresting Caries among Children: A Systematic Review. JDR Clinical and Translational Research 2016b;1(3):201-210.

Gonzalez-Cabezas C, Flannagan S. Impact of different fluoride varnish formulations on enamel demineralization prevention. IADR General Session and Exhibition (June 25-28, 2014) (Capetown, South Africa)

Gonzalez-Cabezas C, Flannagan S. Enamel Fluoride Uptake from Varnishes with Different Fluoride Release. IADR/AADR/CADR General Session & Exhibition (March 11-14, 2015) (Boston, Massachusetts)

Hicks CL, von Baeyer CL, Spafford P, van Korlaar I, Goodenough B. The Faces Pain Scale-Revised: Toward a common metric in pediatric pain measurement. Pain 2001;93(2):173-83.

Horst J, Ellenikiotis H, Milgrom P. UCSF Protocol for Caries Arrest Using Silver Diamine Fluoride: Rationale, Indications, and Consent. J Calif Dent Assoc 2016;44(1): 16–28.

ICDAS. Available online at: www.icdas.org and https://www.icdas.org/uploads/ICDAS%20Criteria%20Document%20corrected%202013.pdf . Accessed online June 2016.

Innes NPT, Clarkson JE, Speed C, Douglass GVA, Maguire A and FICTION trial collaboration. BMC Oral Health 2013;13:25

Kassebaum NJ, Bernabe E, Dahiya M, et al. Global burden of untreated caries: A systematic review and meta regression. J Dent Res 2015;1-9.

Kiyak HA, Holt T, West RA, McNeill RW. Psychologic changes in orthognathic surgery patients: a 24-month follow-up. J Oral Maxillofac Surg 1984;42:506-512.

Larsen DL, Attkisson CC, Hargreaves WA, Nguyen, TD. Assessment of client/patient satisfaction: Development of a general scale, Evaluation and Program Planning 1979;2:197-207.

Lippert F, Hara AT, Martinez-Mier EA, Zero D. In vitro lesion rehardening and enamel fluoride update from fluoride varnishes as a function of application mode. Am J of Dent 2013;26:81-85.

Liu BY, Lo EC, Chu CH, Lin HC. Randomized trial on fluorides and sealants for fissure caries prevention. J Dent Res. 2012 Aug;91(8):753-8.

Llodra J, Rodriguez A, Ferrer B, Menardia V, Ramos T, Morato M. Efficacy of silver diamine fluoride for caries reduction in primary teeth and first permanent molars of schoolchildren: 36-month clinical trial. J Dent Res 2005;84(8):721–4.

Locker D, Allen F. What do measures of 'oral health-related quality of life' measure? Community Dentistry and Oral Epidemiology 2007;35:401-411.

Locker D, Jokovic A, Clarke M. Assessing the responsiveness of measures of oral health-related quality of life. Community Dentistry and Oral Epidemiology 2004;32:10-18.

Malden PE, Thomson WM, Jokovic A, Locker D. Changes in parent-assessed oral health-related quality of life among young children following dental treatment under general anesthetic. Community Dentistry and Oral Epidemiology 2008;36:108-117.

Marinho VCC, Worthington HV, Walsh T, Clarkson JE. Fluoride varnishes for preventing dental caries in children and adolescents. Cochrane Database of Systematic Reviews 2013, Issue 7. Art. No.: CD002279. DOI: 10.1002/14651858.CD002279.pub2

Mei ML, Chin-Man Lo E, Chu CH. Clinical Use of Silver Diamine Fluoride in Dental Treatment. Compend Contin Educ Dent 2016;37(2):93-98.

Milgrom P, Horst JA, Ludwig S, Rothen M, Chaffee BW, Lyalina S, Pollard KS, DeRisi JL, Mancl L. Topical silver diamine fluoride for dental caries arrest in preschool children: A randomized controlled trial and microbiological analysis of caries associated microbes and resistance gene expression. J Dent 2018;68:72-78.

Santamaria RM, Innes NP, Machiulskien V, Evans DJ, Alkilzy M, Splieth CH. Acceptability of different caries management methods for primary molars in a RC. International Journal of Paediatric Dentistry 2015;25:9-17.

Sonis, ST, Eilers JP, Epstein JB, LeVeque FG, Liggett WH Jr, Mulagha MT, Peterson DE, Rose AH, Schubert MM, Spijkervet FK, Wittes JP for the Mucositis Study Group. Validation of a new scoring system for the assessment of clinical trial research of oral mucositis induced by radiation or chemotherapy. Cancer 1999;85(10):2103-2113.

Tan HP, Lo EC, Dyson JE, Luo Y, Corbet EF. A randomized trial on root caries prevention in elders. J Dent Res. 2010 Oct;89(10):1086-90.

Thomson WM, Foster Page LA, Gaynor WN, Malden PE. Short-form versions of the Parental-Caregivers Perceptions Questionnaire (P-CPQ) and the Family Impact Scale (FIS). Community Dentistry and Oral Epidemiology 2013;41:441-450.

Turton BJ, Thomson WM, Foster Page LA, Saub RL, Razak IA. Responsiveness of the Child Perceptions Questionnaire 11-to 14 (CPQ11-14) for Cambodian children undergoing basic dental care. International Journal of Pediatric Dentistry 2015;21:2-8.

Warder CJ, Edelstein BL (2017). Evaluating levels of dentist participation in Medicaid. A complicated endeavor. J Am Dent Assoc 148(1):26-32.

Versloot J, Veerkamp J, Hoogstraten J. Assessment of pain by the child, dentist and independent observers. Pediatric Dentistry 2004;26(5):445-449.

Versloot J, Veerkamp JS, Hoogstraten J. Dental Discomfort Questionnaire: assessment of dental discomfort and/or pain in very young children. Community Dent Oral Epidemiol 2006;34(1):47-52.

Yee R, Holmgren C, Mulder J, Lama D, Walker D, van Palenstein HW. Efficacy of silver diamine fluoride for arresting caries treatment. J Dent Res 2009;88(7):644–7. [PubMed]

Zhi QH, Lo ECM, Lin HC. Randomized clinical trial on effectiveness of silver diamine fluoride and glass ionomer in arresting dentine caries in preschool children. Journal of Dentistry 2012;40(11):962–967. [PubMed]

Zhi Q, Lo E, Kwok A. An in vitro study of silver and fluoride ions on remineralization of demineralized enamel and dentine. Aust Dent J, 2013;58(1):50-6
