# Supplementary material for: Efficacy of Silver Diamine Fluoride on Young Children With Severe Early Childhood Caries: A Randomized Clinical Trial
Source: JAMA Pediatr. 2026 Jul 27:e262567. Online ahead of print. doi: 10.1001/jamapediatrics.2026.2567 (PMC13409122; doi:10.1001/jamapediatrics.2026.2567)
Supplement: Supplement 2. — Statistical analysis plan [file jamapediatr-e262567-s002.pdf]

Statistical Analysis Plan

Phase III RCT of the Effectiveness of Silver Diamine Fluoride in Arresting Cavitated Caries Lesions

TABLE OF CONTENTS

TABLE OF CONTENTS 2
ABBREVIATIONS 4
1. INTRODUCTION 5
2. OVERALL STUDY DESIGN AND PLAN 6
3. TRIAL OBJECTIVES AND OUTCOME MEASURES 7
3.1. Primary Outcome Measure 7
3.2. Secondary Outcome Measures 7
3.3. Tertiary Outcome Measures 8
3.4. Safety Outcome Measures 8
4. SAMPLE SIZE 9
5. RANDOMIZATION 10
6. PLANNED ANALYSES 11
6.1. Statistical Comparisons 11
6.2. Analysis Populations 11
6.2.1. Intent-to-Treat (ITT) Population 11
6.2.2. Per Protocol (PP) Population 11
6.2.3. Safety Population 11
6.2.4. Intent-to-Treat Population #2 (ITT2) - Interim Participants 11
6.2.5. Per Protocol Population #2 (PP2) - Interim Participants 12
6.3. Global Conventions and Definitions 12
6.3.1. Computing 12
6.3.2. General Statistical Methods 12
6.3.3. Listing Conventions 12
6.3.4. Table Conventions 12
6.3.5. Baseline and Study Day 13
6.3.6. Conventions for Missing or Partial Dates 13
6.4. Visit Windows 14
6.4.1. Visit and Intermediate Contact Windows 14
6.4.2. 3-Month, 6-Month, and 8-Month Contacts During COVID-19 Pandemic 14
6.5. Participant Disposition 14
6.6. Protocol Deviations 15
6.7. Demographic and Baseline Characteristics 15
6.8. Medical History 17
6.9. Prior and Concomitant Medications 17

6.10. Exposure 17
6.11. Efficacy Analyses 17
6.11.1. Primary Efficacy Hypothesis, Endpoint, and Analysis 18
6.11.2. Primary Outcome Estimand 18
6.11.3. Primary Efficacy Endpoint: Sensitivity Analyses 19
6.11.4. Secondary Efficacy Hypotheses, Endpoints, and Analyses 20
6.11.5. Tertiary Efficacy Hypotheses, Endpoints, and Analyses 21
6.12. Safety Analysis 21
6.12.1. Adverse Events 21
6.13. Subgroup Analyses for Efficacy & Safety 22
7. DATA SAFETY MONITORING BOARD 23
8. INTERIM ANALYSIS 24
9. CHANGES TO PLANNED PROTOCOL ANALYSES 25
10. REFERENCES 26
11. TABLES, FIGURES AND LISTING SHELLS 27

ABBREVIATIONS

Abbreviation	Definition
AE	Adverse Event
ANCOVA	Analysis of Covariance
CSR	Clinical Study Report
DDQ	Dental Discomfort Questionnaire
dmfs	decayed, missing, and filled surfaces
DSMB	Data Safety Monitoring Board
ECC	Early Childhood Carries
eCRF	Electronic case report form
FCS	Fully Conditional Specification
FDA	Food and Drug Administration
FIS	Family Impact Scale
GEE	Generalized Estimating Equation
ICDAS	International Caries Detection and Assessment System
IE	Intercurrent Event
ITT	Intent-to-Treat
MAR	Missing at Random
MedDRA	Medical Dictionary for Regulatory Activities
MNAR	Missing Not at Random
NIDCR	National Institute of Dental and Craniofacial Research
OHRQoL	Oral Health-Related Quality of Life
P-CPQ	Parental-Caregiver Perception Questionnaire
P-CPQ EW	Parental-Caregiver Perception Questionnaire Emotional Well-Being
P-CPQ FL	Parental-Caregiver Perception Questionnaire Functional Limitations
P-CPQ SW	Parental-Caregiver Perception Questionnaire Social Well-Being
PP	Per protocol
PT	Preferred Term
SAE	Serious adverse event
SAP	Statistical Analysis Plan
SD	Standard Deviation
SDF	Silver Diamine Fluoride
S-ECC	Severe-Early Childhood Caries
SOC	System Organ Class
TEAE	Treatment Emergent Adverse Event
TFL	Tables, Figures, Listings
UP	Unanticipated Problem

1. INTRODUCTION

There are large disparities in the access to and effectiveness of many available preventive strategies for Severe-Early Childhood Caries (S-ECC) and Early Childhood Caries (ECC), two of the disease presentations for dental caries in children, one of the most common chronic diseases of childhood. This inequity frequently results in progression of dental disease in higher risk groups to an advanced stage, during which cavitated carious lesions and/or pulpal infections develop. When primary prevention fails or is not accessible, effective treatments that are safe, simple, low-cost, and acceptable to patients are necessary to decrease disease morbidity.

The product, 38% percent Silver Diamine Fluoride (SDF; also known as Diamine Silver Fluoride- DSF) is a potentially safe, non-invasive, quick, simple to use agent that will be tested in this trial for its ability to arrest cavitated lesions with exposed dentin in primary teeth [Chu et al., 2002; Llodra et al., 2005; Tan et al., 2010; Liu et al., 2012]. SDF has been used since the 1970s in Asia as a caries arresting and anti-hypersensitivity agent, and in 2014 SDF was cleared by the Food and Drug Administration (FDA) as a medical device to manage hypersensitive teeth in adults. SDF is currently marketed in the United States as Advantage Arrest^{TM}, an FDA approved device to manage dentin hypersensitivity in adults. The effectiveness of SDF will be compared to a placebo for arrest of cavitated lesions with dentin exposed in primary teeth, an off-label use of the current FDA premarket clearance for SDF.

The primary aim of this trial is to assess the efficacy of 38% SDF applied once, to arrest cavitated lesions (caries lesions with cavities that expose dentin clinically, but with no pulp exposure, pain, mobility, or clinical signs of pulpal infection) in the primary dentition (assessed using the International Caries Detection and Assessment System-ICDAS II activity criteria) at 6-month follow-up.

This statistical analysis plan (SAP) document details the planned statistical analyses to answer the study objective(s) for Study 17-094-E. The proposed analyses are based on the contents of protocol v3.1 (dated 16 April 2019). Populations for analysis, data handling rules, statistical methods, and formats for data presentation are provided. The statistical analyses and summary tabulation described in this SAP will provide the basis for the results sections of the Clinical Study Report (CSR) for this study.

2. OVERALL STUDY DESIGN AND PLAN

This trial is a Phase III, multisite, randomized, placebo-controlled superiority trial, with two parallel groups: SDF and placebo, applied at baseline and at approximately 6 months. The primary hypothesis of the trial is that 38% SDF is superior to placebo for arresting cavitated caries lesions with dentin exposed in primary teeth when assessed approximately 6 months after initial application.

An interim analysis will be performed, where the trial may be stopped early if interim analysis results provide early determination of efficacy or futility. Further details are described in Section 8.

3. TRIAL OBJECTIVES AND OUTCOME MEASURES

The trial objectives are as follows:

- Aim 1: The primary aim of this trial is to assess the efficacy of 38% SDF applied once, to arrest cavitated lesions (caries lesions with cavities that expose dentin clinically, but with no pulp exposure, pain, mobility, or clinical signs of pulpal infection) in the primary dentition (assessed using the International Caries Detection and Assessment System-ICDAS II activity criteria, Appendix A) at 6-month follow-up.
- Aim 2: The first secondary aim is to assess the efficacy of 38% SDF applied twice, approximately 6 months apart, to arrest cavitated lesions in the primary dentition at approximately 8 months after initial application. A comparison of the effect of one application will be assessed over time at 3- and 6-month follow-ups (Sub-Aim 2a).
- Aim 3: The next secondary aim is to assess the impact of 38% SDF applied twice, approximately 6 months apart, on pain in children through use of the Dental Discomfort Questionnaire. The impact of 38% SDF on pain after a single application will be assessed at approximately 3 and 6 months (Sub-Aim 3a).
- Aim 4: The tertiary aim is to assess the effect of 38% SDF applied once (assessed at approximately 6 months after initial application) and twice, approximately 6 months apart (assessed at approximately 8 months after initial application), on family-level outcomes, assessing the impact on oral health-related quality of life (Sub-Aim 4a), and on treatment satisfaction and acceptability (e.g., associated with the change in color of treated lesions, etc.; Sub-Aim 4b). The relationship between treatment satisfaction/acceptability and other factors (e.g., family experience with ECC, oral hygiene and dietary habits) will be evaluated.

Additionally, safety will be assessed.

3.1. Primary Outcome Measure

The following primary efficacy outcome will be assessed:

- Efficacy of 38% SDF to arrest Cavitated Caries Lesions (Aim 1): Lesion arrest will be assessed for all cavitated lesions in the mouth (whether included or not in the trial, to reduce bias) using the ICDAS II system activity criteria, which categorizes lesion activity for ICDAS scores 5-6 based on dentin hardness [i.e., arrested lesions will change over time from soft-included in the trial at baseline- to hard, without an increase in ICDAS severity score (see Protocol Appendix A)]. The primary outcome will be the proportion of arrested trial lesions per child in each treatment arm. For the purposes of testing the hypothesis, the primary outcome will be measured at about 6 months post initial treatment (Aim 1).

3.2. Secondary Outcome Measures

The following secondary outcomes associated with application of 38% SDF will be assessed (*key secondary endpoints to be tested in a secondary endpoint hierarchical strategy as described in Section 6.11.4 are italicized*):

- *Efficacy of 38% SDF to arrest Cavitated Caries Lesions when applied twice, approximately 6 months apart: lesion arrest will be measured at approximately 8 months post initial treatment to assess efficacy (Aim 2).* Compare (Sub-Aim 2a) the effect of one application of SDF measured at approximately 3 and 6 months post initial treatment.
- *Child's Pain (Aim 3): The pain outcome will be defined as the proportion of children in each treatment arm experiencing toothache pain.* Additional pain outcomes will be the number of episodes of pain associated with treated teeth for each child in each arm during the follow-up period and the average recorded pain score for children in each treatment arm. The presence of pain associated with a trial tooth will be defined as a score of 1 or higher using the Dental Discomfort Questionnaire (DDQ; See Protocol Appendix B and Section 8), which a trial examiner can associate with a trial tooth during a clinical visit. The number of pain episodes is the total of all in-person visits at which a DDQ score of 1 or higher is confirmed by the trial examiner to be associated with a trial tooth. The pain score will be calculated using the DDQ.

3.3. Tertiary Outcome Measures

The following tertiary outcomes associated with the effect of 38% SDF on the Family will be assessed:

- Oral Health-Related Quality of Life (OHRQoL): OHRQoL will be assessed at baseline, 3-, 6-, and 8-month visits. Parents/legal guardians will be asked to complete the 16-item Parental-Caregiver Perception Questionnaire (P-CPQ) and 8-item Family Impact Scale (FIS) questionnaire (See Protocol Appendix C). The P-CPQ and FIS total and subscale scores are computed by summing the scores.
- Treatment Satisfaction and Acceptability: This will be assessed at baseline/24-48 hour contact/visit, and 3-, 6-, and 8-month visits using the questionnaire labeled as Family Survey (See Protocol Appendix D). Parents/legal guardians in each treatment arm will be asked about their level of satisfaction with the appearance of their children's teeth, and their overall level of acceptance and satisfaction with the treatment received. They will also be asked about their family experiences with ECC, as well as the child's brushing and dietary habits, and these items will be correlated with acceptability and satisfaction (e.g., adults having prior experience with the consequences of ECC and associated stress might be more accepting of this treatment alternative). In addition, color of treated lesions will be determined (yellow, brown, black), not as a primary indicator of lesion activity, but the change in color over time will be used for both trial arms as a measure of consequences associated with treatment assessed in the satisfaction questionnaire.

3.4. Safety Outcome Measures

Safety will be assessed through adverse events (AEs). AEs will be monitored throughout the trial. All AEs, serious adverse events (SAEs), and unanticipated problems (UPs) will be captured any time after informed consent is obtained. In addition, all AEs will be captured until 7 days after the last day of trial participation. Serious adverse events will be captured until 30 days after trial participation.

4. SAMPLE SIZE

The population will be children 12-71 months of age at the baseline caries examination attending early childhood education programs or recruited from clinics associated with the Michigan, Iowa or New York clinical trial sites.

The primary outcome of this trial is the proportion of arrested caries lesions approximately 6 months from the baseline visit (i.e., lesions that have changed from soft to hard over the trial period according to the ICDAS II activity criteria). A 10% difference in the proportion of arrested caries lesions between the SDF and placebo groups will be considered clinically significant. Prior studies in the literature of SDF using this outcome in primary cavitated lesions have shown a wide range for the proportion of arrested caries lesions for both SDF-treated and placebo-treated or untreated lesions; therefore the 10% difference in proportions was calculated assuming 55% versus 45% arrested caries lesions for SDF compared to placebo. In prior studies, an average of 4 lesions was treated per participant, with a within-participant correlation of 0.5; we are conservatively assuming 3 lesions per participant. The sample size calculations used a 2-sided test for a difference in proportions with cluster randomization (participant as the cluster) at significance level α=0.1%, with 80% power to detect a superiority difference of 10% for SDF compared to placebo. The calculations also account for the use of an early stopping rule for efficacy and futility. The interim analysis will be performed after approximately half of the sample has completed the 6-month examinations. A Lan-Demets spending function with an O'Brien-Fleming type boundary will be used, with p-values for efficacy at 0.000001709 and 0.00099938 at 50% and 100% completion, respectively. Non-binding p-values for stopping due to futility at 50% and 100% completion will be 0.2336928 and 0.00099938, respectively. With the above assumptions, the trial will require 1144 participants to be enrolled. Sample size calculations were made using East version 6.

5. RANDOMIZATION

Randomization will be allocated to SDF/Placebo in a 1:1 fashion and will be stratified by site. Randomization to 38% SDF application (treatment) or placebo (control) will be at the participant-level; all teeth within a participant will receive the same trial product. Both treatment and control will be dispensed from identical unit-dose ampules coded and labelled to ensure masking of all trial personnel. Randomization will occur after the baseline ICDAS exam is completed and it is verified that the child has eligible trial teeth.

The randomization schedule will be generated by a designated unmasked biostatistician. The Project Biostatistician will remain masked to the trial randomization until after database lock and unblinding.

Additional details can be found in the study Randomization Plan.

6. PLANNED ANALYSES

No SAP prepared in advance of the data can be definitive, and the final CSR may contain additional tables or statistical tests, if warranted by the data obtained. The justification for any such additional analyses, or modifications to any planned analyses, if applicable, will be fully documented in the final CSR.

6.1. Statistical Comparisons

All statistical comparisons will be made using a two-sided test. The interim and final analyses will be conducted using p-value thresholds of 0.000001709 and 0.00099938, respectively, for the primary analysis. The overall type I error for interim and final primary endpoint analysis is two-sided α=0.1%.

6.2. Analysis Populations

There are 5 analysis populations as described in the following sections. Participants excluded from the analysis populations and the reason for their exclusion will be listed in Appendix 16.2 of the CSR.

6.2.1. Intent-to-Treat (ITT) Population

The ITT Population will include all participants who are randomized to receive study drug. Participants will be summarized according to their randomized treatment and not the treatment they receive. The ITT Population will be used for efficacy analyses.

6.2.2. Per Protocol (PP) Population

The PP Population will be limited to subjects who complete the baseline, 3-month, and 6-month dental examinations within the originally-specified windows. The PP Population will be used for supportive analyses of efficacy.

6.2.3. Safety Population

All participants who are treated with any amount of SDF will be included in the Safety Population. Participants will be summarized according to the treatment they received. Safety analyses will be conducted using the Safety Population.

6.2.4. Intent-to-Treat Population #2 (ITT2) - Interim Participants

The ITT2 Population will include all participants randomized to receive study drug and included in the interim analysis. Participants will be summarized according to their randomized treatment and not the treatment they receive. The ITT2 Population will be used for efficacy analyses.

6.2.5. Per Protocol Population #2 (PP2) - Interim Participants

The PP2 Population will be limited to subjects included in the interim analysis who complete the baseline, 3-month, and 6-month dental examinations within the originally-specified windows. The PP2 Population will be used for supportive analyses of efficacy.

6.3. Global Conventions and Definitions

6.3.1. Computing

All data listings, summaries, figures, and statistical analyses will be generated using SAS version 9.4 or higher (SAS Institute, Cary, NC).

6.3.2. General Statistical Methods

For continuous variables, the number of non-missing observations, mean, standard deviation (SD), median, and minimum and maximum values will be presented. The minimum and maximum values will be presented with the same precision as the data. The mean and median will be presented with a precision of one decimal place more than the data. The standard deviation will be presented with a precision of two decimal places more than the data.

For categorical variables, the number/frequency and percentage within each category of the parameter will be presented. For each parameter, all categories will be shown, and for categories with a number/frequency of zero (0), only the number, without percentage, will be presented. Percentages will be presented with one decimal place.

6.3.3. Listing Conventions

In general, listings will be sorted in the following order:

- Treatment
- Participant
- Parameter
- Study Day

All data collected on the eCRFs will be presented in the data listings.

6.3.4. Table Conventions

In general, summaries of the data will be presented by treatment group with the treatment group labels displayed as follows in the table outputs:

SDF<br>(N=XX)	Control<br>(N=XX)

There is one exception, whereby baseline tables will additionally include an “Overall” column, i.e., SDF and Control combined:

SDF	Control	Overall

(N=XX)	(N=XX)	(N=XX)

6.3.5. Baseline and Study Day

Baseline values will be defined as the last available non-missing result prior to SDF administration. Assessments carried out on the day of first study drug administration (SDF application) are considered to have taken place before the SDF application if the corresponding times have not been recorded.

Study day will be calculated as the number of days from first SDF administration.

For events/assessments on or after study drug (SDF) administration:

- Study day = date of event (or date of assessment) – date of first SDF administration + 1.

For events/assessments before study drug (SDF) administration:

- Study day = date of event (or date of assessment) – date of first SDF administration.

6.3.6. Conventions for Missing or Partial Dates

In the rare case that an AE start date or time is missing, and it is unclear whether the AE is treatment emergent or not, then a conservative approach will be taken, and it will be assumed that the AE occurred after study drug administration.

If a prior or concomitant medication end date or time is missing and it is unclear whether the medication is prior or concomitant, then a conservative approach will be taken, and it will be assumed that the medication was taken on or after study drug administration.

If a medical condition end date or time is missing and it is unclear whether the medical condition is previous or ongoing, then a conservative approach will be taken, and it will be assumed that the medical condition was still active on or after study drug administration.

Where the Date of Birth is missing, the following convention will be used:

- Where the day is missing and month and year are available, the day will be completed as the 15th. For example, Date of Birth specified as --JAN1980 will be completed as 15JAN1980.
- If the day and month are missing and the year is available, the day and month will be completed as 02JUL (the 183rd day of the year). For example, Date of Birth specified as -----1980 will be completed as 02JUL1980.

All dates presented in the individual participant listings will be as recorded on the Electronic Case Report Form (eCRF).

6.4. Visit Windows

6.4.1. Visit and Intermediate Contact Windows

Visit and intermediate contact windows as initially defined in the protocol are: +/- 19 days for the 3-month in-person visit (71-110 days post-baseline), 6-month in-person visit (161-201 days post-baseline), and 8-month in-person visit (221-262 days post-baseline); +/- 10 days for the 1.5-month telephone-based intermediate contact (35-55 days post-baseline), 4.5-month telephone-based intermediate contact (125-146 days post-baseline), and 7-month telephone-based intermediate contact (200-222 days post-baseline).

6.4.2. 3-Month, 6-Month, and 8-Month Contacts During COVID-19 Pandemic

Due to the COVID-19 pandemic and associated illnesses, precautions, and shutdowns, some study subjects were unable to be seen for in-person visits within the study windows. To maintain contact with the subjects and ensure data were collected when possible, phone contacts occurred within the windows of the 3-month, 6-month, and 8-month visits. Although the dental examinations could not take place during these contacts, the questionnaire-based responses could be collected (DDQ, P-CPQ, FIS, Treatment Satisfaction and Acceptability). A subject may have two sets of data for the same 'visit' if an in-person visit subsequently occurred after the data were collected via phone. If an in-person visit occurred within the standard visit window (see *Section 6.4.1*), the data collected at the in-person visit will be used, otherwise the data collected via phone will be used.

6.5. Participant Disposition

Participant disposition will be summarized by treatment group and overall, where appropriate, for the Screened Population. The following information will be reported:

- Number of participants for the following category:
    - Screened
- Number and percentage of participants for the following categories:
    - Randomized
    - Treated
    - Not Treated
    - Completed the study
    - Discontinued the Study
    - Reasons for study discontinuation
- Number and percentage of participants included in, and excluded from, each study population together with the reasons for exclusion from the analysis set.
- Number and percentage of participants who completed / discontinued treatment, including the reasons for treatment discontinuation.
- Number and percentage of participants who met / did not meet all eligibility criteria, together with the criteria not met.

- Number and percentage of participants who failed screening prior to randomization, including the primary reason for screen failure.
- Number and percentage of participants at each site.

All disposition data will also be listed.

6.6. Protocol Deviations

A listing of protocol deviations will be provided within Appendix 16.2 of the CSR.

6.7. Demographic and Baseline Characteristics

The comparability of treatment groups with respect to participant demographics and baseline characteristics will be assessed in a descriptive manner, but no formal statistical testing will be performed. Standard continuous or categorical variable summaries will be presented by treatment group for demographic and baseline characteristics in each of the analysis populations.

Standard descriptive statistics will be presented for the demographic continuous variables, e.g.:

- Number of Children Living with You
- Number of Adults Living with You

Total counts and percentages of participants will be presented for the demographic categorical variables including:

- Age (1, 2, 3, 4, 5 years)
- Sex
- Race
- Ethnicity
- Primarily non-English spoken at home
- Study site
- Is a Household Adult Employed
- Number of Children Living with You
- Number of Adults Living with You
- Household Income

Standard descriptive statistics will be presented for the baseline characteristic continuous variables:

- Number of study teeth per subject
- Number of study lesions per subject
- dmfs (number of decayed, missing, and filled surfaces)
- dmft (number of decayed, missing, and filled teeth)
- P-CPQ FL
- P-CPQ EW
- P-CPQ SW
- FIS
- FIS PE

- FIS PF
- FIS FC
- Child's Teeth and Mouth Health
- How much is Child's Overall well-being affected by the condition of his/her teeth, lips, jaw or mouth
- How Much is Family's Daily Life Affected your child’s teeth lips, jaws or mouth

Total counts and percentages of participants will be presented for the baseline characteristic categorical variables of:

- Entry criteria met
    - Age <3 S-ECC: Any Caries
    - Age 3-5 S-ECC: upper anterior dmfs>=1
    - Age 3 S-ECC: dmfs>=4
    - Age 4 S-ECC: dmfs>=5
    - Age 5 S-ECC: dmfs> 5
- Number of study teeth per subject
- Number of study lesions per subject
- Lesion location
    - Tooth
    - Anterior / Posterior
    - Maxillary / Mandibular
    - Surface
- ICDAS severity score for enrolled lesions
- Maximum ICDAS severity score for subject
- Frequency Child's Teeth are Brushed
- Who Brushes Child's Teeth
- Child Uses Fluoride Toothpaste
- Child Uses Prescription High-Fluoride Toothpaste
- Fluoride Painted/Put on Child's Teeth
- How Often Does Child Get Sugary Drinks
- How Often Does Child Get Sugary Snacks
- How Often Does Child Drink Water
- Parent's Satisfaction in Appearance of Child's Teeth
- Child's Satisfaction in Appearance of Child's Teeth
- Have Any of Your Children Had Cavities Fixed at Hospital
- Have Any of Family or Friend's Children Had Cavities Fixed at Hospital
- Do You Have Any of Your Own Teeth
- Have You Had Cavities, Fillings, or Teeth Pulled in Last Two Years

All demographic and baseline characteristic data will also be listed.

6.8. Medical History

All medical history data will be listed.

6.9. Prior and Concomitant Medications

All prior and concomitant medications will be listed.

6.10. Exposure

The number of SDF applications administered will be summarized categorically (0, 1, 2) by treatment group for the Safety and ITT and ITT2 Populations. The number of applications administered will be listed for each participant. Missing data will not be imputed.

6.11. Efficacy Analyses

All efficacy endpoints will be analyzed using the ITT and ITT2 populations. Additional analyses will be performed on the PP and PP2 populations.

The null hypothesis is no difference between the treatment groups and the alternative hypothesis is there is a difference between treatment groups. All statistical comparisons will be made using a two-sided test. The interim and final analyses will be conducted using p-value thresholds of 0.000001709 and 0.00099938, respectively. The overall type I error for interim and final primary endpoint analysis is two-sided α=0.1%.

Several categorical variables that are to be used as covariates in the GEE model will have categories collapsed due to small sample sizes. Race and ethnicity will be combined to form a single combined variable defined as: Hispanic or Latino if a subject indicated as such for ethnicity, multiracial if more than one race was selected, Black, White, "Asian or 'American Indian or Alaska Native' or 'Native Hawaiian / Other Pacific Islander'", "'Not Reported' or Unknown". Within the 'tooth' variable, lower incisors (LL1, LL2, LR1, LR2) will be combined into a single category and LR6 will be combined with LR5 for the imputation models for the lesion arrest outcomes, and combined into upper jaw / lower jaw and anterior teeth (upper and lower central incisors, lateral incisors, and cuspids) / posterior teeth (upper and lower molars on the) variables for the imputation models for the presence of tooth pain outcomes. For the imputation models for the presence of tooth pain outcomes, child ages 1 and 2 years will be combined.

The primary method for handling missing data for 6-month and 8-month efficacy analysis will be to use a multiple imputation analysis. Missing data follow a non-monotone missing pattern. The imputation will use fully conditional specification (FCS) methods. Imputations for lesion arrest and lesion pain will be based on race/ethnicity, gender, age, trial site, initial ICDAS score, tooth location, and lesion arrest results from prior visits using a logistic regression model. Imputations for the subject-level pain score and family outcomes will be based on race/ethnicity, gender, age, trial site, maximum initial ICDAS score for the subject, a non-arrested/arrested indicator for the subject (non-arrested if any of the subject's study lesions are not arrested) at the 'current' visit or at the most recent prior visit if the 'current' visit is missing, and results at prior visits for the outcome using a predictive mean matching model. Missing at random (MAR) will be assumed.

Twenty-five imputed datasets will be created, and results from the analyses performed on each imputed dataset will be combined using standard methods via Proc MIAnalyze.

6.11.1. Primary Efficacy Hypothesis, Endpoint, and Analysis

The primary efficacy hypothesis is that 38% Silver Diamine Fluoride (SDF) will arrest cavitated caries lesions (ICDAS 5 or 6) in primary teeth, measured by a change in dentin hardness, without a change in ICDAS severity score, after a single application (measured at 6 months). This occurs as a result of the halting of the caries disease process, which occurs very soon after application of SDF due to its strong antimicrobial and remineralizing properties.

The proportion of arrested cavities at the 6-month visit will be compared between the SDF and placebo-treated groups first using a generalized estimating equation (GEE) model with a logit link for the lesion-specific binary outcome yes/no lesion arrest at the 6-month visit, using an exchangeable correlation to account for non-independence among multiple treated lesions within a participant. The GEE model will include study site as a covariate due to stratified randomization by site.

Trial teeth that cause pain due to caries, pulpal exposure, mobility, signs of pulpal infection (abscess, fistula, swelling) will be exited from the trial and referred for appropriate care (early exit strategy), and will be considered treatment failures (non-arrest) in the analysis of the primary efficacy outcome. Treatment failures from the early exit strategy and timing of the early exit are determined from the subject disposition (withdrawal from the study with reasons related to adverse event or study tooth abscess, pulpal involvement, or pain), lesion withdrawal from the study (reason for lesion withdrawal study tooth abscess, fistula, pulpal involvement, or pain), lesion examination (notes for study tooth abscess, fistula, pulpal involvement, or pain), and adverse events. In short, any lesions removed from the trial due to the “early exit strategy” will be considered treatment failures – non-arrest of the lesion and presence of pain and/or infection – and thus will not be considered to be missing data.

6.11.2. Primary Outcome Estimand

Estimand attributes for the primary efficacy analysis are defined according to the following attributes specified in ICH E9(R1) Addendum:

- **Treatment** – 38% percent Silver Diamine Fluoride (SDF) applied once at baseline.
- **Population** – Children between 12-71 months of age at baseline, up to the day the child turns 6 years old with S-ECC *[defined as: In children younger than age 3, any sign (non-cavitated or cavitated lesion) of caries in any tooth surface (i.e., most common for this age group will be on erupted smooth surfaces). From ages 3 through 5, ≥1 dmfs in maxillary anterior teeth; or a dmfs score of ≥4 (age 3), ≥5 (age 4), or ≥6 (age 5) constitutes S-ECC. Note: The “d” component of the dmfs index is defined as including cavitated and non-cavitated lesions, thus ICDAS>1)]* and with at least one SDF-target tooth with soft cavitated caries lesions extending into dentin [ICDAS 5 or 6 (Protocol Appendix A)]; and/or cavitated lesion(s) that allow for direct hardness assessment and application of SDF (microbrush applicator must fit the cavity and be able to access all exposed dentin).
- **Primary Variable** – Number of arrested cavities at the 6-month visit

- **Population level summary** – Odds Ratio comparing odds of arrested cavities in the treated group vs odds in the placebo group at month 6 visit. Population risk difference at month 6 along with its 95% confidence intervals will also be presented.
- **Intercurrent event (IE)** – Study discontinuation before 6 months visit due to pain (early exit).
- **Strategy** – The following strategy will be used to account for IEs **-** any efficacy data collected after the occurrence of the intercurrent event (early exit due to pain before visit at month 6) will be set to failure i.e. any lesions removed from the trial due to the "early exit strategy" will be considered treatment failures – non-arrest of the lesion and presence of pain and/or infection – and thus will not be considered to be missing data.

6.11.3. Primary Efficacy Endpoint: Sensitivity Analyses

Tipping Point Analyses:
A sensitivity / tipping point analysis will also be performed to evaluate the MAR assumption for the missing primary efficacy outcome (caries arrest at 6 months). A pattern-mixture approach to the missing data imputation (missing not at random, MNAR) will be applied to the logistic regression model used for imputing the missing caries arrest at 6 months outcome from the variables described above. To represent MNAR, a range of shift parameters will be used to adjust the log odds ratios for the SDF-treated group. The tipping point will be identified as the amount of shift needed to change the statistical significance of the treatment comparison using the primary efficacy analysis. The size of the shift required to change the statistical significance will be used to gauge the plausibility of the MAR assumption.

Additional Sensitivity Analysis (Visit Windows):
Due to the COVID-19 pandemic and associated illnesses, precautions, and shutdowns, some study subjects were unable to be seen for in-person visits within the study windows. The primary analysis will be repeated using wider windows after the 6-month visit, 7-month intermediate contact, and 8- month visit as follows:

- additional 10 days prior to the original window opening for all visits and intermediate contacts
- additional 10 days after the original window closure for all intermediate contacts
- 6-month visit occurred within the standard window
    - use standard windows for the 7-month contact and 8-month visit
- 6-month visit window expanded to include days +202 through +242 post-baseline in addition to the standard window
    - if the 6-month visit occurred within the expanded window, the 7-month window used 20- 40 days from the 6-month visit, and the 8-month window used 41-79 days from the 6- month visit
    - if the 6-month visit was completed but was more than 242 days post-baseline, the 7- month window used 20-40 days from the 6-month visit, and the 8-month window used 41-79 days from the 6-month visit
- 6-month visit missed
    - 7-month window 200-270 days post-baseline, 8-month window 221-300 days post-baseline

The wider window for the 6-month visit still falls within a reasonable range for a 6-month recall visit in a clinical setting. Data collected outside the allowable visit or contact windows will be treated as missing data as described above in *Section 6.11.1*.

6.11.4. Secondary Efficacy Hypotheses, Endpoints, and Analyses

The gatekeeping procedure will assess the hypotheses associated with the two key secondary endpoints in the following prescribed hierarchy. Each endpoint will be assessed using a two-sided α=0.1% significance level, however the second endpoint in the hierarchy below will be gated by the first endpoint meeting significance. Otherwise, no inference will be drawn on the second endpoint in the hierarchy below, however results with p-values will still be calculated and presented in the table outputs.

1. **Secondary Endpoint #1: Cavitated Caries Lesion Arrest at 8 months (Aim 2)**

   This secondary endpoint will be tested first. The same GEE model for binary outcomes as used for Aim 1 will be used to compare the proportion of arrested lesions at 8-months to examine Aim 2.

2. **Secondary Endpoint #2: Pain (Aim 3)**

   This secondary endpoint will be tested second only if the first secondary endpoint reaches statistical significance.

   Aim 3: At the 8-month visits, the proportion of treated lesions that have experienced pain during the follow-up period before or at the visit will be compared between the SDF and placebo-treated groups using GEE models with a logit link for the lesion-specific binary outcomes yes/no lesion pain at each visit, using an exchangeable correlation to account for non- independence among multiple treated lesions within a participant. The GEE models will include study site as a covariate due to stratified randomization by site.

<u>Other Sub-Aim Secondary Endpoint Analyses</u>:
For Sub-Aim 2a (Cavitated caries lesion arrest at 3 months), the same GEE model for binary outcomes as used for Aim 1 will be used to compare the proportion of arrested lesions at 3 months to examine Aim 2a. Sub-Aim 2a is not included in the hierarchical testing strategy.

For Sub-Aim 2b (Stability of the effect of one application of 38% SDF on caries lesion arrest between 3 and 6 months), a longitudinal (repeated measures) model for binary data that incorporates both the correlation within each subject at each time and across the time points will be used to compare the proportion of arrested caries lesions at 3 and 6 months. Study site will be included as a covariate due to stratified randomization by site.

For Sub-Aim 3a (Pain at 3 and 6 months): At the 3-, 6-month visits, the proportion of treated lesions that have experienced pain during the follow-up period before or at the visit will be compared between the SDF and placebo-treated groups using GEE models with a logit link for the lesion-specific binary outcomes yes/no lesion pain at each visit, using an exchangeable correlation to account for non- independence among multiple treated lesions within a participant. The GEE models will include study site as a covariate due to stratified randomization by site.

6.11.5. Tertiary Efficacy Hypotheses, Endpoints, and Analyses

The P-CPQ (Sub-Aim 4a), FIS (Sub-Aim 4a), and satisfaction and acceptability scores (Sub-Aim 4b) will be compared between the SDF and placebo-treated groups using analysis of covariance (ANCOVA). The models will include study site as a covariate due to stratified randomization by site. Additional ANCOVA models will consider the effects of covariates and their interactions with the treatment effect.

Changes in the color of treated lesions over time will be summarized by group.

The family's experiences with ECC, child's brushing and dietary habits, and color change will be examined as additional covariates for the analyses of satisfaction and acceptability scores.

The total number of episodes of pain associated with trial teeth per participant (calculated by adding all reports of pain throughout the trial verified by a clinical exam to be associated with a trial tooth) during the follow-up period and the DDQ scores will be summarized for the SDF and placebo treated groups. Due to the small number of participants with any pain, no statistical comparisons will be performed for the number of episodes of pain or the DDQ scores.

Additional analyses of the proportion of arrested lesions at 3-months, 6-months, and 8-months will extend the GEE models for binary outcomes to include covariates and their interactions with the treatment effect; covariates will include race/ethnicity, gender, age, trial site, initial ICDAS score, tooth location (anterior/posterior; as there are some data in the literature to suggest a difference in SDF efficacy) and number of affected teeth. Interactions of the covariates with treatment will be explored to examine the results for differential treatment effects. However, the analyses are not specifically powered to detect these interactions. Due to the small number of participants with any pain, no analyses which include covariate effects on the binary pain outcomes will be performed.

6.12. Safety Analysis

The safety analyses will be presented by the treatment received for the Safety Population.

6.12.1. Adverse Events

All reported AEs will be coded using the Medical Dictionary for Regulatory Activities (MedDRA) and summarized by System Organ Class (SOC) and Preferred Term (PT).

Analyses of AEs will be performed for those events that are considered treatment emergent AEs (TEAE), where treatment emergent is defined as: any AE with onset after the administration of study drug (SDF or placebo).

An overall summary of AEs without SOC and PT will be presented with the number and percentage of participants by treatment with any of the following types of AEs:

- TEAE
- TEAE by severity (mild, moderate, severe, life-threatening, fatal)

- TEAE by relationship to study drug (unrelated, related; where unrelated includes not related and unlikely related, and related includes possibly related, probably related, related)
- Serious TEAE
- TEAE leading to study drug discontinuation
- TEAE leading to death

In addition to the overall summary, the number and percentage of participants by treatment for each of the TEAE categories above will be summarized by SOC and PT. In these summaries, each participant will contribute only once to the incidence rate based on the most related occurrence and most severe occurrence of the AE. This is true regardless of the number of occurrences.

Summaries of TEAEs by SOC and PT will also include total number of unique AE occurrences, where unique AE occurrences include all events, regardless of whether the same participant experiences the same AE multiple times. However, if the end of a first occurrence and the start of a subsequent occurrence overlap, or the end of a first occurrence indicates “ongoing”, then this would be counted as only one occurrence.

6.13. Subgroup Analyses for Efficacy & Safety

The primary efficacy endpoint and the two key secondary endpoints (using the ITT and ITT2 populations, as well as the adverse event summaries (using the Safety population) will be performed for the following subgroups:

- Gender (Male, Female)
- Ethnicity (Hispanic/Latino, Not Hispanic/Latino)
- Race (Black/African American, White, Asian, American Indian/Alaska Native, Multiracial, Not Reported)
- Age (Binary): < 2 years vs. >=2 years of age
- Age (Chronological): 1, 2, 3, 4, 5 years of age

If any of the categories in the above variables have less than 5% of the analyses set, they may be combined with other (neighboring if ordinal) categories so that we have enough subjects in every category used in the subgroup analyses.

7. DATA SAFETY MONITORING BOARD

In addition to the PI's responsibility for oversight, trial oversight will be under the direction of a Data and Safety Monitoring Board (DSMB) composed of members with appropriate expertise in pediatric dentistry, pediatrics, statistics, ethics, and other scientific disciplines. Members will be appointed by NIDCR. The DSMB will meet at least annually to assess safety and efficacy data, trial progress, and data integrity for the trial. If safety or data integrity concerns arise, more frequent meetings may be held. The DSMB will operate under the rules of an NIDCR-approved charter that will be approved at the organizational meeting of the DSMB. The DSMB will provide recommendations to the NIDCR.

In order to maintain clinical trial integrity, there will be firewalls in place to ensure the interim analysis results will be limited to only those related experts (e.g., unmasked DSMB members, unmasked independent biostatistician) who are independent of the personnel involved in conducting and managing this study.

8. INTERIM ANALYSIS

A formal interim analysis of the primary endpoint will be conducted after approximately half of the sample has completed the 6-month examination. A Lan-Demets spending function with an O'Brien-Fleming type boundary will be used, with p-values for efficacy at 0.000001709 and 0.00099938 at 50% and 100% completion, respectively. Non-binding p-values for stopping due to futility at 50% and 100% completion will be 0.2336928 and 0.00099938, respectively.

9. CHANGES TO PLANNED PROTOCOL ANALYSES

Changes in SAP v3 from the protocol specified analyses include:

- The per protocol population definition was updated.
- Safety population was added.
- A testing strategy for key secondary endpoints was added.
- Safety analyses will be presented descriptively. Formal statistical testing for safety (adverse events) was removed.
- ITT2 and PP2 analysis populations were added (FDA request from pre-NDA meeting)
- Subgroup analyses for efficacy and safety was added (FDA request from pre-NDA meeting)

10. REFERENCES

Chu CH, Lo ECM, Lin HC. Effectiveness of silver diamine fluoride and sodium fluoride varnish in arresting dentin caries in Chinese pre-school children. J Den Res 2002;81(11):767-770. [PubMed]

Liu BY, Lo EC, Chu CH, Lin HC. Randomized trial on fluorides and sealants for fissure caries prevention. J Dent Res. 2012 Aug;91(8):753-8.

Llodra J, Rodriguez A, Ferrer B, Menardia V, Ramos T, Morato M. Efficacy of silver diamine fluoride for caries reduction in primary teeth and first permanent molars of schoolchildren: 36-month clinical trial. J Dent Res 2005;84(8):721–4.

Tan HP, Lo EC, Dyson JE, Luo Y, Corbet EF. A randomized trial on root caries prevention in elders. J Dent Res. 2010 Oct;89(10):1086-90.

11. TABLES, FIGURES AND LISTING SHELLS

The full list of all tables, figures, and listings (TFL) along with the associated prototype shells and numbering to be produced in accordance with this SAP will be maintained in a standalone TFL shell document.
