# Supplementary material for: Efficacy of Silver Diamine Fluoride on Young Children With Severe Early Childhood Caries: A Randomized Clinical Trial
Source: JAMA Pediatr. 2026 Jul 27:e262567. Online ahead of print. doi: 10.1001/jamapediatrics.2026.2567 (PMC13409122; doi:10.1001/jamapediatrics.2026.2567)
Supplement: Supplement 3. — eMethods 1. Sample size and early stopping rules eMethods 2. Multiple imputation eTable 1. Participant Dental Characteristics and Practices at Baseline eTable 2. Product Application-Number of Treatment Doses/Ampules Used [file jamapediatr-e262567-s003.pdf]

Supplemental Online Content

Fontana M, Moursi A, Gonzalez-Cabezas C, et al. Efficacy of silver diamine fluoride on young children with with severe early childhood caries: a randomized clinical trial. *JAMA Pediatr*. Published online July 27, 2026. doi:10.1001/jamapediatrics.2026.2567

eMethods 1. Sample size and early stopping rules

eMethods 2. Multiple imputation

eTable 1. Participant Dental Characteristics and Practices at Baseline

eTable 2. Product Application-Number of Treatment Doses/Ampules Used

This supplemental material has been provided by the authors to give readers additional information about their work.

© 2026 Fontana M et al. *JAMA Pediatr*.

eMethods 1. Sample size

Study population: Children 12-71 months of age at the baseline caries examination attending early childhood education programs or recruited from clinics associated with the Michigan, Iowa or New York clinical trial sites.

The primary outcome of this trial is the proportion of arrested caries lesions approximately 6 months from the baseline visit (i.e., lesions that have changed from soft to hard over the trial period according to the ICDAS II activity criteria). A 10% difference in the proportion of arrested caries lesions between the SDF and placebo groups will be considered clinically significant. Prior studies in the literature of SDF using this outcome in primary cavitated lesions have shown a wide range for the proportion of arrested caries lesions for both SDF-treated and placebo-treated or untreated lesions; therefore the 10% difference in proportions was calculated assuming 55% versus 45% arrested caries lesions for SDF compared to placebo. In prior studies, an average of 4 lesions was treated per participant, with a within-participant correlation of 0.5; we are conservatively assuming 3 lesions per participant. The sample size calculations used a 2-sided test for a difference in proportions with cluster randomization (participant as the cluster with multiple caries lesion per participant,) at significance level α=0.1%, with 80% power to detect a superiority difference of 10% for SDF compared to placebo.

The calculations also account for the use of an early stopping rule for efficacy and futility. The interim analysis will be performed after approximately half of the sample has completed the 6-month examinations. A Lan-Demets spending function with an O'Brien-Fleming type boundary will be used, with p-values for efficacy at 0.000001709 and 0.00099938 at 50% and 100% completion, respectively. Non-binding p-values for stopping due to futility at 50% and 100% completion will be 0.2336928 and 0.00099938, respectively. With the above assumptions, the trial will require 1144 participants to be enrolled. Sample size calculations were made using East version 6.

Positive interim analysis led to early trial termination for efficacy in September 2022, approved by the DSMB and NIDCR; final sample size was 830, as recorded with ClinicalTrials.gov and the FDA. Participants who were already enrolled in the trial at the time of the interim analysis decision were allowed to complete the trial.

eMethods 2. Multiple imputation

The primary method for handling missing data for 6-month and 8-month efficacy analysis used a multiple imputation analysis. Missing data followed a non-monotone missing pattern. The imputation used fully conditional specification (FCS) methods for subject-specific longitudinally-informed imputations. Imputations for lesion arrest and lesion pain were based on race/ethnicity, gender, age, trial site, initial ICDAS score, tooth location, and lesion arrest results from prior visits using a logistic regression model. Imputations for the subject-level pain score and family outcomes were based on race/ethnicity, gender, age, trial site, maximum initial ICDAS score for the subject, a non-arrested/arrested indicator for the subject (non-arrested if any of the subject's study lesions are not arrested) at the 'current' visit or at the most recent prior visit if the 'current' visit is missing, and results at prior visits for the outcome using a predictive mean matching model. Missing at random (MAR) was assumed. Twenty-five imputed datasets were created, and results from the analyses performed on each imputed dataset were combined using standard methods via Proc MIAnalyze in SAS version 9.4.

In addition, a sensitivity / tipping point analysis was performed to evaluate the MAR assumption for the missing primary efficacy endpoint (lesion arrest at 6 months). A pattern-mixture approach to the missing data imputation (missing not at random, MNAR) was applied to the logistic regression model used for imputing the missing lesion arrest at 6 months. To represent MNAR, a range of shift parameters were used to adjust the log odds ratios for the SDF-treated and/or placebo-treated group. The tipping point was identified as the amount of shift needed to change the statistical significance of the treatment comparison using the primary efficacy analysis. The size of the shift required to change the statistical significance was used to gauge the plausibility of the MAR assumption.

© 2026 Fontana M et al. *JAMA Pediatr*.

Missing data imputed at each visit:

Visit	SDF (n=976 lesions)	Control (n=1011 lesions)
3-month	248	282
6-month	347	383
9-month	438	454

A change in the significance of the SDF versus Control comparison by decreasing the probability of lesion arrest when imputing missing data in the SDF group was not possible; the SDF versus Control comparison was still significant regardless of the size of the shift in prediction model used for imputation of missing data. A change in the significance of the SDF versus Control comparison by increasing the probability of lesion arrest when imputing missing data in the Control group was achieved with a shift parameter of -3.4. The overall estimated percentages of arrested lesions for lesions with missing data were 30.5% without the shift and 87.3% with the shift. A change in the significance of the SDF versus Control comparison by decreasing the probability of lesion arrest when imputing missing data in the SDF group while simultaneously increasing the probability of lesion arrest when imputing missing data in the Control group was achieved with shift parameters of 9 for the SDF group and -1.2 in the Control group. The overall estimated percentages of arrested lesions for lesions with missing data were 43.8% without the shift and 0% with the shift in the SDF group and 30.5% without the shift and 52.0% with the shift in the Control group. Extreme shifts were required to change the significance of the SDF versus Control comparison, leading to a determination that it was unlikely that the MAR assumption was violated.

eTABLE 1

Supplemental Table 1- Participant Dental Characteristics and Practices at Baseline

Variable	Level	SDF	Control	Overall
		N (%) or N, Mean (SD), Median, Min to Max		
**Number of Study Lesions Per Child**		414, 2.36 (2.10), 2, 1 to 16	416, 2.43 (2.13), 2, 1 to 16	830, 2.39 (2.11), 2, 1 to 16
**Number of Study Teeth Per Child**		414, 2.17 (1.72), 2, 1 to 13	416, 2.23 (1.83), 2, 1 to 12	830, 2.20 (1.77), 2, 1 to 13
**d1mfs (d=ICDAS≥1)**		414, 24.80 (13.64), 23, 2 to 72	416, 24.56 (13.86), 22, 3 to 83	830, 24.68 (13.74), 22, 2 to 83
**d3mfs (d=ICDAS≥3)**		414, 13.00 (11.01), 10, 1 to 66	416, 12.41 (10.19), 10, 1 to 59	830, 12.70 (10.60), 10, 1 to 66
**d5mfs (d=ICDAS≥5)**		414, 10.20 (10.10), 7, 1 to 66	416, 10.00 (9.09), 7, 1 to 58	830, 10.10 (9.60), 7, 1 to 66
**Frequency Child's Teeth Brushed**	(missing)	13	6	19
	2x/day	269 (67.1%)	264 (64.4%)	533 (65.7%)
	1x/day	107 (26.7%)	109 (26.6%)	216 (26.6%)

© 2026 Fontana M et al. *JAMA Pediatr*.

	Every other day	15 (3.7%)	18 (4.4%)	33 (4.1%)
	Seldom	9 (2.2%)	17 (4.1%)	26 (3.2%)
	Never	1 (0.2%)	2 (0.5%)	3 (0.4%)
**Brushing: Child Alone**	(missing)	13	6	19
	No	265 (66.1%)	259 (63.2%)	524 (64.6%)
	Yes	136 (33.9%)	151 (36.8%)	287 (35.4%)
**Brushing: Child with Adult Supervision**	(missing)	13	6	19
	No	173 (43.1%)	194 (47.3%)	367 (45.3%)
	Yes	228 (56.9%)	216 (52.7%)	444 (54.7%)
**Brushing: Parent/Adult**	(missing)	13	6	19
	No	215 (53.6%)	219 (53.4%)	434 (53.5%)
	Yes	186 (46.4%)	191 (46.6%)	377 (46.5%)
**Child Use of Fluoride Toothpaste**	(missing)	13	6	19
	No	41 (10.2%)	36 (8.8%)	77 (9.5%)
	Unsure	41 (10.2%)	57 (13.9%)	98 (12.1%)
	Yes	319 (79.6%)	317 (77.3%)	636 (78.4%)
**Fluoride Painted/Put on Their Teeth by Dentist/Doctor**	(missing)	15	6	21
	No	222 (55.6%)	232 (56.6%)	454 (56.1%)
	Unsure	34 (8.5%)	42 (10.2%)	76 (9.4%)
	Yes	143 (35.8%)	136 (33.2%)	279 (34.5%)
**How Often Child Gets Sugary Drinks?**	(missing)	13	6	19
	Three or more times a day	75 (18.7%)	94 (22.9%)	169 (20.8%)
	One or two times a day	212 (52.9%)	203 (49.5%)	415 (51.2%)
	Weekly	84 (20.9%)	72 (17.6%)	156 (19.2%)
	Monthly	15 (3.7%)	29 (7.1%)	44 (5.4%)
	Never	15 (3.7%)	12 (2.9%)	27 (3.3%)
**How Often Child Gets Sugary Snacks?**	(missing)	13	7	20
	Three or more times a day	34 (8.5%)	46 (11.2%)	80 (9.9%)
	One or two times a day	232 (57.9%)	216 (52.8%)	448 (55.3%)

© 2026 Fontana M et al. *JAMA Pediatr*.

	Weekly	113 (28.2%)	125 (30.6%)	238 (29.4%)
	Monthly	18 (4.5%)	19 (4.6%)	37 (4.6%)
	Never	4 (1.0%)	3 (0.7%)	7 (0.9%)
**How Often Child Drinks Water?**	(missing)	15	7	22
	Daily	270 (67.7%)	265 (64.8%)	535 (66.2%)
	Weekly	24 (6.0%)	29 (7.1%)	53 (6.6%)
	Monthly	12 (3.0%)	7 (1.7%)	19 (2.4%)
	Never	93 (23.3%)	108 (26.4%)	201 (24.9%)
**Child Had Cavities Fixed in Hospital Before**	(missing)	14	8	22
	Do not know	2 (0.5%)	5 (1.2%)	7 (0.9%)
	No	331 (82.8%)	346 (84.8%)	677 (83.8%)
	Yes	67 (16.8%)	57 (14.0%)	124 (15.3%)

4) eTABLE 2

Supplemental Table 2. Product Application-Number of Treatment Doses/Ampules Used

Treatment Doses Visit: # of ampules used (BL: baseline)	SDF	Control	Overall
**BL: 1**	103 (24.9%)	103 (24.8%)	206 (24.8%)
**BL: 1, 6M: 1**	294 (71.0%)	287 (69.0%)	581 (70.0%)
**BL: 1, 6M: 2**	9 (2.2%)	8 (1.9%)	17 (2.0%)
**BL: 1, 6M: 3**	0 (0%)	1 (0.2%)	1 (0.1%)
**BL: 2**	5 (1.2%)	7 (1.7%)	12 (1.4%)
**BL: 2, 6M: 1**	3 (0.7%)	6 (1.4%)	9 (1.1%)
**BL: 3**	0 (0%)	1 (0.2%)	1 (0.1%)
**BL: 3, 6M: 1**	0 (0%)	2 (0.5%)	2 (0.2%)
**BL: 6, 6M: 1**	0 (0%)	1 (0.2%)	1 (0.1%)

© 2026 Fontana M et al. *JAMA Pediatr*.
