# Supplementary material for: Efficacy of Silver Diamine Fluoride on Young Children With Severe Early Childhood Caries: A Randomized Clinical Trial
Source: JAMA Pediatr. 2026 Jul 27:e262567. Online ahead of print. doi: 10.1001/jamapediatrics.2026.2567 (PMC13409122; doi:10.1001/jamapediatrics.2026.2567)
Supplement: Supplement 4. — Data sharing statement [file jamapediatr-e262567-s004.pdf]

Data Sharing Statement

Fontana. Efficacy of Silver Diamine Fluoride on Young Children With Severe Early Childhood Caries. *JAMA Pediatr*. Published July 27, 2026. doi:10.1001/jamapediatrics.2026.2567

Data
Additional Information: ClinicalTrials.gov Identifier:NCT03649659
Data available: Yes
Data types: Deidentified participant data, Data dictionary
How to access data: De-identified data upon request and completion of a data use agreement. Interested researchers should send data requests for consideration and approval to the corresponding author, mfontan@umich.edu
When available: With publication

Supporting Documents
Document types: None

Additional Information
Who can access the data: Researchers whose proposed use of the data has been approved.
Types of analyses: For any purpose that does not overlap with the analyses planned by the study team
Mechanisms of data availability: After approval of a proposal and a signed data use access agreement
